# Ultrasonic and conversion-based inline fouling measurements for continuous emulsion copolymerisation of vinyl acetate in a tubular reactor

**DOI:** 10.1038/s41598-024-54321-4

**Published:** 2024-02-19

**Authors:** Sören Rust, Marco Osenberg, Thomas Musch, Werner Pauer

**Affiliations:** 1 Institute for Technical and Macromolecular Chemistry, University of Hamburg, Bundesstraße 45, 20146 Hamburg, Germany; 2 Chair of Electronic Circuits, Ruhr-University Bochum, Universitätsstraße 150, 44801 Bochum, Germany

**Keywords:** Emulsion polymerization, Continuous emulsion polymerisation, Tubular reactor, Vinyl acetate, Fouling, Inline measurements, Chemical engineering, Polymer chemistry

## Abstract

One of the serious challenges for the implementation of continuous emulsion polymerisation is their significant fouling. The elucidation of time-dependent fouling processes and the development of inline analysis methods for fouling mass quantification are crucial to making progress in this area. Inline-sensor concepts based on ultrasonic measurements as well as residence time and conversion analysis were investigated regarding their suitability for the detection of time-dependent fouling formation and compared with gravimetrical results in order to validate their precision. Both a set-up using a turnover analysis for determination of losses in reaction volume by fouling and an ultrasound-based measurement system detecting deposit-caused changes by evaluating the average sound velocity could be used as suitable sensor concepts. The accuracies of both sensors are below 10% deviation to fouling references.

## Introduction

Fouling is a major challenge for various chemical processes on industrial scale. As cleaning of large-scale plants is time and cost intensive, it is advantageous to gain knowledge about possible occurring fouling during the reaction and how to avoid it to obtain an efficient process. The full understanding of fouling mechanisms is challenging and needs a lot of further research. Some empirical fouling prevention strategies were established and patented. Additives are used to reduce or avoid fouling^1–7^. For example polyoxyalkenes could be used for fouling reduction in solution polymerisation of ethylene co- and homopolymers^6^ or alkyl and aryl phthalates prevent fouling for the polymerisation of acrylates, methacrylate or acrylic acid^7^. In different applications coatings of the equipment were described as solution for fouling reduction^8–11^. Monolayer coatings with polyvinyl alcohol and the disodium salt of bisphenol A are advantageous for the polymerisation of vinyl halides or vinylidene monomers^8^ or water dispersion coatings of alumina oxalyl bis(benzylidene)hydrazide^9^ respectively aqueous selenous acid^10^ can be used for vinyl chloride polymerisation. Besides chemical concepts for fouling reduction there are mechanical concepts as well which are using special reactor concepts^12–17^. McFadden et al.^13^ reduced fouling during continuous polymerization by highly precise control of monomer streams and process parameters. Lowell et al.^14^ optimized the geometry of the reactor to reduce fouling for gas phase polymerisation. Carvalho et al.^17^ reported that an oscillatory flow reactor causes a fouling free continuous emulsion polymerization at lab scale. Moreover, for some specialized applications the addition of comonomers to the recipe is described like for Kinetic Hydrate Inhibitor Formulations^18^. Another approach instead of preventing fouling is the optimization of cleaning concepts, so the efficiency could be increased by efficient cleaning. Olefinic polymer deposits for example can be efficient cleaned by circulating high boiling aromatic hydrocarbon solvents in the reactor and afterwards recovering them by flash separation^19^. Haruyama^20^ developed a mechanical cleaning strategy of polymeric fouling by polymerizing in the gap between two tempered tubes built into each other, which can be tempered independently of each other for cleaning, so that the reaction gap is closed by material expansion or contraction and removes polymer residues^20^. Saikhwan et al.^21^ reported temperature and pH conditions where cleaning of non-cross-linked acrylate-styrene copolymers is easily done by taking advantage of the swelling behavior.

Regarding fouling kinetics or mechanisms there are less publications. For wastewater treatment there are precise mechanistic studies by Ekowati et al.^22^ who reported that cationic polymers cause fouling on reverse osmose membranes. It was possible to predict the time dependent fouling masses and distinguish between reversible fouling that could be removed by chemical cleaning and irreversible fouling that influenced the efficiency of the membranes permanently^22^. Regarding homogeneous polymerisation reactions Deglmann et al.^23^ reported the fouling processes in the free radical polymerisation of N-vinyl pyrrolidone are caused by radical transfer reactions leading to terminal double bonds which are crosslinked afterwards. This crosslinking increases the molecular weight significantly and leads to deposit formation^23^. Neßlinger et al.^24^ performed solvent polymerisation of *N*-vinyl pyrrolidone and evaluated the fouling masses. They investigated the influences of coated reactor surfaces on fouling masses and their suitability for fouling prevention^24^.

The kinetics of emulsion polymerization are described in multiple publications by different research groups, for example Smith and Ewart^25^, Chern^26^, Gilbert^27^, Schork and many others^27–35^. The state of the art regarding modelling development of particle size distributions in emulsion polymerisation and discussing coagulation is well described by Vale et al.^36^. Especially continuous emulsion polymerisation in tubular reactors and approaches for modelling them are discussed here^36^. The fouling behaviour during emulsion polymerisation of vinyl acetate is described in literature as well. Different publications are discussing some parts of this topic^17,37–39^. Carvalho et al.^17^ performed continuous emulsion copolymerisation of vinyl acetate and *n*-butyl acrylate in a tubular reactor and found that fouling caused problems which could be solved by oscillating pulsed flow control and internal sieve plates in the reactor. The oscillating flow results in short periods of turbulent flow control and prevents fouling by high shear rates while the internal sieve plates improve radial mixing and reduce side reactions^17^. Emulsion copolymerisation of vinyl acetate and vinyl esters were investigated regarding to their fouling on heated or cooled surfaces and compared with commercial polyvinyl acetate dispersions. The influences of temperature and surface modifications on fouling masses show that higher temperatures lead to higher fouling masses. For surface modifications no general trend could be observed as it is highly dependent on the other process parameters^37–39^. Hohlen et al.^38^ compared fouling of reactive emulsion polymerisation systems with fouling of non-reactive polymer dispersions. Reactive polymerisation systems cause much higher fouling masses than non-reactive so polymerisation fouling is the major part. Moreover, the morphology of the fouling is given by the pathway of formation so the dominant fouling process can be concluded optically from the fouling morphology^38^.

On the field of fouling quantification technologies there are different approaches described^40–44^. For example Böttcher et al.^40^ reported an inline measurement technique for monitoring fouling masses during emulsion copolymerisation of *n*-butyl acrylate and methyl methacrylate using a quartz crystal microbalance with dissipation monitoring. They reported two different pathways for heat-transfer fouling, the first one leading to thin fouling films with no further growth after formation and the other resulting in continuously growing thick fouling^40^. In fouling intense polymerisation processes it is commonly practiced to detect the changes in the reactor weight^41,42^. At low fouling masses this method is not as precise as a quartz crystal microbalance but for stronger fouling a quartz crystal microbalance does not work robust enough so the weighting of the whole reactor or parts of them is more suitable. Osenberg et al.^43,44^ proved in previous work the general suitability of ultrasonic fouling detection for polymerisation processes as the solvent polymerisation of polyurethanes^43,44^.

Concluding these publications there is much published work about fouling reduction or prevention but less literature which addresses scientific approaches to fouling processes. Most of the scientific publications are focussed on mechanistic influences or temperature effects so there are no publications addressing time-resolved fouling behaviour for the continuous emulsion polymerisation process of vinyl acetate copolymers. This work investigates the fouling behaviour of continuous emulsion polymerisations of vinyl acetate vinyl ester copolymers and develops and ultrasonic measurement approach as well as and conversion based method for inline fouling quantification.

## Exp. section

**Figure 1:**
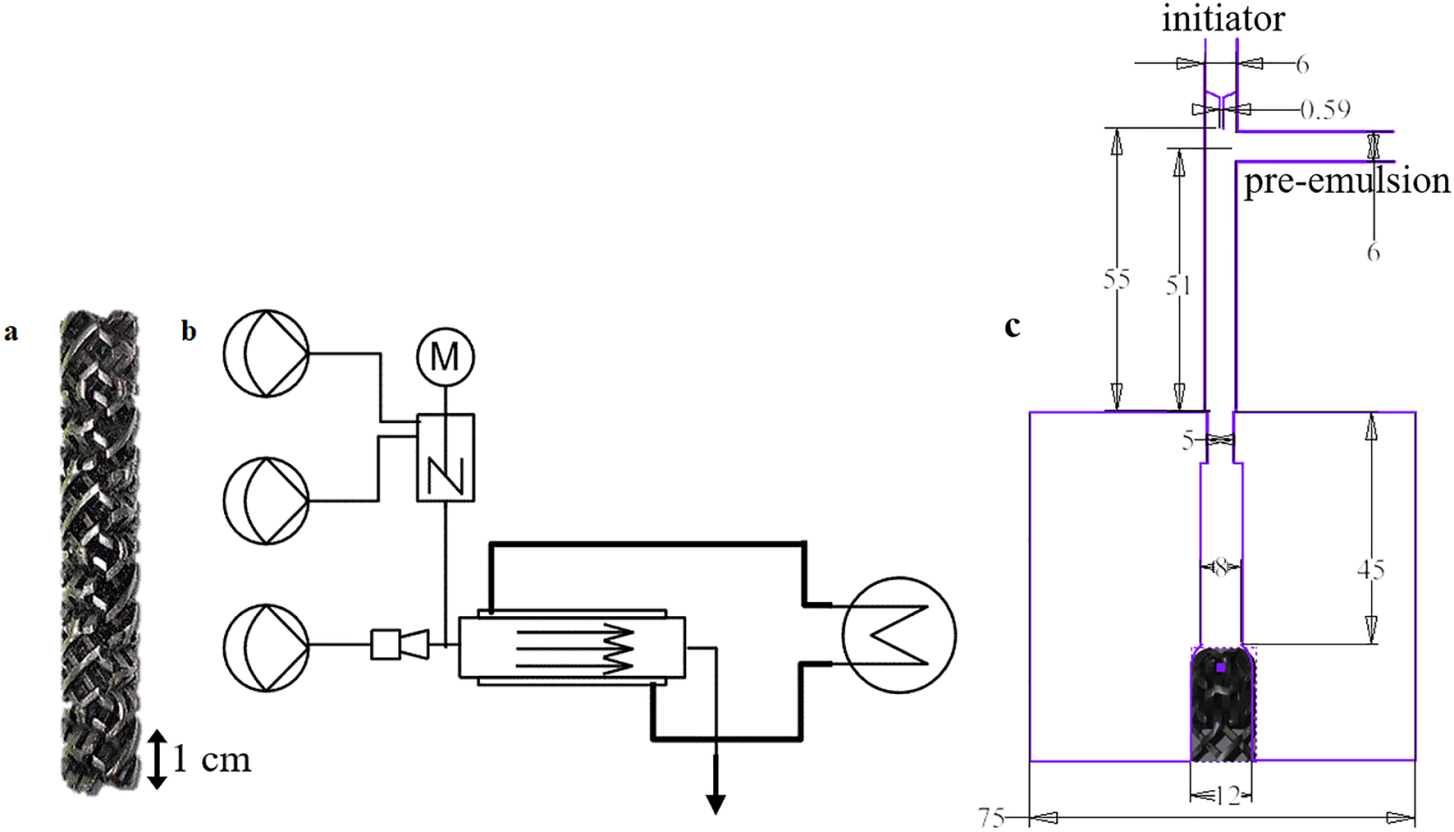
(**a**) Picture of the static mixers type CSE-X, (**b**) schematic structure of the reactor plant, (**c**) detailed plan of the initiator nozzle.

The experiments were carried out in a tubular lab reactor set-up of the type Fluitec ContiPlant LAB^®^ with CSE-X static mixing elements (Fig. 1a). The half-shell reactor has a channel diameter of d = 12 mm and filled with CSE-X static mixing elements a resulting volume of V = 45.5 mL. Temperature control of reactor and ultrasonic measurement cell was achieved by a cryostat of the type Julabo FP50 tempering the double jacket to a constant inlet temperature, closed loop temperature controlled at the jacket inlet (Fig. 1b).

The experimental procedure and recipe were chosen similar as in previous work and are described detailed here for better understanding with the included differences for this task^45^. The dosing was achieved by three solenoid-driven diaphragm metering pumps ProMinent^®^ Gamma. The monomers, vinyl acetate and vinyl neodecanoate (VeoVa10^®^), (ProMinent gamma/4-1, stroke volume 0.13 mL/stroke, dosing flow 0.05 g/s) and the emulsifier solution containing poly(vinyl alcohol), Ascorbic acid and ammonium iron(III)sulphate (ProMinent gamma/5, stroke volume 0.16 mL/stroke, dosing flow 0.15 g/s) were pre-emulsified in a cylindrical CSTR equipped with a magnetic stirrer (*d* = 25.4 mm, *V* = 20 mL, *d*_stirrer_ = 18 mm, 600 rpm) and the initiator flow containing *tert*-butyl hydroperoxide (ProMinent gamma/4, stroke volume 0.03 mL/stroke, dosing flow 0.01 g/s) was added 55 mm upstream and centered to the reactor inlet via a capillary nozzle (inner diameter 1 mm) (Fig. 1c)^45^.

All chemicals were used without further purifying. Vinyl acetate, vinyl neodecanoate and Mowiol 4–88 (molecular weight ~ 31 kDa, Degree of hydrolysis 88%) were purchased in technical grade from Wacker Chemie AG, Burghausen. Ascorbic acid and ammonium iron(III) sulphate dodecahydrate were purchased in analysis grade from Sigma Aldrich. *tert*-Butyl hydroperoxide was purchased as an aqueous solution (c = 70%(w/w)) from Sigma Aldrich^45^.

For the monomer feed a vinyl acetate-vinyl neodecanoate (VeoVa10^®^) comonomer stock system was used for emulsion polymerisation, with 10 wt% VeoVa10^®^ based on total monomer content. Polymerisation were carried out with a monomer content of 23.8 wt%. Polyvinyl alcohol was used as an emulsifier in weight proportions of 8 wt% based on monomer. The initiation was carried out by a redox initiator system consisting of *tert*-butyl hydroperoxide, ascorbic acid and ammonium iron(III) sulphate dodecahydrate in a molar ratio of 1:1:0.03. The initiator was used in weight proportions of 1 wt% based on monomer^45^. The mean hydrodynamic residence time of the reactor is approximately four minutes which is comparable to the time for reaching full conversion. The runtime of the experiments was varied between 20 and 180 min.

**Figure 2:**
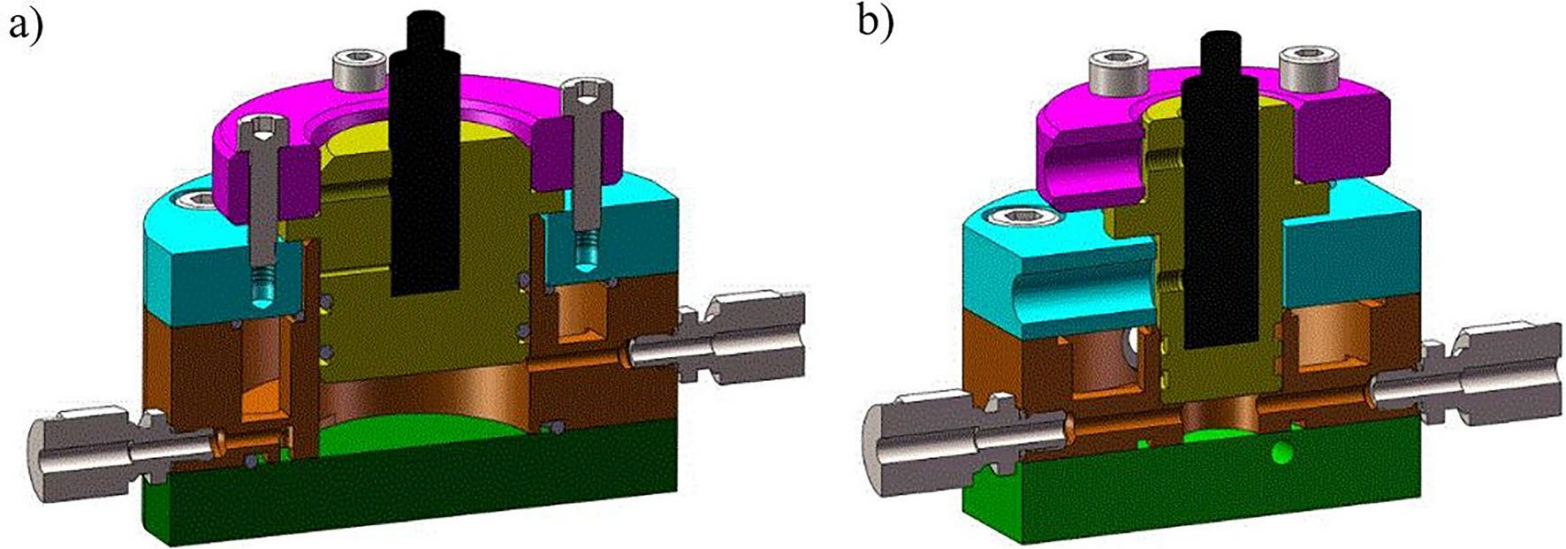
CAD models of the ultrasonic measurement cells, the coupling path (yellow) is made of PEEK, all other parts are made of stainless steel 1.4404, (**a**) measurement cell with large volume, (**b**) optimized measurement cell with smaller volume.

Two ultrasonic measuring cells were developed by the chair of electronic circuits at the Ruhr-University Bochum. To favor fouling to settle in the measuring cell, the flow profile was designed in a way that allows for regions with small flow velocities, since previous work has shown that higher flow velocities reduce fouling^44^. The measurement cells were made of 1.4404 stainless steel. To couple the ultrasonic signals into the emulsion phase, a coupling path (Fig. 2, yellow part) made of polyetheretherketone (PEEK) is used, since the acoustic impedance (AI) of PEEK better matched with the AI of the emulsion than the AI of stainless steel. Thus, more energy can be coupled into the sensing element, resulting in a higher dynamic range. The measurement cells are cylindrical in shape, with different dimensions leading to different dwell times and dead volumes. The first measurement cell (Fig. 2a) has a height of *h*_c_ = 10 mm and a radius of *r*_c_ = 15 mm concluding to a cell volume of *V*_c_ = 7.0 mL with large, fouling intense dead zones. The second measurement cell (Fig. 2b) has an optimised geometry with less dead zones. The dimensions are a height of *h*_c_ = 7.5 mm and a radius of *r*_c_ = 8 mm concluding to a cell volume of *V*_c_ = 1.5 mL. A cell with higher volume would be more sensitive to early stages of the deposit formation because of the low flow velocities but will not represent the situation in the reactor well. Concluding the second measurement cell with smaller volume was considered and detailed investigated. A sectional view of the computer aided design (CAD) models is shown in Fig. 2.

**Figure 3:**
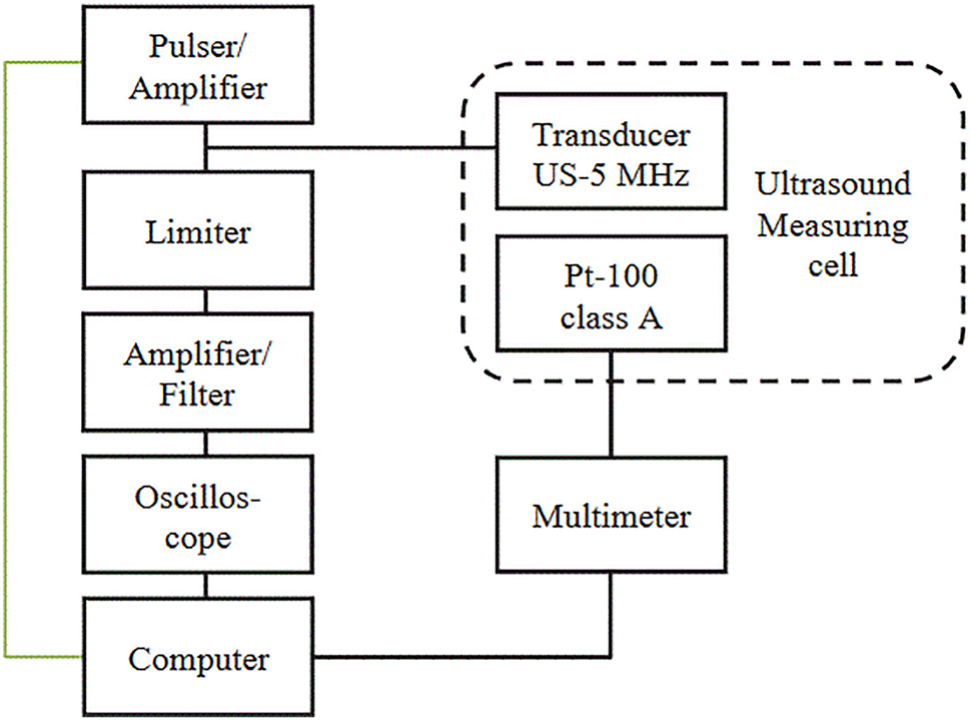
Block diagram of the ultrasonic measurement set-up.

An ultrasonic transducer with a center frequency of *f*_M_ = 4.8 MHz (6 mm aperture diameter, 4.02 MHz bandwidth, Olympus Corp., USA, model V310-SM) was used for measurement because the emulsion causes high signal attenuation at higher frequencies. At the selected center frequency, still a best spatial resolution is ensured with sufficient signal-to-noise ratio. The ultrasonic transducer is excited with an experimental MOSFET output stage, this generates a pulse with a pulse length of *t*_PA_ = 100 ns and a voltage of *U*_PA_ = 50 V. To decouple the reflection signals from the excitation a passive decoupling is used. For protection of the amplifiers and analog filters, an analog voltage limiter is applied to limit the received signals to *U*_Lim_ = 0.7 V, ensuring that the receiving circuitry is not damaged. The signals are filtered with a 6th order bandpass filter with a lower cutoff frequency of *f*_u_ = 0.5 MHz and an upper cutoff frequency of *f*_o_ = 20 MHz. To digitize the signals, an oscilloscope (Rohde & Schwarz GmbH, Germany, model RTO 1004) with a sampling frequency of *f*_sp_ = 500 MSps is used. The measurement set-up is shown graphically as a block diagram (Fig. 3).

To enhance the signal to noise ratio, the mean value is formed from 25 individual measurements and a digital band pass filter is used to suppress any interference that is outside the useful frequency range. To determine reflection times and signal powers, the envelope of the time signal is evaluated. For this purpose, a Gaussian fit is performed with the Curve Fitting Toolbox of Matlab™ in the calculated in-coupling and out-coupling reflection time sections. The Gaussian fit provides a good representation of times and signal powers, since many measurement points are incorporated for the fit. From these data, the average sound velocity (ASV) of the phases in the measurement cell can now be evaluated over the coupling time *t*_1_ and the back-wall reflection time *t*_2_ (Eq. 1).

1 $$c_{{ASV}} = \frac{{h_{{\text{c}}} }}{{t_{1} - t_{2} }}$$

All fouling masses are given as fouling masses referenced to the weight of the static mixers in the reactor where the fouling mainly occurs (mg_fouling_/g_mixer)_. Conversions *X*(t) were calculated as the weight ratio between formed polymer *w*_P_ and total initial monomer content *w*_M_ (Eq. 2). The polymer content was measured by microwave gravimetry of the product dispersions.

2 $$X(t)=\frac{{w}_{{\text{P}}}(t)}{{w}_{{\text{M}}}(t=0)}$$

For gel permeation chromatography (GPC) a set-up using tetrahydrofuran as eluent with a flow rate of 1 mL/min was used. The set-up contained in order of use a Knauer K-4002 2- channel Degasser, a FLOM Intelligent Pump AI-12–13 and a Knauer Smartline 3800 Autosampler with 20 µL sample loop. As columns one PLgel 10 µm Guard followed by two PLgel 10 µm MIXED-B columns by Agilent Technologies were used. The detection was performed by a Schambeck SFD GmbH RI 2000 detector measuring refractive index. All measurements were calibrated with linear polystyrene standards of different molecular weights, so the obtained molecular weights are qualitative and not quantitative.

## Results

For the development of inline-sensor concepts to monitor time-dependent fouling formation it is in the first step necessary to establish a robust and reproducible polymerisation which results in reproducible fouling masses. In the second step the time and space resolved fouling deposition can be quantified. In further experiments these data will be used to evaluate possible sensor concepts regarding their suitability and precision.

### Investigation of a suitable polymerisation

**Figure 4:**
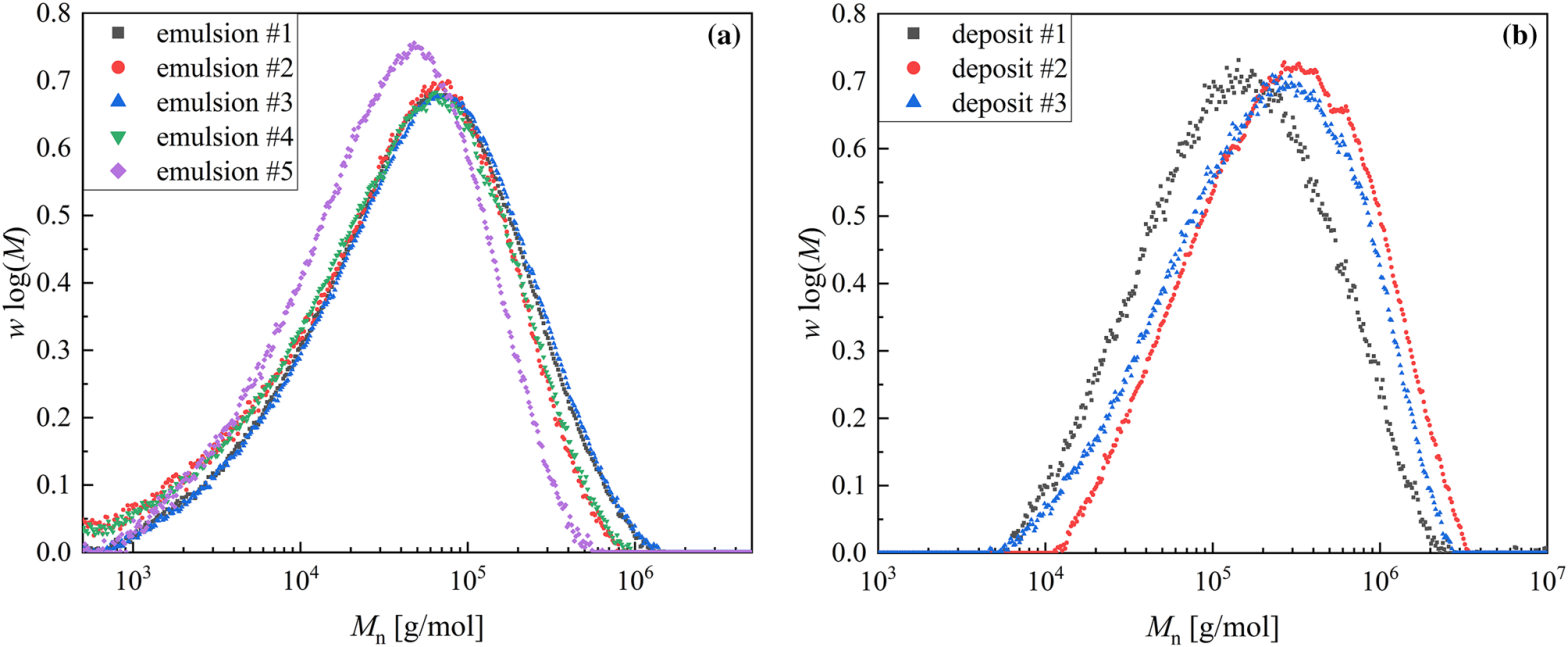
Plot of the qualitative molecular weight distribution of emulsion polymers from five different reproduction experiments (**a**) and deposits from three of this reproduction reactions (**b**) at same conditions at 20 °C to confirm reproducibility.

An emulsion copolymerisation of vinyl acetate and vinyl neodecanoate was chosen as test system for the fouling detection. The polymer properties of the products from reproduction experiments were investigated to validate the reproducibility of the reaction. For this purpose, the molecular weights of the emulsion polymers and the deposit of three different reactions under same conditions were determined by qualitative GPC and compared (Fig. 4). Moreover, the molecular weights of the emulsion polymers of two additional reactions from sensor testing were included.

Very similar molecular weights for all emulsion polymers as well as all deposits were obtained, with the molecular weight of the emulsion polymers always being lower than that in the fouling, so the polymerisation process is reproducible and the polymeric products are comparable.

**Figure 5:**
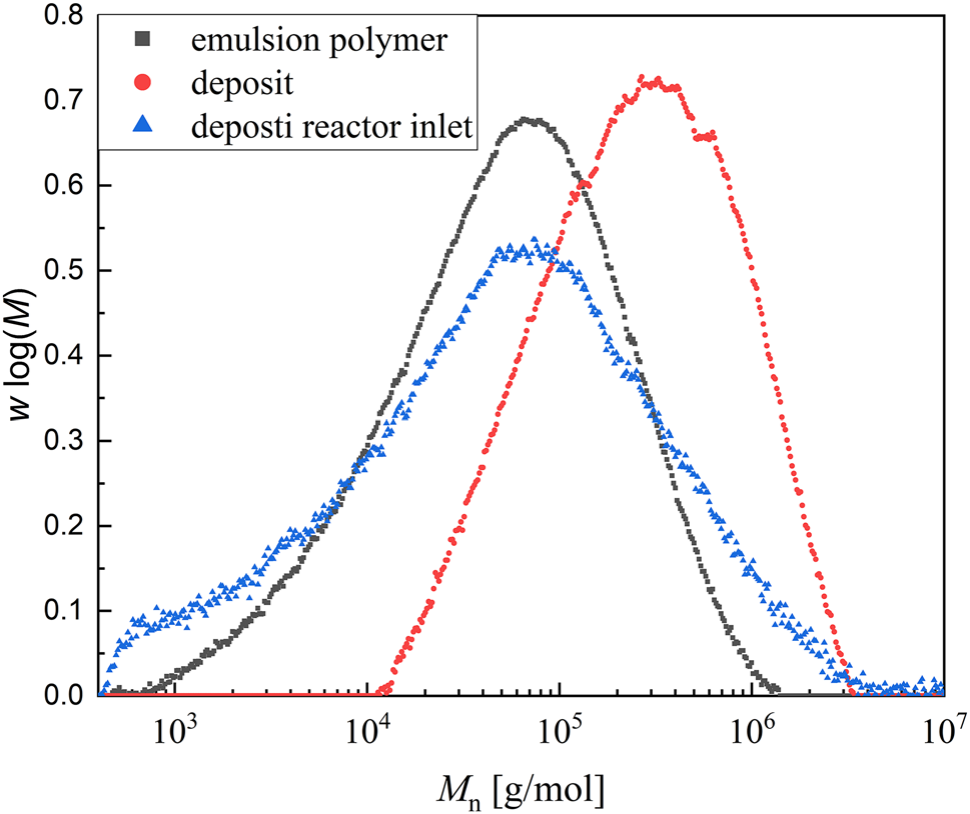
Plot of the molecular weight distribution of emulsion polymer, deposit and deposit at the reactor inlet for comparison of the polymeric properties for an experiment at 20 °C.

The molecular weight of the emulsion polymer, the deposit and the deposit in the first part of the reactor were compared as well. The deposit in the first part of the reactor was investigated as the conversion there is lower and a broader molecular weight distribution is expected, if the deposits are caused by polymerisation fouling (Fig. 5).

The molecular weights of the emulsion polymers are significantly lower than those of the deposits (Fig. 5), indicating that the polymeric properties of the two phases do not match. In particular, the molecular weight distribution of the fouling at the reactor inlet is of interest, as it is much broader than the others. It ranges from very low molecular weights to the high molecular weights of the deposits. This indicates that polymerisation fouling is the dominant process, since at the reactor inlet at low radical concentrations significant monomers as well as oligomers remain in the deposit, i.e., polymerisation takes place in a film on the reactor wall and no sedimented particles are responsible for the fouling. The increased molecular weights in the deposits are presumably since the initiation takes place in the aqueous phase and therefore, due to the lower surface-to-volume ratio, fewer radicals enter the film on the reactor wall than the micelles, so that termination occurs later there.

The statistical deviation of fouling deposition was determined by replication tests. For this purpose, seven tests were carried out under conditions that were as identical as possible and the deviation was calculated. The statistical component results in an average deviation of 12% which indicates the range of scattering including measurement errors.

### Time and space resolved fouling deposition

**Figure 6:**
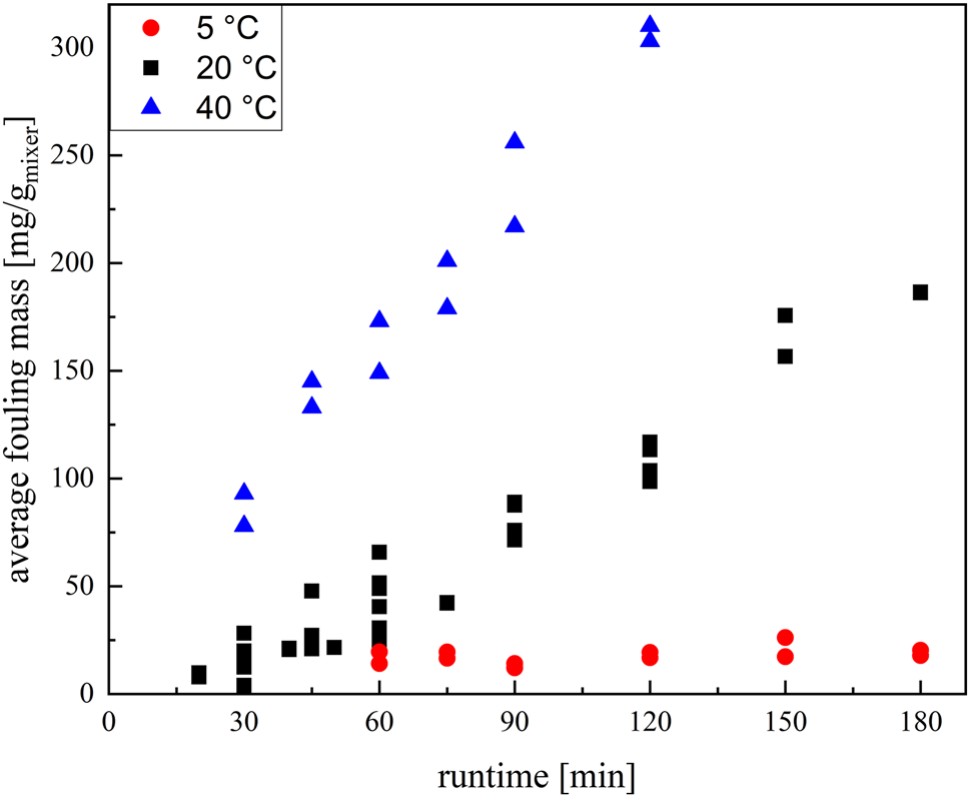
Plot of the mean fouling masses of the total reactor over the runtime of the experiment at different temperatures.

First, the average fouling masses of the entire reactor were determined gravimetrically and their development over time was investigated. Since it is known that the temperature also exerts an influence on the fouling^45^, the influences of the temperature are investigated as well (Fig. 6). This data could be used later for calibrating the sensors.

The overall fouling in the reactor is linear, i.e. the formation of initial fouling neither inhibits the fouling as would be expected from a reduction in the surface energy, nor does the deposit formation increase due to the more uneven surface. The linear correlations of the time-dependent fouling exist for all investigated temperatures (Fig. 6). Only the slope of the linear function is different due to the changed temperature and describes the temperature dependence of the fouling. This indicates that nothing changes mechanistically in the selected temperature range, since a mechanistic change would usually be accompanied by a different time-related behaviour. For the fouling masses at 5 °C the slope of linear correlation is close to zero and in the shown time-interval the offset in fouling caused by dead zones in the reactor dominates the time-dependent fouling trend.

**Figure 7:**
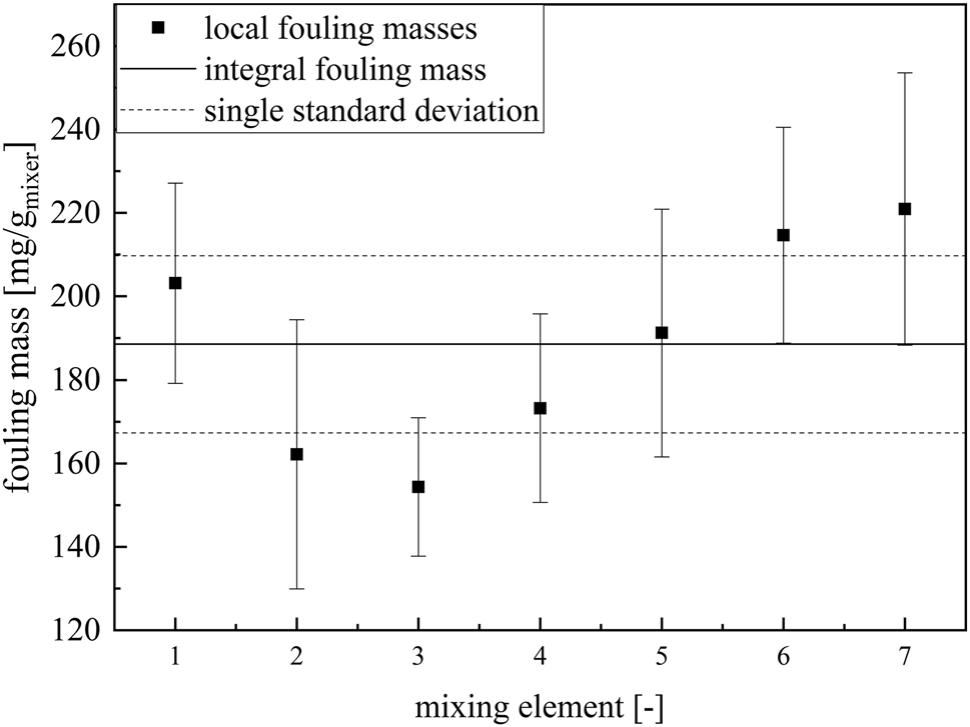
Plot of the mean fouling masses of the entire reactor and the fouling masses of the individual static mixers at a runtime of 120 min and a temperature of 20 °C.

The reaction progresses along the reactor and the fouling is depending on the conversion^45^. It is to be expected that higher fouling masses occur at the reactor outlet than at the inlet, since higher conversions are achieved there. For this purpose, the fouling masses of the individual static mixers were compared with the mean values of the overall reactor (Fig. 7).

**Figure 8:**
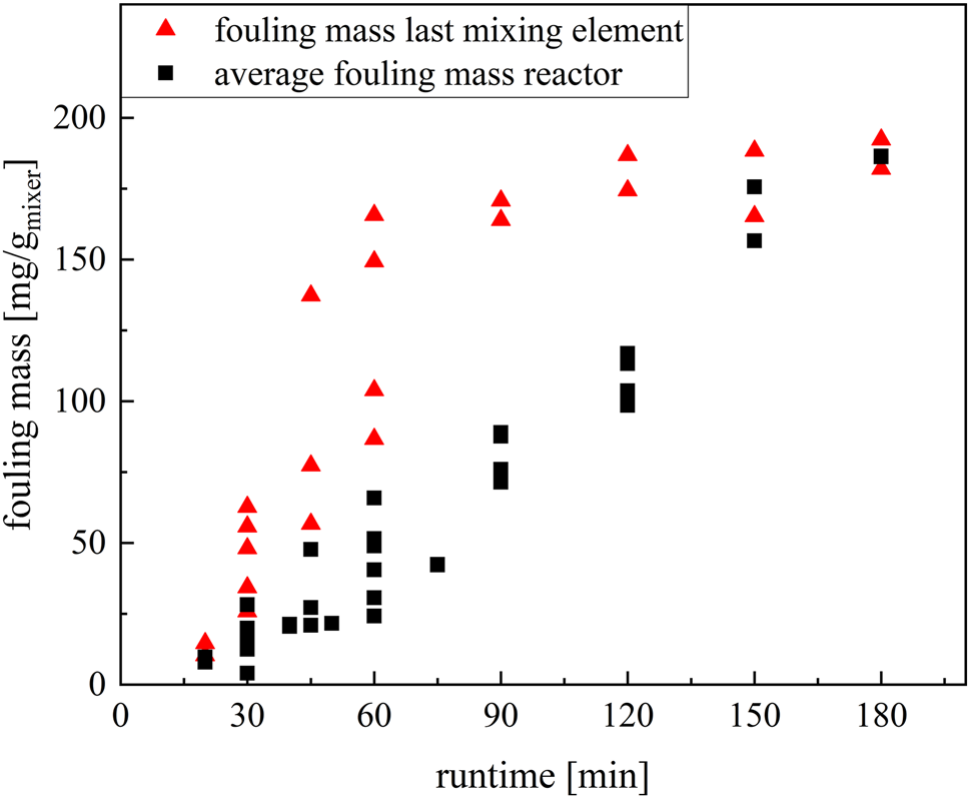
Plot of the mean fouling masses of the total reactor and the fouling masses of the last mixing element (ME7) over the runtime of the test at 20 °C.

The fouling largely reflects the expected conversion process in the reactor (Fig. 7). This agrees well with the literature^45^, which predicts a strong conversion dependence of the fouling. An exception to this is the first static mixer, in which the deposit formation is significantly increased. This is probably due to increased dead zones at the reactor inlet and deficits in the initial mixing^46^. Both favour an increased fouling, since the conversions are increased locally and the shear rates are also reduced by dead zones, so that sedimentation is favoured. After a minimum of fouling in static mixers two and three, the deposit formation then increases along the reactor and reaches its maximum near the reactor outlet. For technical implementation, the most fouling-intensive point is usually the most important, as this is most likely to lead to critical situations. Therefore, the time-dependent fouling of the last static mixer was compared with the average fouling mass for the whole reactor (Fig. 8).

The fouling formation at the reactor outlet is initially above the reactor mean. With increasing runtime, the deposit formation at the reactor outlet reaches a plateau and hardly increases any further. This course is attributed to that with increasing fouling, the flow velocity in the remaining channel volume is increased, so that further fouling is reduced due to the shear rates. Thus, a saturation is reached after which the fouling increase slightly. This saturation is reached at different times due to the different rates of fouling along the reactor, so that the mean value increases linearly for a long time, while saturation is already reached close to the reactor outlet.

### Conversion based online fouling sensing

The observation of the volume loss due to the fouling is a possible measurement method for fouling quantification. Here the volume loss was determined by conversion observation. Prerequisite for this is that the hydrodynamic residence time of the process corresponds approximately to the reaction time, so that a volume loss during the reaction directly influences the conversion. If the residence time is significantly higher than the reaction time, declines in conversion by reduced reaction volume will be detectable only at late fouling stages. Complete conversions would still be achieved even if the volume was reduced by significant fouling masses. In the set-up investigated here, the hydrodynamic residence time in the reactor was about 4 min, while the reaction time until complete thermal conversion of the investigated substance system in the batch reactor at 20 °C was about 3.5 min. In the batch process an increase in reactor temperature of about 20 °C was observed. In the continuous tubular reactor with improved cooling capacity compared to the batch reactor, the reaction time would therefore be somewhat longer and thus above the hydrodynamic residence time, so that the method is suitable here.

**Figure 9:**
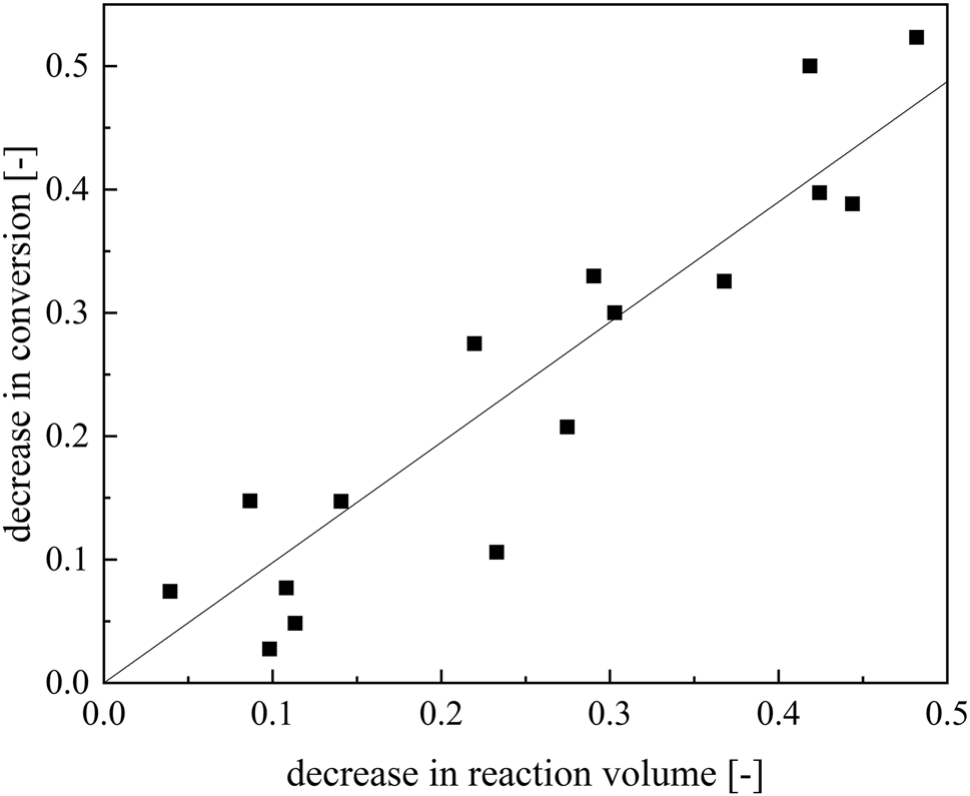
Plot of the decrease in conversion against the decrease in reaction volume for the experiments at 20 °C. The decrease in conversion is compared with the calculated decrease in reaction volume by gravimetrically determined reactor filling from the fouling masses in the reactor.

For this purpose, the reaction conversion at the reactor outlet was measured at regular intervals and the percentage conversion decrease over the runtime was determined. The conversion is directly proportional to the reaction volume at constant temperature and dosing currents, the decrease in conversion can be used to conclude the degree of filling in the reactor (Fig. 9).

The indirect determination of fouling masses by the decrease in conversion is in good agreement (R^2^ = 0.96) with the direct, gravimetric determination. To confirm that the regression requirements were met, the normal distribution of the residuals was analysed using Shapiro–Wilk. The test showed a significant normal distribution (p > 0.05). The determination of fouling masses by conversion analysis results in similar reactor fillings, the deviation of which lies within the measurement errors of the methods caused by conversion analytics or statistical deviations in fouling processes. Despite all possible deviations, the coverage of both methods is within 5%, so that the statistical deviation in the investigated fouling processes with 12% is significantly higher than the measurement errors. This method can be inline, online or atline depending on the method for determining the conversion. In this case, a real-time microwave gravimetric determination of conversions was used, so the method is between online and atline. The conversion could be easily accessed inline by calibrated Raman-spectroscopy as previously published^47^. Concluding, the real-time conversion analysis is a good strategy to quantify the amount of fouling fast. Although the conversion-based determination of the fouling masses is well practicable as a laboratory method, it is unsuitable for larger plants or routine operation due to the high effort involved.

### Inline fouling sensing using ultrasonic techniques

**Figure 10:**
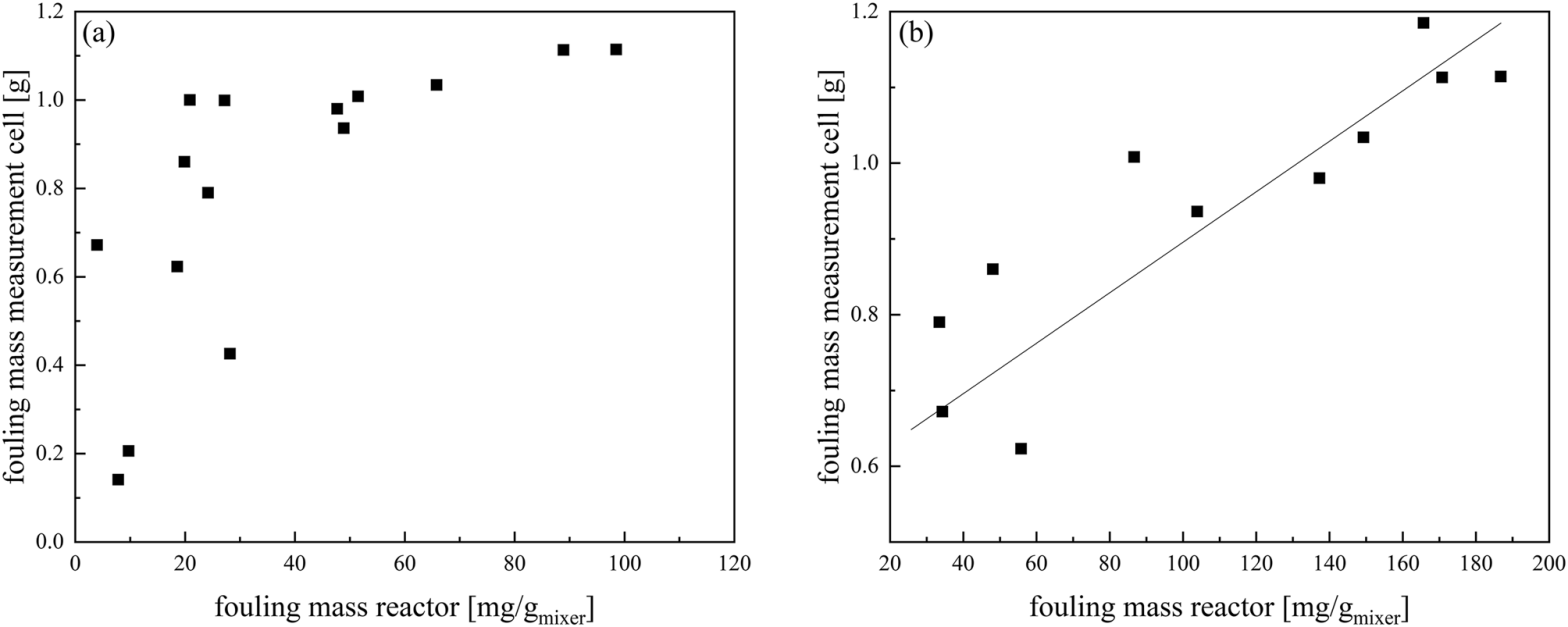
Plot of the fouling masses in the measurement cell against the average fouling masses of the reactor (**a**) and the fouling masses of the last static mixer (ME7) (**b**) for experiments at 20 °C.

The ultrasonic measuring technique can determine material characteristics which will be discussed as opportunity for fouling measurement. Since the reactor outlet was identified in the above considerations as the most fouling-intensive and therefore most critical point, the measuring cell was placed directly behind the reactor outlet. The fouling behaviour of the measuring cell was compared with the average fouling in the reactor (Fig. 10a) as well as the fouling at the reactor outlet (Fig. 10b) to validate that the measuring cell is representative of the fouling situation inside the reactor.

The average fouling masses of the reactor is not linear correlated with the fouling masses in the measurement cell. The measurement cell is especially in the beginning more sensitive to fouling deposition than the average reactor (Fig. 10a). In comparison the fouling at the last static mixer in the reactor agrees well with the gravimetrically determined fouling masses in the measuring cell (Fig. 10b). It is noticeable that for the whole runtime investigated up to 120 min there is a linear correlation between both fouling masses (R^2^ = 0.77, Shapiro–Wilk p > 0.05). It was chosen a regression of the type y = ax + b as there is a constant offset in fouling mass in the measurement cell caused by small dead zones, which is described by parameter b. With the measuring cell, the course of the reactor end can be mapped well, so that it is suitable for describing the fouling behaviour.

**Figure 11:**
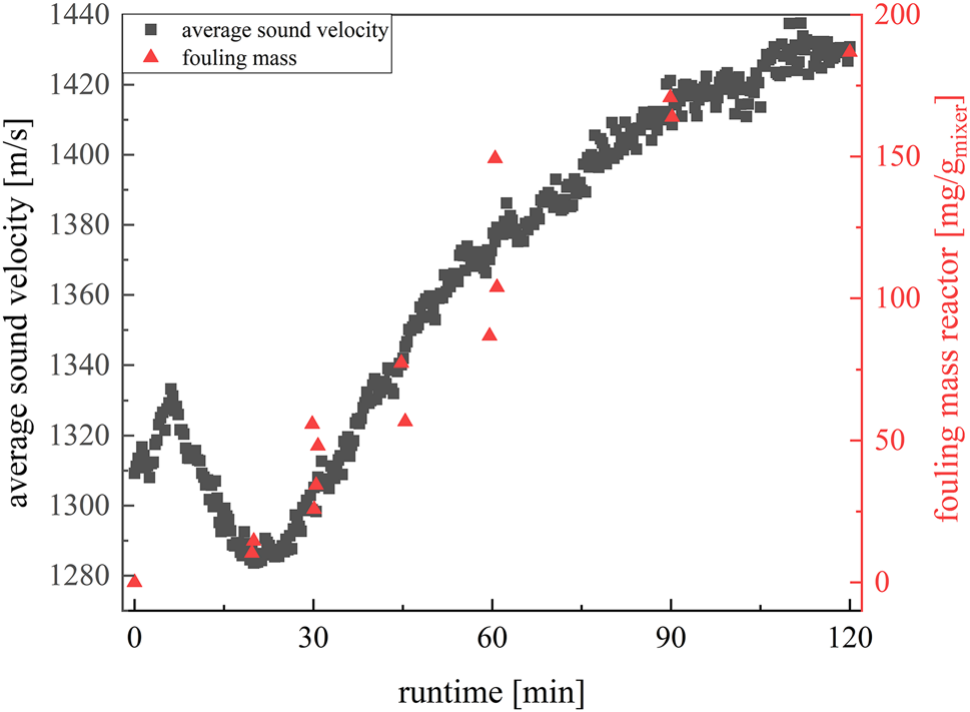
Plot of the fouling masses of the measuring cell and the average sound velocity against the runtime for one experiment at 20 °C.

Since the section with the most fouling is usually the most interesting, this method can represent this well. Further, it is still crucial to consider the directly accessible measurement variables of the ultrasonic measurement technology in which this trend can be depicted to obtain a useable technology. The average sound velocity (ASV) in the measuring cell is media dependent, so it is a promising approach for detecting fouling (Fig. 11).

**Figure 12:**
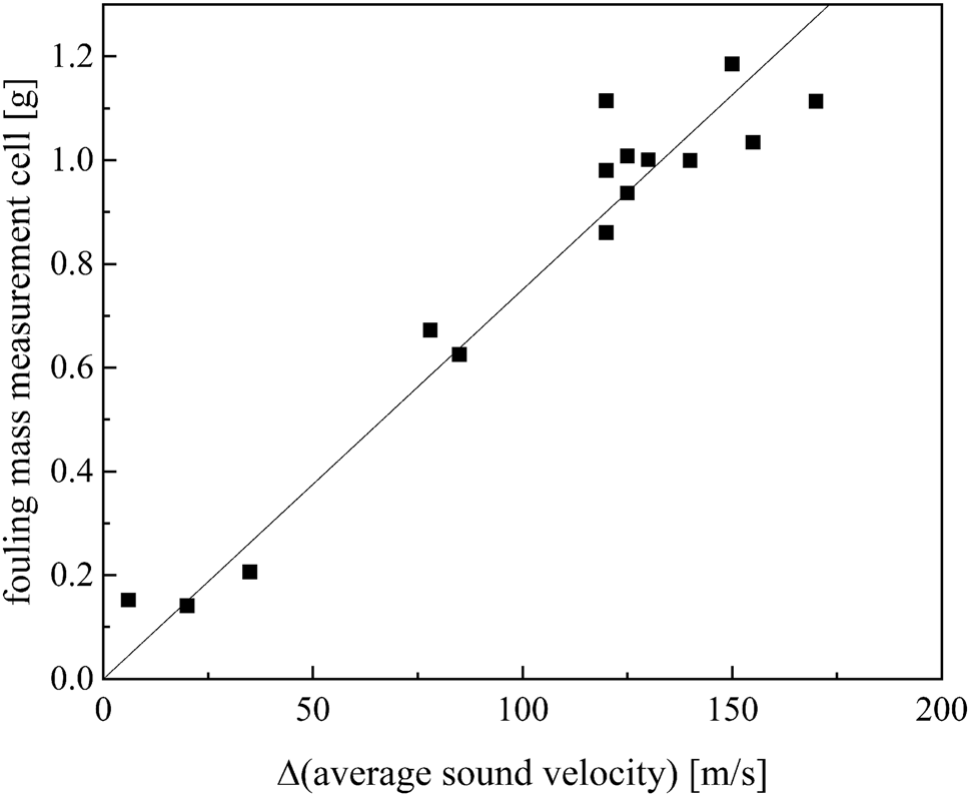
Plot of the fouling masses of the measuring cell against the change in mean sound velocity for experiments at 20 °C.

During the first 15 min of the experiment, the reaction has not reached stationarity so the average sound velocity is fluctuating (Fig. 11). These fluctuations of the ASV at the beginning of the measurement are caused by the low homogeneity of the pre-emulsion containing monomer droplets, micelles and water. About 7 min after starting the experiment first reaction can be detected in the measurement cell. This is expected as the hydrodynamic mean residence time for the reactor, connecting tubes and measurement cell is between 5 and 6 min. It can be recognized by the temperature increase due to the exothermic reaction but also visually by the resulting product. The curve of the average sound velocity clearly indicates the start of the reaction by the strong reduction of the average sound velocity, reaching a constant level with reaching the stationarity of the process after 15 min. In the following the fouling starts and the ASV is increasing as the solid fouling has a higher sound velocity than the emulsion. Time-resolved gravimetrically determined fouling masses correlate well with the change in average sound velocity indicating that the sound velocity is representing deposit formation. For validation the fouling mass in the measurement cell for 16 experiments are plotted against the difference in average sound velocity (Fig. 12).

The fouling mass and the average sound velocity correlates with R^2^ = 0.98 (Shapiro–Wilk p > 0.05). Concluding, the average sound velocity is suitable for inline monitoring of the fouling masses in the reactor.

**Figure 13:**
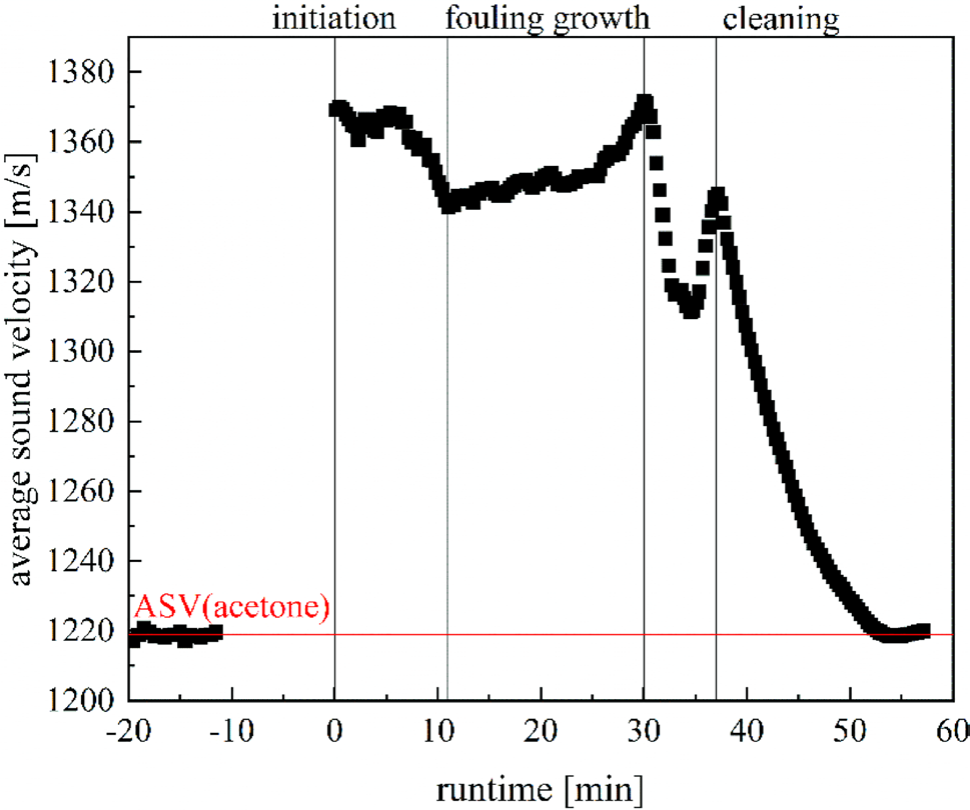
Plot of a complete deposit formation-cleaning cycle as proof of the conceptual suitability of the measurement technique at 20 °C.

As proof of the conceptual suitability of the measurement technique, a complete deposit formation cleaning cycle was followed using ultrasonic measurement technology. First, the fouling in the measuring cell and reactor was observed and then the cleaning of the reactor was followed using acetone (Fig. 13). Both the fouling mass before cleaning and the cleaning result were confirmed gravimetrically and optically.

The average sound velocity of acetone was measured as reference for cleaning. After that, the reaction was started and after 12 min the stationarity was reached and fouling growth can be observed. The runtime was set to 30 min and after that the experiment was ended and the measurement cell was rinsed with compressed air. In the next step acetone was used for cleaning and the decrease of the fouling layer can be observed starting 37 min after initiation. After 15 min of cleaning the average sound velocity reaches the level of pure acetone, so there should be no more fouling in the measurement cell which is confirmed visually. Only in the corners of the measuring cell outside the measuring path could small amounts of deposits still be detected optically, but these could not be measured because the measuring path was completely free of fouling. Further optimisation of the measuring cell is necessary here so that there are no undetectable zones, and the behaviour of the reactor can be described even more precisely.

## Summary

Two suitable sensor concepts were established, one monitoring fouling processes via conversion analysis and one using ultrasonic measurement techniques or fouling quantification. In the first case the loss in reaction volume caused by fouling was measured by the decrease in conversion during the reaction. In the second case the deposit formation was monitored by tracking the changes in average sound velocity in the measurement cell, which are substance-dependent and therefore caused by the formation of deposits. Both methods agree well with gravimetric references and reach deviations below 10%. Deposit formation is mainly caused by polymerisation fouling and for the average reactor value a linear fouling trend can be observed. This is caused by local differences in fouling behaviour as the conversion influences the fouling speed. The most intense fouling occurs at the reactor end causing a higher slope in fouling growth but reaching a saturation after 60 min as increased flow velocities reduce further fouling. Thus, the time-dependent fouling processes during continuous emulsion copolymerisation of vinyl acetate could be determined.

### Supplementary Information

Supplementary Information.

## Data Availability

All data generated or analysed during this study are included in this published article and its Supplementary Information files.
