# Supplementary material for: Ultrasonic and conversion-based inline fouling measurements for continuous emulsion copolymerisation of vinyl acetate in a tubular reactor
Source: Sci Rep. 2024 Feb 19;14:4077. doi: 10.1038/s41598-024-54321-4 (PMC10876618; doi:10.1038/s41598-024-54321-4)
Supplement: Supplementary file 1 — Supplementary Information. [file 41598_2024_54321_MOESM1_ESM.docx]

## Supplementary information for the publication “Ultrasonic and conversion-based inline fouling measurements for continuous emulsion copolymerisation of vinyl acetate in a tubular reactor”

## Author information

Sören Rust^1^, Marco Osenberg^2^, Thomas Musch^2^, Werner Pauer^1^

## Corresponding author

Werner Pauer, werner.pauer@chemie.uni-hamburg.de

## Affiliations

^1^ Institute for Technical and Macromolecular Chemistry, University of Hamburg, Bundesstraße 45, 20146 Hamburg, Germany

^2^ Ruhr-University Bochum, Chair of Electronic Circuits, Universitätsstraße 150, 44801 Bochum, Germany

Raw data Fig. 4+5

| experiment | emulsion | | | | | deposit | | | deposit reactor inlet |
| --- | --- | --- | --- | --- | --- | --- | --- | --- | --- |
| molecular weight | P-SR-455E | P-SR-456E | P-SR-457E | P-SR-458E | P-SR-459E | P-SR-456B Belag | P-SR-457B Belag | P-SR-458B Belag | P-SR-457A Anfang |
| 3.89E+02 | 0.00E+00 | 0.00E+00 | 0.00E+00 | 0.00E+00 | 0.00E+00 | 0.00E+00 | 0.00E+00 | 0.00E+00 | 0.00E+00 |
| 3.97E+02 | 0.00E+00 | 1.87E-03 | 0.00E+00 | 3.74E-06 | 0.00E+00 | 0.00E+00 | 0.00E+00 | 0.00E+00 | 8.26E-04 |
| 4.05E+02 | 3.77E-03 | 2.35E-02 | 0.00E+00 | 4.96E-04 | 0.00E+00 | 0.00E+00 | 0.00E+00 | 0.00E+00 | 1.12E-04 |
| 4.13E+02 | 1.02E-03 | 2.38E-02 | 9.93E-05 | 2.04E-02 | 0.00E+00 | 0.00E+00 | 0.00E+00 | 0.00E+00 | 1.44E-03 |
| 4.21E+02 | 4.54E-05 | 3.04E-02 | 1.62E-03 | 1.59E-02 | 0.00E+00 | 0.00E+00 | 0.00E+00 | 0.00E+00 | 3.90E-04 |
| 4.30E+02 | 1.22E-03 | 2.10E-02 | 0.00E+00 | 2.91E-02 | 0.00E+00 | 0.00E+00 | 0.00E+00 | 0.00E+00 | 6.20E-03 |
| 4.39E+02 | 7.03E-03 | 2.35E-02 | 2.54E-05 | 1.46E-02 | 0.00E+00 | 0.00E+00 | 0.00E+00 | 0.00E+00 | 1.24E-02 |
| 4.48E+02 | 4.10E-03 | 2.78E-02 | 4.93E-04 | 3.27E-02 | 0.00E+00 | 0.00E+00 | 0.00E+00 | 0.00E+00 | 1.45E-02 |
| 4.57E+02 | 7.88E-03 | 3.55E-02 | 2.62E-04 | 2.81E-02 | 0.00E+00 | 0.00E+00 | 0.00E+00 | 0.00E+00 | 2.98E-02 |
| 4.66E+02 | 5.61E-03 | 4.74E-02 | 1.50E-04 | 3.78E-02 | 2.41E-04 | 0.00E+00 | 0.00E+00 | 0.00E+00 | 3.38E-02 |
| 4.76E+02 | 2.65E-03 | 4.35E-02 | 3.60E-03 | 4.67E-02 | 4.82E-03 | 0.00E+00 | 0.00E+00 | 0.00E+00 | 3.39E-02 |
| 4.86E+02 | 8.52E-03 | 4.55E-02 | 5.97E-03 | 3.71E-02 | 5.39E-03 | 0.00E+00 | 0.00E+00 | 0.00E+00 | 3.51E-02 |
| 4.96E+02 | 8.08E-03 | 4.91E-02 | 1.55E-03 | 3.09E-02 | 1.34E-04 | 0.00E+00 | 0.00E+00 | 0.00E+00 | 4.78E-02 |
| 5.06E+02 | 7.22E-03 | 3.52E-02 | 4.31E-03 | 3.72E-02 | 3.49E-03 | 0.00E+00 | 0.00E+00 | 0.00E+00 | 5.40E-02 |
| 5.16E+02 | 6.76E-03 | 4.47E-02 | 9.75E-03 | 3.24E-02 | 1.22E-02 | 0.00E+00 | 0.00E+00 | 0.00E+00 | 6.40E-02 |
| 5.27E+02 | 9.08E-03 | 4.36E-02 | 4.32E-03 | 3.25E-02 | 4.91E-03 | 0.00E+00 | 0.00E+00 | 0.00E+00 | 5.89E-02 |
| 5.37E+02 | 4.38E-03 | 3.83E-02 | 1.23E-04 | 2.84E-02 | 6.56E-03 | 0.00E+00 | 0.00E+00 | 0.00E+00 | 5.83E-02 |
| 5.48E+02 | 1.75E-03 | 4.42E-02 | 4.66E-03 | 4.20E-02 | 2.05E-04 | 0.00E+00 | 0.00E+00 | 0.00E+00 | 5.74E-02 |
| 5.59E+02 | 6.08E-03 | 4.20E-02 | 0.00E+00 | 3.87E-02 | 1.52E-03 | 0.00E+00 | 0.00E+00 | 0.00E+00 | 6.19E-02 |
| 5.71E+02 | 4.11E-03 | 4.77E-02 | 5.60E-05 | 3.49E-02 | 0.00E+00 | 0.00E+00 | 0.00E+00 | 0.00E+00 | 7.46E-02 |
| 5.83E+02 | 2.99E-03 | 2.51E-02 | 2.99E-03 | 3.15E-02 | 0.00E+00 | 0.00E+00 | 0.00E+00 | 0.00E+00 | 5.99E-02 |
| 5.94E+02 | 3.94E-03 | 4.35E-02 | 3.48E-05 | 2.69E-02 | 0.00E+00 | 0.00E+00 | 0.00E+00 | 0.00E+00 | 6.74E-02 |
| 6.07E+02 | 4.39E-03 | 4.28E-02 | 2.55E-03 | 3.49E-02 | 8.21E-05 | 0.00E+00 | 0.00E+00 | 0.00E+00 | 8.93E-02 |
| 6.19E+02 | 5.96E-03 | 3.15E-02 | 2.72E-05 | 2.94E-02 | 4.23E-03 | 0.00E+00 | 0.00E+00 | 0.00E+00 | 8.38E-02 |
| 6.32E+02 | 3.52E-03 | 4.44E-02 | 3.15E-03 | 3.95E-02 | 0.00E+00 | 0.00E+00 | 0.00E+00 | 0.00E+00 | 6.97E-02 |
| 6.45E+02 | 1.89E-03 | 3.58E-02 | 4.15E-06 | 3.76E-02 | 6.29E-05 | 0.00E+00 | 0.00E+00 | 0.00E+00 | 8.76E-02 |
| 6.58E+02 | 2.20E-03 | 2.82E-02 | 1.14E-03 | 2.74E-02 | 5.42E-03 | 0.00E+00 | 0.00E+00 | 0.00E+00 | 8.31E-02 |
| 6.71E+02 | 2.31E-03 | 4.00E-02 | 1.45E-06 | 3.86E-02 | 1.73E-02 | 0.00E+00 | 0.00E+00 | 0.00E+00 | 8.84E-02 |
| 6.85E+02 | 3.76E-04 | 5.27E-02 | 3.55E-03 | 3.47E-02 | 1.96E-06 | 0.00E+00 | 0.00E+00 | 0.00E+00 | 9.31E-02 |
| 6.99E+02 | 2.66E-03 | 5.21E-02 | 6.99E-03 | 2.78E-02 | 4.21E-04 | 0.00E+00 | 0.00E+00 | 0.00E+00 | 8.36E-02 |
| 7.13E+02 | 5.98E-03 | 4.08E-02 | 6.07E-05 | 4.61E-02 | 7.03E-08 | 0.00E+00 | 0.00E+00 | 0.00E+00 | 7.31E-02 |
| 7.28E+02 | 6.05E-03 | 3.67E-02 | 4.66E-03 | 3.72E-02 | 1.92E-05 | 0.00E+00 | 0.00E+00 | 0.00E+00 | 8.26E-02 |
| 7.43E+02 | 8.99E-03 | 3.63E-02 | 1.41E-02 | 3.44E-02 | 9.59E-08 | 0.00E+00 | 0.00E+00 | 0.00E+00 | 7.79E-02 |
| 7.58E+02 | 9.41E-03 | 3.66E-02 | 5.19E-03 | 3.03E-02 | 1.50E-02 | 0.00E+00 | 0.00E+00 | 0.00E+00 | 7.67E-02 |
| 7.73E+02 | 8.85E-03 | 4.84E-02 | 6.36E-03 | 3.97E-02 | 1.28E-02 | 0.00E+00 | 0.00E+00 | 0.00E+00 | 7.79E-02 |
| 7.89E+02 | 1.86E-02 | 5.52E-02 | 8.47E-03 | 4.37E-02 | 1.56E-04 | 0.00E+00 | 0.00E+00 | 0.00E+00 | 8.39E-02 |
| 8.05E+02 | 1.55E-02 | 5.10E-02 | 9.32E-03 | 4.92E-02 | 1.85E-02 | 0.00E+00 | 0.00E+00 | 0.00E+00 | 1.03E-01 |
| 8.22E+02 | 1.33E-02 | 4.40E-02 | 8.60E-03 | 4.15E-02 | 2.23E-02 | 0.00E+00 | 0.00E+00 | 0.00E+00 | 7.89E-02 |
| 8.38E+02 | 8.88E-03 | 3.49E-02 | 1.14E-02 | 4.41E-02 | 1.69E-03 | 0.00E+00 | 0.00E+00 | 0.00E+00 | 8.22E-02 |
| 8.56E+02 | 1.56E-02 | 4.10E-02 | 1.74E-02 | 5.37E-02 | 2.67E-02 | 0.00E+00 | 0.00E+00 | 0.00E+00 | 8.87E-02 |
| 8.73E+02 | 1.24E-02 | 6.32E-02 | 8.98E-03 | 4.25E-02 | 1.51E-02 | 0.00E+00 | 0.00E+00 | 0.00E+00 | 9.81E-02 |
| 8.91E+02 | 1.29E-02 | 4.23E-02 | 1.38E-02 | 4.86E-02 | 1.30E-02 | 0.00E+00 | 0.00E+00 | 0.00E+00 | 8.96E-02 |
| 9.09E+02 | 2.14E-02 | 5.04E-02 | 2.28E-02 | 4.63E-02 | 3.77E-04 | 0.00E+00 | 0.00E+00 | 0.00E+00 | 8.19E-02 |
| 9.28E+02 | 2.06E-02 | 6.98E-02 | 1.57E-02 | 5.86E-02 | 2.88E-02 | 0.00E+00 | 0.00E+00 | 0.00E+00 | 9.51E-02 |
| 9.47E+02 | 2.13E-02 | 6.33E-02 | 1.64E-02 | 5.64E-02 | 2.33E-02 | 0.00E+00 | 0.00E+00 | 0.00E+00 | 9.72E-02 |
| 9.66E+02 | 1.52E-02 | 4.98E-02 | 2.35E-02 | 4.65E-02 | 3.06E-02 | 0.00E+00 | 0.00E+00 | 0.00E+00 | 8.58E-02 |
| 9.86E+02 | 2.69E-02 | 6.97E-02 | 2.13E-02 | 5.15E-02 | 2.40E-02 | 0.00E+00 | 0.00E+00 | 0.00E+00 | 9.06E-02 |
| 1.01E+03 | 2.23E-02 | 6.50E-02 | 2.27E-02 | 5.88E-02 | 4.72E-02 | 0.00E+00 | 0.00E+00 | 0.00E+00 | 9.60E-02 |
| 1.03E+03 | 2.43E-02 | 7.15E-02 | 2.96E-02 | 6.26E-02 | 3.01E-02 | 0.00E+00 | 0.00E+00 | 0.00E+00 | 9.22E-02 |
| 1.05E+03 | 3.13E-02 | 7.03E-02 | 3.00E-02 | 6.07E-02 | 3.77E-02 | 0.00E+00 | 0.00E+00 | 0.00E+00 | 8.47E-02 |
| 1.07E+03 | 2.50E-02 | 3.84E-02 | 2.14E-02 | 5.88E-02 | 4.25E-02 | 0.00E+00 | 0.00E+00 | 0.00E+00 | 9.41E-02 |
| 1.09E+03 | 2.84E-02 | 5.83E-02 | 2.65E-02 | 6.46E-02 | 4.66E-02 | 0.00E+00 | 0.00E+00 | 0.00E+00 | 9.91E-02 |
| 1.11E+03 | 3.47E-02 | 6.67E-02 | 3.18E-02 | 6.22E-02 | 3.85E-02 | 0.00E+00 | 0.00E+00 | 0.00E+00 | 1.00E-01 |
| 1.14E+03 | 3.69E-02 | 5.13E-02 | 3.00E-02 | 7.40E-02 | 3.18E-02 | 0.00E+00 | 0.00E+00 | 0.00E+00 | 9.15E-02 |
| 1.16E+03 | 3.19E-02 | 6.72E-02 | 2.99E-02 | 6.98E-02 | 3.08E-02 | 0.00E+00 | 0.00E+00 | 0.00E+00 | 7.98E-02 |
| 1.18E+03 | 4.08E-02 | 7.56E-02 | 3.19E-02 | 6.59E-02 | 3.40E-02 | 0.00E+00 | 0.00E+00 | 0.00E+00 | 9.27E-02 |
| 1.21E+03 | 4.40E-02 | 7.14E-02 | 3.52E-02 | 6.52E-02 | 3.71E-02 | 0.00E+00 | 0.00E+00 | 0.00E+00 | 1.08E-01 |
| 1.23E+03 | 3.92E-02 | 6.98E-02 | 3.72E-02 | 7.35E-02 | 4.51E-02 | 0.00E+00 | 0.00E+00 | 0.00E+00 | 1.01E-01 |
| 1.26E+03 | 3.76E-02 | 6.10E-02 | 3.35E-02 | 7.37E-02 | 5.29E-02 | 0.00E+00 | 0.00E+00 | 0.00E+00 | 1.07E-01 |
| 1.28E+03 | 3.62E-02 | 6.85E-02 | 4.04E-02 | 6.96E-02 | 6.17E-02 | 0.00E+00 | 0.00E+00 | 0.00E+00 | 1.07E-01 |
| 1.31E+03 | 4.55E-02 | 8.54E-02 | 3.72E-02 | 7.45E-02 | 6.22E-02 | 0.00E+00 | 0.00E+00 | 0.00E+00 | 1.08E-01 |
| 1.34E+03 | 4.66E-02 | 7.77E-02 | 4.08E-02 | 6.99E-02 | 5.85E-02 | 0.00E+00 | 0.00E+00 | 0.00E+00 | 1.17E-01 |
| 1.36E+03 | 4.83E-02 | 8.36E-02 | 3.92E-02 | 6.80E-02 | 6.59E-02 | 0.00E+00 | 0.00E+00 | 0.00E+00 | 1.03E-01 |
| 1.39E+03 | 5.19E-02 | 8.68E-02 | 4.77E-02 | 7.47E-02 | 6.05E-02 | 0.00E+00 | 0.00E+00 | 0.00E+00 | 1.02E-01 |
| 1.42E+03 | 5.54E-02 | 8.25E-02 | 4.67E-02 | 8.28E-02 | 8.09E-02 | 0.00E+00 | 0.00E+00 | 0.00E+00 | 9.93E-02 |
| 1.45E+03 | 5.32E-02 | 7.52E-02 | 5.17E-02 | 7.27E-02 | 7.08E-02 | 0.00E+00 | 0.00E+00 | 0.00E+00 | 9.42E-02 |
| 1.48E+03 | 5.44E-02 | 8.87E-02 | 5.40E-02 | 8.31E-02 | 6.90E-02 | 0.00E+00 | 0.00E+00 | 0.00E+00 | 1.08E-01 |
| 1.51E+03 | 5.69E-02 | 9.72E-02 | 4.78E-02 | 8.45E-02 | 6.63E-02 | 0.00E+00 | 0.00E+00 | 0.00E+00 | 1.10E-01 |
| 1.54E+03 | 5.97E-02 | 7.90E-02 | 5.04E-02 | 9.00E-02 | 7.47E-02 | 0.00E+00 | 0.00E+00 | 0.00E+00 | 1.07E-01 |
| 1.57E+03 | 5.37E-02 | 1.03E-01 | 4.94E-02 | 8.19E-02 | 7.73E-02 | 0.00E+00 | 0.00E+00 | 0.00E+00 | 1.22E-01 |
| 1.60E+03 | 6.38E-02 | 1.04E-01 | 5.28E-02 | 7.54E-02 | 8.40E-02 | 0.00E+00 | 0.00E+00 | 0.00E+00 | 1.14E-01 |
| 1.63E+03 | 6.40E-02 | 1.08E-01 | 6.38E-02 | 8.50E-02 | 8.26E-02 | 0.00E+00 | 0.00E+00 | 0.00E+00 | 1.12E-01 |
| 1.67E+03 | 5.89E-02 | 9.81E-02 | 6.21E-02 | 9.21E-02 | 9.04E-02 | 0.00E+00 | 0.00E+00 | 0.00E+00 | 1.09E-01 |
| 1.70E+03 | 6.22E-02 | 1.06E-01 | 5.73E-02 | 9.62E-02 | 7.15E-02 | 0.00E+00 | 0.00E+00 | 0.00E+00 | 1.16E-01 |
| 1.74E+03 | 6.46E-02 | 1.12E-01 | 5.82E-02 | 8.97E-02 | 8.29E-02 | 0.00E+00 | 0.00E+00 | 0.00E+00 | 1.30E-01 |
| 1.77E+03 | 6.47E-02 | 1.13E-01 | 6.35E-02 | 9.13E-02 | 6.76E-02 | 0.00E+00 | 0.00E+00 | 0.00E+00 | 1.29E-01 |
| 1.81E+03 | 7.22E-02 | 1.15E-01 | 6.30E-02 | 1.02E-01 | 8.44E-02 | 0.00E+00 | 0.00E+00 | 0.00E+00 | 1.22E-01 |
| 1.85E+03 | 7.61E-02 | 9.84E-02 | 6.40E-02 | 9.43E-02 | 9.56E-02 | 0.00E+00 | 0.00E+00 | 0.00E+00 | 1.26E-01 |
| 1.88E+03 | 6.93E-02 | 1.12E-01 | 6.03E-02 | 1.00E-01 | 9.99E-02 | 0.00E+00 | 0.00E+00 | 0.00E+00 | 1.16E-01 |
| 1.92E+03 | 7.36E-02 | 1.07E-01 | 6.37E-02 | 1.11E-01 | 9.77E-02 | 0.00E+00 | 0.00E+00 | 0.00E+00 | 1.14E-01 |
| 1.96E+03 | 8.11E-02 | 1.05E-01 | 6.75E-02 | 1.10E-01 | 1.05E-01 | 0.00E+00 | 0.00E+00 | 0.00E+00 | 1.22E-01 |
| 2.00E+03 | 8.11E-02 | 1.04E-01 | 7.57E-02 | 1.08E-01 | 1.07E-01 | 0.00E+00 | 0.00E+00 | 0.00E+00 | 1.26E-01 |
| 2.04E+03 | 7.21E-02 | 1.11E-01 | 7.52E-02 | 1.06E-01 | 1.16E-01 | 0.00E+00 | 0.00E+00 | 0.00E+00 | 1.26E-01 |
| 2.08E+03 | 8.24E-02 | 9.28E-02 | 7.08E-02 | 1.08E-01 | 1.09E-01 | 0.00E+00 | 0.00E+00 | 0.00E+00 | 1.17E-01 |
| 2.13E+03 | 8.62E-02 | 1.15E-01 | 7.47E-02 | 1.10E-01 | 1.17E-01 | 0.00E+00 | 0.00E+00 | 0.00E+00 | 1.28E-01 |
| 2.17E+03 | 7.64E-02 | 1.17E-01 | 7.64E-02 | 1.08E-01 | 1.06E-01 | 0.00E+00 | 0.00E+00 | 0.00E+00 | 1.36E-01 |
| 2.21E+03 | 8.63E-02 | 1.32E-01 | 8.02E-02 | 1.12E-01 | 1.08E-01 | 0.00E+00 | 0.00E+00 | 0.00E+00 | 1.33E-01 |
| 2.26E+03 | 8.58E-02 | 1.27E-01 | 8.39E-02 | 1.11E-01 | 1.10E-01 | 0.00E+00 | 0.00E+00 | 0.00E+00 | 1.36E-01 |
| 2.31E+03 | 8.45E-02 | 1.30E-01 | 8.81E-02 | 1.15E-01 | 1.13E-01 | 0.00E+00 | 0.00E+00 | 0.00E+00 | 1.30E-01 |
| 2.35E+03 | 8.05E-02 | 1.32E-01 | 8.25E-02 | 1.17E-01 | 1.10E-01 | 0.00E+00 | 0.00E+00 | 0.00E+00 | 1.40E-01 |
| 2.40E+03 | 8.78E-02 | 1.29E-01 | 8.70E-02 | 1.15E-01 | 1.27E-01 | 0.00E+00 | 0.00E+00 | 0.00E+00 | 1.38E-01 |
| 2.45E+03 | 8.91E-02 | 1.36E-01 | 9.09E-02 | 1.32E-01 | 1.32E-01 | 0.00E+00 | 0.00E+00 | 0.00E+00 | 1.15E-01 |
| 2.50E+03 | 9.35E-02 | 1.28E-01 | 8.82E-02 | 1.27E-01 | 1.32E-01 | 0.00E+00 | 0.00E+00 | 0.00E+00 | 1.32E-01 |
| 2.55E+03 | 9.71E-02 | 1.31E-01 | 9.57E-02 | 1.29E-01 | 1.37E-01 | 0.00E+00 | 0.00E+00 | 0.00E+00 | 1.32E-01 |
| 2.60E+03 | 9.62E-02 | 1.43E-01 | 9.42E-02 | 1.25E-01 | 1.39E-01 | 0.00E+00 | 0.00E+00 | 0.00E+00 | 1.51E-01 |
| 2.66E+03 | 9.71E-02 | 1.39E-01 | 9.54E-02 | 1.26E-01 | 1.29E-01 | 0.00E+00 | 0.00E+00 | 0.00E+00 | 1.45E-01 |
| 2.71E+03 | 1.03E-01 | 1.34E-01 | 1.03E-01 | 1.36E-01 | 1.49E-01 | 0.00E+00 | 0.00E+00 | 0.00E+00 | 1.31E-01 |
| 2.77E+03 | 9.72E-02 | 1.47E-01 | 9.69E-02 | 1.36E-01 | 1.46E-01 | 0.00E+00 | 0.00E+00 | 0.00E+00 | 1.46E-01 |
| 2.82E+03 | 1.12E-01 | 1.36E-01 | 9.60E-02 | 1.41E-01 | 1.44E-01 | 0.00E+00 | 0.00E+00 | 0.00E+00 | 1.43E-01 |
| 2.88E+03 | 1.12E-01 | 1.33E-01 | 9.68E-02 | 1.45E-01 | 1.59E-01 | 0.00E+00 | 0.00E+00 | 0.00E+00 | 1.49E-01 |
| 2.94E+03 | 1.12E-01 | 1.48E-01 | 1.11E-01 | 1.42E-01 | 1.47E-01 | 0.00E+00 | 0.00E+00 | 0.00E+00 | 1.65E-01 |
| 3.00E+03 | 1.17E-01 | 1.54E-01 | 1.10E-01 | 1.49E-01 | 1.47E-01 | 0.00E+00 | 0.00E+00 | 0.00E+00 | 1.55E-01 |
| 3.06E+03 | 1.12E-01 | 1.60E-01 | 1.10E-01 | 1.47E-01 | 1.58E-01 | 0.00E+00 | 0.00E+00 | 0.00E+00 | 1.73E-01 |
| 3.12E+03 | 1.14E-01 | 1.47E-01 | 1.16E-01 | 1.43E-01 | 1.71E-01 | 0.00E+00 | 0.00E+00 | 0.00E+00 | 1.61E-01 |
| 3.19E+03 | 1.20E-01 | 1.53E-01 | 1.20E-01 | 1.43E-01 | 1.64E-01 | 0.00E+00 | 0.00E+00 | 0.00E+00 | 1.63E-01 |
| 3.25E+03 | 1.17E-01 | 1.52E-01 | 1.24E-01 | 1.55E-01 | 1.59E-01 | 0.00E+00 | 0.00E+00 | 0.00E+00 | 1.61E-01 |
| 3.32E+03 | 1.27E-01 | 1.54E-01 | 1.28E-01 | 1.53E-01 | 1.74E-01 | 0.00E+00 | 0.00E+00 | 0.00E+00 | 1.69E-01 |
| 3.39E+03 | 1.23E-01 | 1.76E-01 | 1.25E-01 | 1.51E-01 | 1.70E-01 | 0.00E+00 | 0.00E+00 | 0.00E+00 | 1.71E-01 |
| 3.46E+03 | 1.22E-01 | 1.67E-01 | 1.27E-01 | 1.59E-01 | 1.81E-01 | 0.00E+00 | 0.00E+00 | 0.00E+00 | 1.75E-01 |
| 3.53E+03 | 1.31E-01 | 1.62E-01 | 1.33E-01 | 1.62E-01 | 1.84E-01 | 0.00E+00 | 0.00E+00 | 0.00E+00 | 1.78E-01 |
| 3.60E+03 | 1.30E-01 | 1.75E-01 | 1.35E-01 | 1.64E-01 | 1.83E-01 | 0.00E+00 | 0.00E+00 | 0.00E+00 | 1.83E-01 |
| 3.67E+03 | 1.39E-01 | 1.65E-01 | 1.29E-01 | 1.64E-01 | 1.88E-01 | 0.00E+00 | 0.00E+00 | 0.00E+00 | 1.75E-01 |
| 3.75E+03 | 1.43E-01 | 1.74E-01 | 1.39E-01 | 1.69E-01 | 1.75E-01 | 0.00E+00 | 0.00E+00 | 0.00E+00 | 1.75E-01 |
| 3.82E+03 | 1.44E-01 | 1.84E-01 | 1.41E-01 | 1.70E-01 | 1.92E-01 | 0.00E+00 | 0.00E+00 | 0.00E+00 | 1.84E-01 |
| 3.90E+03 | 1.47E-01 | 1.82E-01 | 1.41E-01 | 1.80E-01 | 1.95E-01 | 0.00E+00 | 0.00E+00 | 0.00E+00 | 1.78E-01 |
| 3.98E+03 | 1.47E-01 | 1.67E-01 | 1.41E-01 | 1.88E-01 | 1.90E-01 | 0.00E+00 | 0.00E+00 | 0.00E+00 | 1.95E-01 |
| 4.06E+03 | 1.52E-01 | 1.85E-01 | 1.45E-01 | 1.88E-01 | 1.92E-01 | 0.00E+00 | 0.00E+00 | 0.00E+00 | 1.84E-01 |
| 4.15E+03 | 1.57E-01 | 1.92E-01 | 1.48E-01 | 1.84E-01 | 2.05E-01 | 0.00E+00 | 0.00E+00 | 0.00E+00 | 1.89E-01 |
| 4.23E+03 | 1.61E-01 | 1.76E-01 | 1.50E-01 | 1.85E-01 | 1.99E-01 | 0.00E+00 | 0.00E+00 | 0.00E+00 | 1.94E-01 |
| 4.32E+03 | 1.56E-01 | 1.87E-01 | 1.47E-01 | 1.84E-01 | 2.19E-01 | 0.00E+00 | 0.00E+00 | 0.00E+00 | 1.86E-01 |
| 4.40E+03 | 1.61E-01 | 2.00E-01 | 1.51E-01 | 1.90E-01 | 2.30E-01 | 0.00E+00 | 0.00E+00 | 0.00E+00 | 1.88E-01 |
| 4.50E+03 | 1.62E-01 | 2.01E-01 | 1.61E-01 | 1.97E-01 | 2.29E-01 | 0.00E+00 | 0.00E+00 | 0.00E+00 | 1.88E-01 |
| 4.59E+03 | 1.65E-01 | 1.95E-01 | 1.58E-01 | 2.00E-01 | 2.40E-01 | 0.00E+00 | 0.00E+00 | 0.00E+00 | 1.78E-01 |
| 4.68E+03 | 1.74E-01 | 2.08E-01 | 1.63E-01 | 1.99E-01 | 2.33E-01 | 0.00E+00 | 0.00E+00 | 6.49E-03 | 1.84E-01 |
| 4.78E+03 | 1.73E-01 | 2.09E-01 | 1.71E-01 | 1.95E-01 | 2.41E-01 | 0.00E+00 | 0.00E+00 | 5.16E-03 | 1.95E-01 |
| 4.87E+03 | 1.71E-01 | 2.23E-01 | 1.72E-01 | 2.02E-01 | 2.44E-01 | 0.00E+00 | 0.00E+00 | 0.00E+00 | 1.92E-01 |
| 4.97E+03 | 1.83E-01 | 2.23E-01 | 1.75E-01 | 2.05E-01 | 2.57E-01 | 0.00E+00 | 0.00E+00 | 0.00E+00 | 1.90E-01 |
| 5.08E+03 | 1.81E-01 | 2.08E-01 | 1.78E-01 | 2.11E-01 | 2.46E-01 | 0.00E+00 | 0.00E+00 | 0.00E+00 | 1.94E-01 |
| 5.18E+03 | 1.85E-01 | 2.16E-01 | 1.85E-01 | 2.09E-01 | 2.47E-01 | 0.00E+00 | 0.00E+00 | 0.00E+00 | 1.93E-01 |
| 5.28E+03 | 1.92E-01 | 2.09E-01 | 1.86E-01 | 2.14E-01 | 2.49E-01 | 0.00E+00 | 0.00E+00 | 1.21E-03 | 2.07E-01 |
| 5.39E+03 | 1.90E-01 | 2.26E-01 | 1.84E-01 | 2.29E-01 | 2.55E-01 | 0.00E+00 | 0.00E+00 | 3.54E-03 | 2.13E-01 |
| 5.50E+03 | 1.93E-01 | 2.26E-01 | 1.85E-01 | 2.26E-01 | 2.74E-01 | 0.00E+00 | 0.00E+00 | 2.18E-03 | 1.99E-01 |
| 5.62E+03 | 1.90E-01 | 2.10E-01 | 1.95E-01 | 2.22E-01 | 2.79E-01 | 0.00E+00 | 0.00E+00 | 8.52E-03 | 2.08E-01 |
| 5.73E+03 | 1.98E-01 | 2.30E-01 | 1.98E-01 | 2.35E-01 | 2.65E-01 | 3.80E-03 | 0.00E+00 | 1.14E-02 | 2.09E-01 |
| 5.85E+03 | 2.00E-01 | 2.33E-01 | 1.93E-01 | 2.32E-01 | 2.59E-01 | 2.62E-03 | 0.00E+00 | 5.06E-03 | 2.15E-01 |
| 5.97E+03 | 2.05E-01 | 2.46E-01 | 2.00E-01 | 2.33E-01 | 2.75E-01 | 0.00E+00 | 0.00E+00 | 4.30E-03 | 2.23E-01 |
| 6.09E+03 | 2.11E-01 | 2.45E-01 | 2.00E-01 | 2.42E-01 | 2.86E-01 | 0.00E+00 | 0.00E+00 | 1.52E-02 | 2.12E-01 |
| 6.21E+03 | 2.15E-01 | 2.33E-01 | 2.07E-01 | 2.44E-01 | 2.93E-01 | 0.00E+00 | 0.00E+00 | 2.16E-02 | 2.05E-01 |
| 6.34E+03 | 2.23E-01 | 2.39E-01 | 2.11E-01 | 2.49E-01 | 2.98E-01 | 2.55E-02 | 0.00E+00 | 1.67E-02 | 2.20E-01 |
| 6.47E+03 | 2.20E-01 | 2.49E-01 | 2.14E-01 | 2.49E-01 | 3.06E-01 | 2.98E-02 | 0.00E+00 | 1.15E-02 | 2.15E-01 |
| 6.60E+03 | 2.24E-01 | 2.53E-01 | 2.20E-01 | 2.46E-01 | 2.92E-01 | 2.43E-02 | 0.00E+00 | 1.24E-02 | 2.30E-01 |
| 6.74E+03 | 2.30E-01 | 2.50E-01 | 2.16E-01 | 2.56E-01 | 3.01E-01 | 2.98E-02 | 0.00E+00 | 1.94E-02 | 2.27E-01 |
| 6.87E+03 | 2.27E-01 | 2.56E-01 | 2.17E-01 | 2.61E-01 | 3.04E-01 | 3.26E-02 | 0.00E+00 | 2.47E-02 | 2.22E-01 |
| 7.01E+03 | 2.34E-01 | 2.61E-01 | 2.26E-01 | 2.57E-01 | 3.26E-01 | 1.98E-02 | 0.00E+00 | 1.54E-02 | 2.25E-01 |
| 7.16E+03 | 2.37E-01 | 2.64E-01 | 2.34E-01 | 2.57E-01 | 3.38E-01 | 2.32E-02 | 0.00E+00 | 2.06E-02 | 2.40E-01 |
| 7.30E+03 | 2.43E-01 | 2.65E-01 | 2.25E-01 | 2.61E-01 | 3.30E-01 | 4.05E-02 | 0.00E+00 | 3.22E-02 | 2.51E-01 |
| 7.45E+03 | 2.44E-01 | 2.71E-01 | 2.32E-01 | 2.70E-01 | 3.29E-01 | 4.46E-02 | 0.00E+00 | 2.80E-02 | 2.48E-01 |
| 7.61E+03 | 2.47E-01 | 2.68E-01 | 2.41E-01 | 2.71E-01 | 3.36E-01 | 5.58E-02 | 0.00E+00 | 3.47E-02 | 2.49E-01 |
| 7.76E+03 | 2.48E-01 | 2.85E-01 | 2.45E-01 | 2.77E-01 | 3.38E-01 | 5.40E-02 | 0.00E+00 | 3.67E-02 | 2.49E-01 |
| 7.92E+03 | 2.58E-01 | 2.81E-01 | 2.49E-01 | 2.81E-01 | 3.40E-01 | 4.22E-02 | 0.00E+00 | 3.62E-02 | 2.52E-01 |
| 8.08E+03 | 2.62E-01 | 2.82E-01 | 2.51E-01 | 2.81E-01 | 3.56E-01 | 7.34E-02 | 0.00E+00 | 4.22E-02 | 2.45E-01 |
| 8.25E+03 | 2.64E-01 | 2.83E-01 | 2.54E-01 | 2.90E-01 | 3.56E-01 | 6.87E-02 | 0.00E+00 | 4.92E-02 | 2.44E-01 |
| 8.42E+03 | 2.71E-01 | 2.84E-01 | 2.55E-01 | 2.97E-01 | 3.65E-01 | 6.31E-02 | 0.00E+00 | 4.21E-02 | 2.55E-01 |
| 8.59E+03 | 2.74E-01 | 2.82E-01 | 2.64E-01 | 3.00E-01 | 3.70E-01 | 6.65E-02 | 0.00E+00 | 3.28E-02 | 2.71E-01 |
| 8.76E+03 | 2.74E-01 | 2.91E-01 | 2.65E-01 | 3.08E-01 | 3.74E-01 | 6.59E-02 | 0.00E+00 | 3.77E-02 | 2.66E-01 |
| 8.94E+03 | 2.73E-01 | 3.10E-01 | 2.71E-01 | 3.17E-01 | 3.68E-01 | 7.83E-02 | 0.00E+00 | 4.82E-02 | 2.64E-01 |
| 9.13E+03 | 2.84E-01 | 3.06E-01 | 2.78E-01 | 3.12E-01 | 3.87E-01 | 8.23E-02 | 0.00E+00 | 5.59E-02 | 2.76E-01 |
| 9.31E+03 | 2.91E-01 | 3.11E-01 | 2.80E-01 | 3.14E-01 | 3.84E-01 | 9.82E-02 | 0.00E+00 | 5.73E-02 | 2.73E-01 |
| 9.50E+03 | 2.90E-01 | 3.11E-01 | 2.85E-01 | 3.20E-01 | 3.89E-01 | 9.39E-02 | 0.00E+00 | 5.49E-02 | 2.77E-01 |
| 9.70E+03 | 3.00E-01 | 3.20E-01 | 2.88E-01 | 3.25E-01 | 3.91E-01 | 8.72E-02 | 0.00E+00 | 5.75E-02 | 2.65E-01 |
| 9.89E+03 | 3.02E-01 | 3.15E-01 | 2.87E-01 | 3.27E-01 | 3.96E-01 | 1.00E-01 | 0.00E+00 | 7.31E-02 | 2.78E-01 |
| 1.01E+04 | 3.08E-01 | 3.27E-01 | 2.95E-01 | 3.31E-01 | 4.07E-01 | 9.81E-02 | 0.00E+00 | 6.79E-02 | 2.83E-01 |
| 1.03E+04 | 3.12E-01 | 3.18E-01 | 3.04E-01 | 3.32E-01 | 4.15E-01 | 9.75E-02 | 0.00E+00 | 7.04E-02 | 2.86E-01 |
| 1.05E+04 | 3.07E-01 | 3.28E-01 | 3.05E-01 | 3.42E-01 | 4.13E-01 | 1.12E-01 | 0.00E+00 | 7.01E-02 | 2.83E-01 |
| 1.07E+04 | 3.16E-01 | 3.47E-01 | 3.07E-01 | 3.44E-01 | 4.29E-01 | 1.28E-01 | 0.00E+00 | 5.82E-02 | 2.92E-01 |
| 1.09E+04 | 3.20E-01 | 3.49E-01 | 3.12E-01 | 3.44E-01 | 4.31E-01 | 1.16E-01 | 0.00E+00 | 6.55E-02 | 2.92E-01 |
| 1.12E+04 | 3.23E-01 | 3.45E-01 | 3.16E-01 | 3.52E-01 | 4.28E-01 | 1.01E-01 | 0.00E+00 | 7.67E-02 | 2.90E-01 |
| 1.14E+04 | 3.29E-01 | 3.53E-01 | 3.21E-01 | 3.53E-01 | 4.32E-01 | 9.44E-02 | 4.18E-03 | 7.71E-02 | 2.89E-01 |
| 1.16E+04 | 3.30E-01 | 3.54E-01 | 3.31E-01 | 3.58E-01 | 4.47E-01 | 1.03E-01 | 7.62E-04 | 8.22E-02 | 2.83E-01 |
| 1.19E+04 | 3.39E-01 | 3.41E-01 | 3.33E-01 | 3.63E-01 | 4.43E-01 | 1.15E-01 | 8.33E-03 | 8.11E-02 | 2.92E-01 |
| 1.21E+04 | 3.49E-01 | 3.45E-01 | 3.38E-01 | 3.70E-01 | 4.51E-01 | 1.23E-01 | 7.76E-03 | 8.75E-02 | 2.84E-01 |
| 1.24E+04 | 3.51E-01 | 3.45E-01 | 3.41E-01 | 3.75E-01 | 4.66E-01 | 1.33E-01 | 1.02E-02 | 9.20E-02 | 3.06E-01 |
| 1.26E+04 | 3.52E-01 | 3.61E-01 | 3.45E-01 | 3.78E-01 | 4.71E-01 | 1.30E-01 | 3.41E-03 | 8.99E-02 | 3.21E-01 |
| 1.29E+04 | 3.58E-01 | 3.70E-01 | 3.46E-01 | 3.87E-01 | 4.79E-01 | 1.54E-01 | 1.22E-02 | 1.10E-01 | 3.13E-01 |
| 1.31E+04 | 3.56E-01 | 3.72E-01 | 3.50E-01 | 3.91E-01 | 4.82E-01 | 1.66E-01 | 6.60E-03 | 1.10E-01 | 3.08E-01 |
| 1.34E+04 | 3.62E-01 | 3.82E-01 | 3.56E-01 | 3.92E-01 | 4.83E-01 | 1.62E-01 | 1.53E-02 | 1.14E-01 | 3.11E-01 |
| 1.37E+04 | 3.64E-01 | 3.82E-01 | 3.65E-01 | 3.96E-01 | 4.96E-01 | 1.57E-01 | 3.05E-02 | 1.16E-01 | 3.21E-01 |
| 1.40E+04 | 3.70E-01 | 3.82E-01 | 3.70E-01 | 3.98E-01 | 5.01E-01 | 1.60E-01 | 2.81E-02 | 1.11E-01 | 3.29E-01 |
| 1.42E+04 | 3.73E-01 | 3.74E-01 | 3.67E-01 | 4.02E-01 | 5.14E-01 | 1.79E-01 | 3.99E-02 | 1.18E-01 | 3.33E-01 |
| 1.45E+04 | 3.80E-01 | 3.90E-01 | 3.71E-01 | 4.03E-01 | 5.18E-01 | 1.72E-01 | 4.51E-02 | 1.21E-01 | 3.38E-01 |
| 1.48E+04 | 3.87E-01 | 4.00E-01 | 3.77E-01 | 4.08E-01 | 5.13E-01 | 1.74E-01 | 4.20E-02 | 1.26E-01 | 3.40E-01 |
| 1.51E+04 | 3.99E-01 | 3.95E-01 | 3.82E-01 | 4.20E-01 | 5.21E-01 | 1.92E-01 | 5.19E-02 | 1.24E-01 | 3.44E-01 |
| 1.54E+04 | 3.93E-01 | 4.07E-01 | 3.78E-01 | 4.29E-01 | 5.23E-01 | 2.00E-01 | 4.67E-02 | 1.19E-01 | 3.32E-01 |
| 1.58E+04 | 4.01E-01 | 4.11E-01 | 3.80E-01 | 4.29E-01 | 5.27E-01 | 1.87E-01 | 5.72E-02 | 1.27E-01 | 3.26E-01 |
| 1.61E+04 | 4.12E-01 | 4.13E-01 | 3.89E-01 | 4.33E-01 | 5.34E-01 | 1.89E-01 | 6.14E-02 | 1.33E-01 | 3.28E-01 |
| 1.64E+04 | 4.11E-01 | 4.20E-01 | 4.00E-01 | 4.41E-01 | 5.45E-01 | 2.16E-01 | 6.73E-02 | 1.32E-01 | 3.35E-01 |
| 1.67E+04 | 4.14E-01 | 4.21E-01 | 4.06E-01 | 4.41E-01 | 5.34E-01 | 2.19E-01 | 5.63E-02 | 1.43E-01 | 3.47E-01 |
| 1.71E+04 | 4.21E-01 | 4.28E-01 | 4.11E-01 | 4.46E-01 | 5.40E-01 | 2.00E-01 | 6.64E-02 | 1.51E-01 | 3.54E-01 |
| 1.74E+04 | 4.27E-01 | 4.42E-01 | 4.15E-01 | 4.58E-01 | 5.59E-01 | 2.15E-01 | 7.54E-02 | 1.45E-01 | 3.54E-01 |
| 1.78E+04 | 4.28E-01 | 4.40E-01 | 4.23E-01 | 4.63E-01 | 5.74E-01 | 2.36E-01 | 7.96E-02 | 1.46E-01 | 3.68E-01 |
| 1.82E+04 | 4.36E-01 | 4.41E-01 | 4.26E-01 | 4.56E-01 | 5.78E-01 | 2.55E-01 | 8.00E-02 | 1.61E-01 | 3.67E-01 |
| 1.85E+04 | 4.43E-01 | 4.57E-01 | 4.33E-01 | 4.58E-01 | 5.74E-01 | 2.38E-01 | 7.67E-02 | 1.55E-01 | 3.68E-01 |
| 1.89E+04 | 4.49E-01 | 4.60E-01 | 4.36E-01 | 4.66E-01 | 5.88E-01 | 2.27E-01 | 8.96E-02 | 1.52E-01 | 3.71E-01 |
| 1.93E+04 | 4.49E-01 | 4.62E-01 | 4.43E-01 | 4.70E-01 | 5.99E-01 | 2.53E-01 | 8.65E-02 | 1.60E-01 | 3.71E-01 |
| 1.97E+04 | 4.54E-01 | 4.67E-01 | 4.51E-01 | 4.75E-01 | 5.87E-01 | 2.64E-01 | 9.47E-02 | 1.67E-01 | 3.73E-01 |
| 2.01E+04 | 4.62E-01 | 4.68E-01 | 4.52E-01 | 4.84E-01 | 5.96E-01 | 2.74E-01 | 9.81E-02 | 1.69E-01 | 3.77E-01 |
| 2.05E+04 | 4.69E-01 | 4.64E-01 | 4.55E-01 | 4.88E-01 | 6.17E-01 | 2.56E-01 | 1.03E-01 | 1.73E-01 | 3.92E-01 |
| 2.09E+04 | 4.69E-01 | 4.75E-01 | 4.62E-01 | 4.91E-01 | 6.28E-01 | 2.67E-01 | 1.11E-01 | 1.67E-01 | 3.90E-01 |
| 2.13E+04 | 4.71E-01 | 4.78E-01 | 4.62E-01 | 4.95E-01 | 6.13E-01 | 2.86E-01 | 1.05E-01 | 1.85E-01 | 3.86E-01 |
| 2.18E+04 | 4.78E-01 | 4.83E-01 | 4.69E-01 | 4.99E-01 | 6.18E-01 | 2.80E-01 | 1.10E-01 | 1.83E-01 | 3.92E-01 |
| 2.22E+04 | 4.83E-01 | 5.00E-01 | 4.74E-01 | 5.08E-01 | 6.31E-01 | 2.86E-01 | 1.26E-01 | 1.88E-01 | 3.93E-01 |
| 2.27E+04 | 4.98E-01 | 4.89E-01 | 4.77E-01 | 5.15E-01 | 6.38E-01 | 3.00E-01 | 1.45E-01 | 1.80E-01 | 4.02E-01 |
| 2.31E+04 | 5.01E-01 | 4.88E-01 | 4.82E-01 | 5.11E-01 | 6.40E-01 | 3.19E-01 | 1.40E-01 | 2.03E-01 | 4.09E-01 |
| 2.36E+04 | 5.04E-01 | 5.04E-01 | 4.84E-01 | 5.13E-01 | 6.41E-01 | 3.14E-01 | 1.35E-01 | 1.99E-01 | 4.06E-01 |
| 2.41E+04 | 5.04E-01 | 5.08E-01 | 4.94E-01 | 5.19E-01 | 6.46E-01 | 3.14E-01 | 1.43E-01 | 2.05E-01 | 4.10E-01 |
| 2.46E+04 | 5.09E-01 | 5.08E-01 | 4.99E-01 | 5.24E-01 | 6.41E-01 | 3.30E-01 | 1.49E-01 | 2.23E-01 | 4.16E-01 |
| 2.51E+04 | 5.15E-01 | 5.23E-01 | 5.05E-01 | 5.33E-01 | 6.50E-01 | 3.25E-01 | 1.51E-01 | 2.16E-01 | 4.10E-01 |
| 2.56E+04 | 5.19E-01 | 5.31E-01 | 5.11E-01 | 5.40E-01 | 6.55E-01 | 3.20E-01 | 1.60E-01 | 2.25E-01 | 4.23E-01 |
| 2.61E+04 | 5.27E-01 | 5.32E-01 | 5.18E-01 | 5.43E-01 | 6.63E-01 | 3.18E-01 | 1.60E-01 | 2.39E-01 | 4.24E-01 |
| 2.67E+04 | 5.35E-01 | 5.28E-01 | 5.22E-01 | 5.45E-01 | 6.69E-01 | 3.21E-01 | 1.68E-01 | 2.29E-01 | 4.28E-01 |
| 2.72E+04 | 5.34E-01 | 5.25E-01 | 5.23E-01 | 5.49E-01 | 6.66E-01 | 3.45E-01 | 1.84E-01 | 2.27E-01 | 4.26E-01 |
| 2.78E+04 | 5.36E-01 | 5.38E-01 | 5.31E-01 | 5.43E-01 | 6.72E-01 | 3.46E-01 | 1.84E-01 | 2.36E-01 | 4.19E-01 |
| 2.83E+04 | 5.44E-01 | 5.53E-01 | 5.34E-01 | 5.49E-01 | 6.78E-01 | 3.56E-01 | 1.80E-01 | 2.35E-01 | 4.31E-01 |
| 2.89E+04 | 5.52E-01 | 5.52E-01 | 5.34E-01 | 5.55E-01 | 6.73E-01 | 3.53E-01 | 1.95E-01 | 2.41E-01 | 4.32E-01 |
| 2.95E+04 | 5.55E-01 | 5.72E-01 | 5.40E-01 | 5.57E-01 | 6.78E-01 | 3.74E-01 | 1.95E-01 | 2.56E-01 | 4.32E-01 |
| 3.01E+04 | 5.61E-01 | 5.86E-01 | 5.47E-01 | 5.62E-01 | 6.97E-01 | 3.68E-01 | 1.97E-01 | 2.53E-01 | 4.42E-01 |
| 3.07E+04 | 5.66E-01 | 5.79E-01 | 5.51E-01 | 5.58E-01 | 6.92E-01 | 3.52E-01 | 2.05E-01 | 2.71E-01 | 4.44E-01 |
| 3.13E+04 | 5.73E-01 | 5.84E-01 | 5.57E-01 | 5.55E-01 | 6.94E-01 | 3.77E-01 | 1.95E-01 | 2.76E-01 | 4.40E-01 |
| 3.20E+04 | 5.82E-01 | 5.85E-01 | 5.64E-01 | 5.64E-01 | 7.03E-01 | 3.85E-01 | 2.11E-01 | 2.75E-01 | 4.52E-01 |
| 3.26E+04 | 5.87E-01 | 5.85E-01 | 5.68E-01 | 5.72E-01 | 7.03E-01 | 3.81E-01 | 2.27E-01 | 2.63E-01 | 4.57E-01 |
| 3.33E+04 | 5.84E-01 | 5.96E-01 | 5.72E-01 | 5.78E-01 | 7.08E-01 | 3.88E-01 | 2.30E-01 | 2.85E-01 | 4.53E-01 |
| 3.40E+04 | 5.94E-01 | 6.00E-01 | 5.76E-01 | 5.92E-01 | 7.14E-01 | 3.85E-01 | 2.35E-01 | 2.89E-01 | 4.70E-01 |
| 3.47E+04 | 6.01E-01 | 6.12E-01 | 5.80E-01 | 5.98E-01 | 7.19E-01 | 3.95E-01 | 2.39E-01 | 2.86E-01 | 4.70E-01 |
| 3.54E+04 | 6.02E-01 | 6.19E-01 | 5.83E-01 | 6.00E-01 | 7.17E-01 | 4.15E-01 | 2.45E-01 | 2.90E-01 | 4.68E-01 |
| 3.61E+04 | 6.09E-01 | 6.06E-01 | 5.87E-01 | 6.05E-01 | 7.17E-01 | 4.17E-01 | 2.48E-01 | 3.13E-01 | 4.69E-01 |
| 3.69E+04 | 6.08E-01 | 6.00E-01 | 5.98E-01 | 6.09E-01 | 7.26E-01 | 4.36E-01 | 2.52E-01 | 3.23E-01 | 4.77E-01 |
| 3.76E+04 | 6.15E-01 | 6.07E-01 | 6.01E-01 | 6.16E-01 | 7.30E-01 | 4.51E-01 | 2.54E-01 | 3.24E-01 | 4.81E-01 |
| 3.84E+04 | 6.19E-01 | 6.10E-01 | 6.06E-01 | 6.26E-01 | 7.37E-01 | 4.41E-01 | 2.62E-01 | 3.19E-01 | 4.86E-01 |
| 3.92E+04 | 6.23E-01 | 6.21E-01 | 6.09E-01 | 6.24E-01 | 7.36E-01 | 4.50E-01 | 2.69E-01 | 3.22E-01 | 4.87E-01 |
| 4.00E+04 | 6.27E-01 | 6.29E-01 | 6.08E-01 | 6.33E-01 | 7.26E-01 | 4.88E-01 | 2.69E-01 | 3.28E-01 | 4.84E-01 |
| 4.08E+04 | 6.31E-01 | 6.29E-01 | 6.16E-01 | 6.33E-01 | 7.33E-01 | 4.73E-01 | 2.87E-01 | 3.27E-01 | 4.88E-01 |
| 4.16E+04 | 6.34E-01 | 6.37E-01 | 6.17E-01 | 6.35E-01 | 7.40E-01 | 4.82E-01 | 2.89E-01 | 3.40E-01 | 4.96E-01 |
| 4.25E+04 | 6.38E-01 | 6.44E-01 | 6.19E-01 | 6.44E-01 | 7.38E-01 | 4.78E-01 | 2.96E-01 | 3.51E-01 | 4.97E-01 |
| 4.33E+04 | 6.42E-01 | 6.48E-01 | 6.25E-01 | 6.43E-01 | 7.34E-01 | 4.99E-01 | 3.00E-01 | 3.53E-01 | 4.96E-01 |
| 4.42E+04 | 6.42E-01 | 6.48E-01 | 6.30E-01 | 6.45E-01 | 7.40E-01 | 5.05E-01 | 3.07E-01 | 3.63E-01 | 5.05E-01 |
| 4.51E+04 | 6.47E-01 | 6.53E-01 | 6.35E-01 | 6.49E-01 | 7.52E-01 | 4.84E-01 | 3.08E-01 | 3.60E-01 | 5.13E-01 |
| 4.60E+04 | 6.51E-01 | 6.66E-01 | 6.35E-01 | 6.46E-01 | 7.47E-01 | 4.94E-01 | 3.13E-01 | 3.71E-01 | 5.11E-01 |
| 4.70E+04 | 6.54E-01 | 6.68E-01 | 6.39E-01 | 6.53E-01 | 7.50E-01 | 5.18E-01 | 3.19E-01 | 3.74E-01 | 5.17E-01 |
| 4.79E+04 | 6.58E-01 | 6.56E-01 | 6.49E-01 | 6.61E-01 | 7.56E-01 | 5.15E-01 | 3.31E-01 | 3.70E-01 | 5.24E-01 |
| 4.89E+04 | 6.58E-01 | 6.63E-01 | 6.53E-01 | 6.55E-01 | 7.49E-01 | 5.44E-01 | 3.38E-01 | 3.84E-01 | 5.24E-01 |
| 4.99E+04 | 6.60E-01 | 6.72E-01 | 6.56E-01 | 6.56E-01 | 7.50E-01 | 5.25E-01 | 3.41E-01 | 3.90E-01 | 5.21E-01 |
| 5.09E+04 | 6.66E-01 | 6.61E-01 | 6.60E-01 | 6.59E-01 | 7.50E-01 | 5.05E-01 | 3.44E-01 | 3.92E-01 | 5.16E-01 |
| 5.20E+04 | 6.66E-01 | 6.60E-01 | 6.59E-01 | 6.59E-01 | 7.48E-01 | 4.95E-01 | 3.52E-01 | 3.87E-01 | 5.13E-01 |
| 5.30E+04 | 6.70E-01 | 6.78E-01 | 6.61E-01 | 6.58E-01 | 7.48E-01 | 5.22E-01 | 3.55E-01 | 4.09E-01 | 5.16E-01 |
| 5.41E+04 | 6.68E-01 | 6.85E-01 | 6.64E-01 | 6.60E-01 | 7.39E-01 | 5.55E-01 | 3.64E-01 | 4.18E-01 | 5.23E-01 |
| 5.52E+04 | 6.71E-01 | 6.91E-01 | 6.66E-01 | 6.62E-01 | 7.34E-01 | 5.34E-01 | 3.67E-01 | 4.12E-01 | 5.29E-01 |
| 5.64E+04 | 6.77E-01 | 6.82E-01 | 6.68E-01 | 6.56E-01 | 7.33E-01 | 5.63E-01 | 3.74E-01 | 4.32E-01 | 5.23E-01 |
| 5.75E+04 | 6.75E-01 | 6.78E-01 | 6.66E-01 | 6.68E-01 | 7.36E-01 | 5.53E-01 | 3.77E-01 | 4.23E-01 | 5.12E-01 |
| 5.87E+04 | 6.77E-01 | 6.85E-01 | 6.66E-01 | 6.71E-01 | 7.40E-01 | 5.60E-01 | 3.83E-01 | 4.28E-01 | 5.14E-01 |
| 5.99E+04 | 6.80E-01 | 6.86E-01 | 6.73E-01 | 6.76E-01 | 7.32E-01 | 5.67E-01 | 3.93E-01 | 4.43E-01 | 5.20E-01 |
| 6.11E+04 | 6.79E-01 | 6.91E-01 | 6.73E-01 | 6.79E-01 | 7.34E-01 | 5.69E-01 | 3.95E-01 | 4.16E-01 | 5.17E-01 |
| 6.24E+04 | 6.77E-01 | 6.96E-01 | 6.72E-01 | 6.81E-01 | 7.27E-01 | 5.96E-01 | 4.01E-01 | 4.36E-01 | 5.19E-01 |
| 6.36E+04 | 6.79E-01 | 6.95E-01 | 6.76E-01 | 6.82E-01 | 7.15E-01 | 6.07E-01 | 4.11E-01 | 4.50E-01 | 5.24E-01 |
| 6.49E+04 | 6.82E-01 | 6.85E-01 | 6.78E-01 | 6.83E-01 | 7.07E-01 | 5.91E-01 | 4.16E-01 | 4.76E-01 | 5.27E-01 |
| 6.63E+04 | 6.74E-01 | 6.88E-01 | 6.77E-01 | 6.71E-01 | 7.10E-01 | 6.08E-01 | 4.20E-01 | 4.72E-01 | 5.24E-01 |
| 6.76E+04 | 6.75E-01 | 6.91E-01 | 6.77E-01 | 6.68E-01 | 7.08E-01 | 5.88E-01 | 4.29E-01 | 4.62E-01 | 5.19E-01 |
| 6.90E+04 | 6.78E-01 | 6.94E-01 | 6.76E-01 | 6.79E-01 | 6.93E-01 | 6.09E-01 | 4.42E-01 | 4.71E-01 | 5.16E-01 |
| 7.04E+04 | 6.76E-01 | 6.97E-01 | 6.75E-01 | 6.65E-01 | 6.91E-01 | 6.07E-01 | 4.43E-01 | 4.89E-01 | 5.08E-01 |
| 7.18E+04 | 6.75E-01 | 6.88E-01 | 6.76E-01 | 6.73E-01 | 6.94E-01 | 6.21E-01 | 4.42E-01 | 4.91E-01 | 5.20E-01 |
| 7.33E+04 | 6.79E-01 | 6.86E-01 | 6.73E-01 | 6.69E-01 | 6.85E-01 | 6.38E-01 | 4.45E-01 | 4.92E-01 | 5.36E-01 |
| 7.48E+04 | 6.78E-01 | 6.96E-01 | 6.72E-01 | 6.63E-01 | 6.76E-01 | 6.02E-01 | 4.54E-01 | 4.91E-01 | 5.36E-01 |
| 7.63E+04 | 6.75E-01 | 6.99E-01 | 6.75E-01 | 6.63E-01 | 6.78E-01 | 6.19E-01 | 4.62E-01 | 4.91E-01 | 5.28E-01 |
| 7.79E+04 | 6.74E-01 | 6.98E-01 | 6.77E-01 | 6.55E-01 | 6.78E-01 | 6.10E-01 | 4.65E-01 | 5.06E-01 | 5.24E-01 |
| 7.95E+04 | 6.74E-01 | 6.90E-01 | 6.77E-01 | 6.63E-01 | 6.75E-01 | 6.34E-01 | 4.72E-01 | 5.06E-01 | 5.19E-01 |
| 8.11E+04 | 6.71E-01 | 6.72E-01 | 6.74E-01 | 6.65E-01 | 6.63E-01 | 6.29E-01 | 4.84E-01 | 4.86E-01 | 5.17E-01 |
| 8.28E+04 | 6.68E-01 | 6.76E-01 | 6.72E-01 | 6.67E-01 | 6.56E-01 | 6.15E-01 | 4.84E-01 | 5.26E-01 | 5.14E-01 |
| 8.45E+04 | 6.72E-01 | 6.77E-01 | 6.72E-01 | 6.62E-01 | 6.51E-01 | 6.41E-01 | 4.89E-01 | 5.27E-01 | 5.19E-01 |
| 8.62E+04 | 6.67E-01 | 6.68E-01 | 6.71E-01 | 6.45E-01 | 6.38E-01 | 6.45E-01 | 4.93E-01 | 5.04E-01 | 5.29E-01 |
| 8.80E+04 | 6.62E-01 | 6.67E-01 | 6.66E-01 | 6.58E-01 | 6.33E-01 | 6.58E-01 | 5.00E-01 | 5.34E-01 | 5.19E-01 |
| 8.98E+04 | 6.63E-01 | 6.73E-01 | 6.64E-01 | 6.53E-01 | 6.30E-01 | 6.94E-01 | 5.02E-01 | 5.41E-01 | 5.11E-01 |
| 9.16E+04 | 6.64E-01 | 6.74E-01 | 6.66E-01 | 6.53E-01 | 6.21E-01 | 6.55E-01 | 5.08E-01 | 5.62E-01 | 5.15E-01 |
| 9.35E+04 | 6.59E-01 | 6.63E-01 | 6.66E-01 | 6.42E-01 | 6.17E-01 | 6.71E-01 | 5.23E-01 | 5.71E-01 | 5.24E-01 |
| 9.54E+04 | 6.52E-01 | 6.50E-01 | 6.59E-01 | 6.28E-01 | 6.11E-01 | 6.91E-01 | 5.21E-01 | 5.51E-01 | 5.21E-01 |
| 9.73E+04 | 6.50E-01 | 6.37E-01 | 6.54E-01 | 6.34E-01 | 5.97E-01 | 6.69E-01 | 5.26E-01 | 5.65E-01 | 5.10E-01 |
| 9.93E+04 | 6.45E-01 | 6.33E-01 | 6.54E-01 | 6.32E-01 | 5.86E-01 | 6.79E-01 | 5.33E-01 | 5.50E-01 | 5.00E-01 |
| 1.01E+05 | 6.41E-01 | 6.34E-01 | 6.52E-01 | 6.29E-01 | 5.81E-01 | 6.99E-01 | 5.37E-01 | 5.59E-01 | 5.00E-01 |
| 1.03E+05 | 6.40E-01 | 6.37E-01 | 6.51E-01 | 6.35E-01 | 5.77E-01 | 6.92E-01 | 5.45E-01 | 5.60E-01 | 5.07E-01 |
| 1.06E+05 | 6.38E-01 | 6.25E-01 | 6.48E-01 | 6.23E-01 | 5.70E-01 | 6.58E-01 | 5.58E-01 | 5.73E-01 | 5.04E-01 |
| 1.08E+05 | 6.33E-01 | 6.23E-01 | 6.45E-01 | 6.18E-01 | 5.59E-01 | 6.88E-01 | 5.64E-01 | 5.72E-01 | 5.01E-01 |
| 1.10E+05 | 6.26E-01 | 6.28E-01 | 6.39E-01 | 6.06E-01 | 5.54E-01 | 6.69E-01 | 5.66E-01 | 5.65E-01 | 5.00E-01 |
| 1.12E+05 | 6.20E-01 | 6.29E-01 | 6.30E-01 | 5.92E-01 | 5.49E-01 | 6.92E-01 | 5.67E-01 | 5.93E-01 | 5.09E-01 |
| 1.14E+05 | 6.17E-01 | 6.17E-01 | 6.29E-01 | 5.86E-01 | 5.42E-01 | 6.85E-01 | 5.79E-01 | 5.85E-01 | 5.08E-01 |
| 1.17E+05 | 6.11E-01 | 6.04E-01 | 6.24E-01 | 5.67E-01 | 5.24E-01 | 6.77E-01 | 5.90E-01 | 5.79E-01 | 4.98E-01 |
| 1.19E+05 | 6.06E-01 | 5.88E-01 | 6.14E-01 | 5.65E-01 | 5.15E-01 | 6.69E-01 | 5.87E-01 | 5.95E-01 | 4.93E-01 |
| 1.22E+05 | 6.03E-01 | 5.79E-01 | 6.12E-01 | 5.71E-01 | 5.10E-01 | 7.20E-01 | 5.83E-01 | 6.01E-01 | 4.86E-01 |
| 1.24E+05 | 5.96E-01 | 5.85E-01 | 6.14E-01 | 5.58E-01 | 5.08E-01 | 6.86E-01 | 5.90E-01 | 5.94E-01 | 4.90E-01 |
| 1.27E+05 | 5.94E-01 | 5.88E-01 | 6.10E-01 | 5.48E-01 | 4.90E-01 | 7.08E-01 | 5.98E-01 | 6.21E-01 | 4.97E-01 |
| 1.29E+05 | 5.91E-01 | 5.77E-01 | 6.06E-01 | 5.54E-01 | 4.81E-01 | 6.99E-01 | 6.01E-01 | 5.94E-01 | 4.90E-01 |
| 1.32E+05 | 5.84E-01 | 5.56E-01 | 5.98E-01 | 5.54E-01 | 4.67E-01 | 6.91E-01 | 6.04E-01 | 6.37E-01 | 4.78E-01 |
| 1.35E+05 | 5.78E-01 | 5.53E-01 | 5.91E-01 | 5.47E-01 | 4.58E-01 | 6.83E-01 | 6.03E-01 | 6.14E-01 | 4.82E-01 |
| 1.37E+05 | 5.70E-01 | 5.67E-01 | 5.89E-01 | 5.32E-01 | 4.67E-01 | 7.05E-01 | 6.01E-01 | 6.32E-01 | 4.77E-01 |
| 1.40E+05 | 5.64E-01 | 5.58E-01 | 5.82E-01 | 5.37E-01 | 4.46E-01 | 7.17E-01 | 6.02E-01 | 6.29E-01 | 4.67E-01 |
| 1.43E+05 | 5.58E-01 | 5.51E-01 | 5.79E-01 | 5.29E-01 | 4.33E-01 | 7.31E-01 | 5.99E-01 | 6.13E-01 | 4.68E-01 |
| 1.46E+05 | 5.55E-01 | 5.40E-01 | 5.74E-01 | 5.22E-01 | 4.32E-01 | 6.99E-01 | 6.11E-01 | 6.23E-01 | 4.58E-01 |
| 1.49E+05 | 5.53E-01 | 5.28E-01 | 5.66E-01 | 5.25E-01 | 4.19E-01 | 7.07E-01 | 6.14E-01 | 6.34E-01 | 4.51E-01 |
| 1.52E+05 | 5.44E-01 | 5.14E-01 | 5.62E-01 | 5.16E-01 | 4.07E-01 | 6.83E-01 | 6.16E-01 | 6.32E-01 | 4.55E-01 |
| 1.55E+05 | 5.35E-01 | 5.03E-01 | 5.54E-01 | 5.12E-01 | 3.96E-01 | 6.90E-01 | 6.19E-01 | 6.34E-01 | 4.47E-01 |
| 1.58E+05 | 5.31E-01 | 5.07E-01 | 5.53E-01 | 5.08E-01 | 3.85E-01 | 6.87E-01 | 6.33E-01 | 6.50E-01 | 4.50E-01 |
| 1.61E+05 | 5.25E-01 | 5.00E-01 | 5.46E-01 | 5.01E-01 | 3.70E-01 | 7.01E-01 | 6.38E-01 | 6.44E-01 | 4.50E-01 |
| 1.65E+05 | 5.16E-01 | 4.96E-01 | 5.38E-01 | 4.99E-01 | 3.70E-01 | 6.93E-01 | 6.40E-01 | 6.44E-01 | 4.36E-01 |
| 1.68E+05 | 5.15E-01 | 4.93E-01 | 5.33E-01 | 4.84E-01 | 3.59E-01 | 7.07E-01 | 6.49E-01 | 6.52E-01 | 4.33E-01 |
| 1.71E+05 | 5.13E-01 | 4.76E-01 | 5.27E-01 | 4.70E-01 | 3.53E-01 | 7.15E-01 | 6.54E-01 | 6.52E-01 | 4.26E-01 |
| 1.75E+05 | 5.06E-01 | 4.61E-01 | 5.17E-01 | 4.57E-01 | 3.50E-01 | 7.10E-01 | 6.60E-01 | 6.72E-01 | 4.06E-01 |
| 1.79E+05 | 4.94E-01 | 4.62E-01 | 5.07E-01 | 4.54E-01 | 3.27E-01 | 7.09E-01 | 6.64E-01 | 6.73E-01 | 4.16E-01 |
| 1.82E+05 | 4.88E-01 | 4.47E-01 | 5.02E-01 | 4.58E-01 | 3.20E-01 | 6.85E-01 | 6.71E-01 | 6.62E-01 | 4.20E-01 |
| 1.86E+05 | 4.86E-01 | 4.39E-01 | 5.03E-01 | 4.33E-01 | 3.05E-01 | 6.73E-01 | 6.77E-01 | 6.51E-01 | 4.10E-01 |
| 1.90E+05 | 4.80E-01 | 4.39E-01 | 4.91E-01 | 4.42E-01 | 2.93E-01 | 6.77E-01 | 6.76E-01 | 6.78E-01 | 4.02E-01 |
| 1.94E+05 | 4.73E-01 | 4.33E-01 | 4.88E-01 | 4.30E-01 | 2.90E-01 | 7.00E-01 | 6.76E-01 | 6.69E-01 | 3.90E-01 |
| 1.98E+05 | 4.66E-01 | 4.20E-01 | 4.79E-01 | 4.28E-01 | 2.91E-01 | 6.96E-01 | 6.86E-01 | 6.75E-01 | 4.03E-01 |
| 2.02E+05 | 4.59E-01 | 4.18E-01 | 4.71E-01 | 4.27E-01 | 2.85E-01 | 6.83E-01 | 6.92E-01 | 6.97E-01 | 3.97E-01 |
| 2.06E+05 | 4.50E-01 | 4.12E-01 | 4.66E-01 | 4.09E-01 | 2.68E-01 | 6.91E-01 | 6.89E-01 | 6.63E-01 | 3.94E-01 |
| 2.10E+05 | 4.45E-01 | 3.96E-01 | 4.64E-01 | 3.95E-01 | 2.55E-01 | 6.87E-01 | 6.89E-01 | 6.95E-01 | 3.95E-01 |
| 2.14E+05 | 4.38E-01 | 3.82E-01 | 4.58E-01 | 3.99E-01 | 2.61E-01 | 6.64E-01 | 6.94E-01 | 6.80E-01 | 3.76E-01 |
| 2.19E+05 | 4.27E-01 | 3.73E-01 | 4.45E-01 | 3.83E-01 | 2.57E-01 | 6.73E-01 | 7.05E-01 | 6.88E-01 | 3.78E-01 |
| 2.23E+05 | 4.22E-01 | 3.66E-01 | 4.31E-01 | 3.76E-01 | 2.44E-01 | 6.55E-01 | 7.01E-01 | 6.79E-01 | 3.81E-01 |
| 2.28E+05 | 4.19E-01 | 3.73E-01 | 4.20E-01 | 3.65E-01 | 2.38E-01 | 6.86E-01 | 6.99E-01 | 7.09E-01 | 3.91E-01 |
| 2.32E+05 | 4.10E-01 | 3.60E-01 | 4.24E-01 | 3.66E-01 | 2.23E-01 | 6.71E-01 | 6.99E-01 | 7.00E-01 | 3.72E-01 |
| 2.37E+05 | 4.01E-01 | 3.38E-01 | 4.15E-01 | 3.48E-01 | 2.15E-01 | 6.81E-01 | 6.98E-01 | 7.07E-01 | 3.77E-01 |
| 2.42E+05 | 3.95E-01 | 3.25E-01 | 4.19E-01 | 3.64E-01 | 2.05E-01 | 6.98E-01 | 7.02E-01 | 6.97E-01 | 3.74E-01 |
| 2.47E+05 | 3.88E-01 | 3.20E-01 | 4.14E-01 | 3.38E-01 | 1.92E-01 | 6.70E-01 | 7.02E-01 | 6.86E-01 | 3.76E-01 |
| 2.52E+05 | 3.82E-01 | 3.17E-01 | 4.02E-01 | 3.32E-01 | 1.92E-01 | 6.79E-01 | 7.06E-01 | 6.89E-01 | 3.82E-01 |
| 2.57E+05 | 3.79E-01 | 3.12E-01 | 3.95E-01 | 3.18E-01 | 1.90E-01 | 6.74E-01 | 7.13E-01 | 7.04E-01 | 3.71E-01 |
| 2.62E+05 | 3.70E-01 | 3.00E-01 | 3.92E-01 | 3.17E-01 | 1.79E-01 | 6.41E-01 | 7.23E-01 | 6.91E-01 | 3.62E-01 |
| 2.68E+05 | 3.60E-01 | 2.84E-01 | 3.77E-01 | 3.10E-01 | 1.62E-01 | 6.49E-01 | 7.28E-01 | 6.88E-01 | 3.59E-01 |
| 2.73E+05 | 3.55E-01 | 2.76E-01 | 3.71E-01 | 3.04E-01 | 1.60E-01 | 6.52E-01 | 7.18E-01 | 6.97E-01 | 3.54E-01 |
| 2.79E+05 | 3.49E-01 | 2.65E-01 | 3.67E-01 | 3.07E-01 | 1.57E-01 | 6.93E-01 | 7.19E-01 | 6.91E-01 | 3.45E-01 |
| 2.84E+05 | 3.40E-01 | 2.71E-01 | 3.58E-01 | 2.90E-01 | 1.35E-01 | 6.19E-01 | 7.19E-01 | 6.91E-01 | 3.40E-01 |
| 2.90E+05 | 3.27E-01 | 2.68E-01 | 3.52E-01 | 2.75E-01 | 1.31E-01 | 6.22E-01 | 7.18E-01 | 7.08E-01 | 3.40E-01 |
| 2.96E+05 | 3.23E-01 | 2.61E-01 | 3.49E-01 | 2.68E-01 | 1.28E-01 | 6.15E-01 | 7.15E-01 | 6.97E-01 | 3.29E-01 |
| 3.02E+05 | 3.18E-01 | 2.54E-01 | 3.42E-01 | 2.62E-01 | 1.22E-01 | 6.46E-01 | 7.20E-01 | 6.92E-01 | 3.42E-01 |
| 3.08E+05 | 3.10E-01 | 2.43E-01 | 3.32E-01 | 2.57E-01 | 1.11E-01 | 6.32E-01 | 7.20E-01 | 6.93E-01 | 3.36E-01 |
| 3.15E+05 | 3.03E-01 | 2.27E-01 | 3.27E-01 | 2.51E-01 | 1.09E-01 | 6.11E-01 | 7.18E-01 | 6.78E-01 | 3.24E-01 |
| 3.21E+05 | 2.94E-01 | 2.26E-01 | 3.18E-01 | 2.46E-01 | 1.01E-01 | 6.19E-01 | 7.24E-01 | 6.85E-01 | 3.24E-01 |
| 3.28E+05 | 2.87E-01 | 2.17E-01 | 3.10E-01 | 2.30E-01 | 1.05E-01 | 5.96E-01 | 7.26E-01 | 6.96E-01 | 3.31E-01 |
| 3.34E+05 | 2.81E-01 | 1.98E-01 | 3.04E-01 | 2.35E-01 | 9.96E-02 | 6.07E-01 | 7.21E-01 | 6.94E-01 | 3.22E-01 |
| 3.41E+05 | 2.78E-01 | 1.90E-01 | 2.94E-01 | 2.22E-01 | 8.80E-02 | 5.96E-01 | 7.16E-01 | 6.83E-01 | 3.15E-01 |
| 3.48E+05 | 2.71E-01 | 1.92E-01 | 2.87E-01 | 2.06E-01 | 8.16E-02 | 5.84E-01 | 7.16E-01 | 6.83E-01 | 3.04E-01 |
| 3.55E+05 | 2.63E-01 | 1.96E-01 | 2.86E-01 | 2.05E-01 | 7.19E-02 | 6.01E-01 | 7.13E-01 | 6.84E-01 | 2.94E-01 |
| 3.63E+05 | 2.56E-01 | 1.88E-01 | 2.78E-01 | 1.94E-01 | 7.26E-02 | 5.44E-01 | 7.13E-01 | 6.75E-01 | 2.95E-01 |
| 3.70E+05 | 2.49E-01 | 1.71E-01 | 2.71E-01 | 1.88E-01 | 6.53E-02 | 5.87E-01 | 7.14E-01 | 6.75E-01 | 3.10E-01 |
| 3.77E+05 | 2.42E-01 | 1.68E-01 | 2.68E-01 | 1.91E-01 | 6.74E-02 | 5.50E-01 | 7.13E-01 | 6.71E-01 | 3.06E-01 |
| 3.85E+05 | 2.37E-01 | 1.55E-01 | 2.59E-01 | 1.84E-01 | 7.04E-02 | 5.55E-01 | 7.16E-01 | 6.81E-01 | 2.92E-01 |
| 3.93E+05 | 2.34E-01 | 1.53E-01 | 2.50E-01 | 1.85E-01 | 3.80E-02 | 5.23E-01 | 7.15E-01 | 6.74E-01 | 2.89E-01 |
| 4.01E+05 | 2.24E-01 | 1.50E-01 | 2.45E-01 | 1.64E-01 | 4.22E-02 | 5.37E-01 | 7.13E-01 | 6.85E-01 | 2.81E-01 |
| 4.09E+05 | 2.14E-01 | 1.44E-01 | 2.30E-01 | 1.64E-01 | 5.31E-02 | 5.46E-01 | 7.17E-01 | 6.72E-01 | 2.86E-01 |
| 4.18E+05 | 2.13E-01 | 1.42E-01 | 2.26E-01 | 1.50E-01 | 4.08E-02 | 5.51E-01 | 7.14E-01 | 6.60E-01 | 2.86E-01 |
| 4.26E+05 | 2.08E-01 | 1.33E-01 | 2.20E-01 | 1.45E-01 | 3.10E-02 | 5.31E-01 | 7.06E-01 | 6.64E-01 | 2.69E-01 |
| 4.35E+05 | 2.03E-01 | 1.20E-01 | 2.18E-01 | 1.41E-01 | 1.69E-02 | 5.09E-01 | 7.02E-01 | 6.71E-01 | 2.73E-01 |
| 4.44E+05 | 1.99E-01 | 1.23E-01 | 2.12E-01 | 1.45E-01 | 2.40E-02 | 5.05E-01 | 6.96E-01 | 6.57E-01 | 2.69E-01 |
| 4.53E+05 | 1.90E-01 | 1.21E-01 | 2.08E-01 | 1.25E-01 | 3.46E-02 | 5.18E-01 | 6.91E-01 | 6.53E-01 | 2.83E-01 |
| 4.62E+05 | 1.83E-01 | 1.06E-01 | 2.02E-01 | 1.25E-01 | 2.70E-02 | 5.02E-01 | 6.87E-01 | 6.50E-01 | 2.69E-01 |
| 4.72E+05 | 1.78E-01 | 1.05E-01 | 1.93E-01 | 1.12E-01 | 1.26E-02 | 4.95E-01 | 6.81E-01 | 6.37E-01 | 2.65E-01 |
| 4.81E+05 | 1.72E-01 | 9.88E-02 | 1.87E-01 | 1.20E-01 | 9.23E-03 | 5.01E-01 | 6.82E-01 | 6.34E-01 | 2.68E-01 |
| 4.91E+05 | 1.67E-01 | 8.27E-02 | 1.85E-01 | 1.04E-01 | 1.66E-02 | 4.84E-01 | 6.79E-01 | 6.25E-01 | 2.60E-01 |
| 5.01E+05 | 1.60E-01 | 8.39E-02 | 1.80E-01 | 1.04E-01 | 7.47E-03 | 4.95E-01 | 6.64E-01 | 6.27E-01 | 2.61E-01 |
| 5.11E+05 | 1.55E-01 | 8.35E-02 | 1.72E-01 | 1.12E-01 | 1.37E-02 | 4.78E-01 | 6.56E-01 | 6.36E-01 | 2.43E-01 |
| 5.22E+05 | 1.51E-01 | 8.08E-02 | 1.69E-01 | 9.38E-02 | 2.49E-02 | 4.79E-01 | 6.55E-01 | 6.21E-01 | 2.58E-01 |
| 5.32E+05 | 1.49E-01 | 8.02E-02 | 1.61E-01 | 8.12E-02 | 1.15E-02 | 4.79E-01 | 6.58E-01 | 6.15E-01 | 2.40E-01 |
| 5.43E+05 | 1.46E-01 | 7.27E-02 | 1.53E-01 | 7.96E-02 | 1.41E-02 | 4.64E-01 | 6.56E-01 | 6.17E-01 | 2.41E-01 |
| 5.54E+05 | 1.38E-01 | 5.74E-02 | 1.46E-01 | 7.11E-02 | 2.98E-03 | 4.22E-01 | 6.57E-01 | 6.08E-01 | 2.48E-01 |
| 5.66E+05 | 1.36E-01 | 5.74E-02 | 1.47E-01 | 7.89E-02 | 0.00E+00 | 4.26E-01 | 6.57E-01 | 5.94E-01 | 2.46E-01 |
| 5.77E+05 | 1.30E-01 | 5.52E-02 | 1.41E-01 | 7.26E-02 | 0.00E+00 | 4.17E-01 | 6.55E-01 | 6.04E-01 | 2.39E-01 |
| 5.89E+05 | 1.23E-01 | 4.67E-02 | 1.34E-01 | 6.49E-02 | 0.00E+00 | 4.28E-01 | 6.59E-01 | 6.19E-01 | 2.39E-01 |
| 6.01E+05 | 1.23E-01 | 5.98E-02 | 1.25E-01 | 6.75E-02 | 2.31E-04 | 4.14E-01 | 6.54E-01 | 6.10E-01 | 2.28E-01 |
| 6.13E+05 | 1.19E-01 | 5.05E-02 | 1.22E-01 | 5.66E-02 | 9.32E-04 | 4.22E-01 | 6.57E-01 | 6.05E-01 | 2.18E-01 |
| 6.26E+05 | 1.15E-01 | 4.42E-02 | 1.25E-01 | 5.98E-02 | 7.73E-04 | 4.33E-01 | 6.62E-01 | 5.91E-01 | 2.13E-01 |
| 6.39E+05 | 1.11E-01 | 3.93E-02 | 1.13E-01 | 5.24E-02 | 3.21E-03 | 4.05E-01 | 6.56E-01 | 5.90E-01 | 2.10E-01 |
| 6.52E+05 | 1.02E-01 | 3.58E-02 | 1.09E-01 | 4.89E-02 | 0.00E+00 | 3.80E-01 | 6.44E-01 | 5.90E-01 | 2.12E-01 |
| 6.65E+05 | 9.70E-02 | 2.70E-02 | 1.06E-01 | 3.89E-02 | 0.00E+00 | 3.89E-01 | 6.37E-01 | 5.85E-01 | 2.05E-01 |
| 6.79E+05 | 9.06E-02 | 2.60E-02 | 1.04E-01 | 4.63E-02 | 0.00E+00 | 3.89E-01 | 6.35E-01 | 5.69E-01 | 2.07E-01 |
| 6.93E+05 | 8.76E-02 | 3.17E-02 | 9.66E-02 | 3.62E-02 | 0.00E+00 | 3.96E-01 | 6.30E-01 | 5.55E-01 | 1.96E-01 |
| 7.07E+05 | 8.55E-02 | 2.45E-02 | 9.10E-02 | 2.86E-02 | 0.00E+00 | 3.95E-01 | 6.19E-01 | 5.58E-01 | 2.05E-01 |
| 7.21E+05 | 7.89E-02 | 2.44E-02 | 8.83E-02 | 2.91E-02 | 0.00E+00 | 3.76E-01 | 6.12E-01 | 5.49E-01 | 1.86E-01 |
| 7.36E+05 | 7.67E-02 | 1.78E-02 | 8.16E-02 | 2.22E-02 | 0.00E+00 | 3.57E-01 | 6.12E-01 | 5.43E-01 | 1.82E-01 |
| 7.51E+05 | 7.79E-02 | 1.44E-02 | 7.42E-02 | 1.51E-02 | 0.00E+00 | 3.47E-01 | 6.09E-01 | 5.48E-01 | 1.88E-01 |
| 7.66E+05 | 7.68E-02 | 1.35E-02 | 7.71E-02 | 1.45E-02 | 0.00E+00 | 3.36E-01 | 5.98E-01 | 5.44E-01 | 1.88E-01 |
| 7.82E+05 | 6.84E-02 | 2.09E-03 | 8.05E-02 | 2.58E-02 | 0.00E+00 | 3.30E-01 | 5.85E-01 | 5.33E-01 | 1.91E-01 |
| 7.98E+05 | 5.63E-02 | 1.21E-02 | 6.83E-02 | 1.71E-02 | 0.00E+00 | 3.40E-01 | 5.76E-01 | 5.27E-01 | 1.65E-01 |
| 8.14E+05 | 5.37E-02 | 1.66E-02 | 6.14E-02 | 1.58E-02 | 0.00E+00 | 3.17E-01 | 5.71E-01 | 5.19E-01 | 1.66E-01 |
| 8.31E+05 | 5.58E-02 | 1.13E-03 | 6.35E-02 | 1.19E-02 | 0.00E+00 | 3.14E-01 | 5.69E-01 | 5.25E-01 | 1.81E-01 |
| 8.48E+05 | 5.23E-02 | 8.19E-04 | 6.19E-02 | 8.41E-03 | 0.00E+00 | 2.93E-01 | 5.64E-01 | 5.04E-01 | 1.69E-01 |
| 8.65E+05 | 5.17E-02 | 3.24E-03 | 5.15E-02 | 2.79E-03 | 0.00E+00 | 3.08E-01 | 5.59E-01 | 4.86E-01 | 1.68E-01 |
| 8.83E+05 | 4.82E-02 | 2.25E-04 | 5.32E-02 | 6.35E-04 | 0.00E+00 | 2.83E-01 | 5.52E-01 | 4.90E-01 | 1.66E-01 |
| 9.01E+05 | 4.62E-02 | 9.17E-04 | 5.32E-02 | 9.76E-03 | 0.00E+00 | 2.84E-01 | 5.43E-01 | 4.64E-01 | 1.59E-01 |
| 9.19E+05 | 4.40E-02 | 0.00E+00 | 4.60E-02 | 1.19E-02 | 0.00E+00 | 2.92E-01 | 5.35E-01 | 4.59E-01 | 1.43E-01 |
| 9.38E+05 | 3.88E-02 | 1.24E-04 | 4.44E-02 | 6.02E-03 | 0.00E+00 | 2.84E-01 | 5.25E-01 | 4.48E-01 | 1.49E-01 |
| 9.57E+05 | 3.88E-02 | 5.28E-04 | 3.54E-02 | 5.20E-04 | 0.00E+00 | 2.57E-01 | 5.19E-01 | 4.46E-01 | 1.44E-01 |
| 9.77E+05 | 3.10E-02 | 0.00E+00 | 3.43E-02 | 0.00E+00 | 0.00E+00 | 2.81E-01 | 5.16E-01 | 4.41E-01 | 1.52E-01 |
| 9.97E+05 | 2.42E-02 | 0.00E+00 | 3.89E-02 | 0.00E+00 | 0.00E+00 | 2.32E-01 | 5.03E-01 | 4.27E-01 | 1.49E-01 |
| 1.02E+06 | 2.10E-02 | 0.00E+00 | 2.60E-02 | 0.00E+00 | 0.00E+00 | 2.44E-01 | 4.88E-01 | 4.09E-01 | 1.58E-01 |
| 1.04E+06 | 2.64E-02 | 0.00E+00 | 2.61E-02 | 0.00E+00 | 0.00E+00 | 2.57E-01 | 4.84E-01 | 4.08E-01 | 1.43E-01 |
| 1.06E+06 | 2.19E-02 | 0.00E+00 | 2.75E-02 | 0.00E+00 | 0.00E+00 | 2.31E-01 | 4.79E-01 | 3.94E-01 | 1.40E-01 |
| 1.08E+06 | 8.41E-03 | 0.00E+00 | 2.12E-02 | 0.00E+00 | 0.00E+00 | 2.16E-01 | 4.65E-01 | 3.91E-01 | 1.37E-01 |
| 1.10E+06 | 1.01E-02 | 0.00E+00 | 2.27E-02 | 0.00E+00 | 0.00E+00 | 1.95E-01 | 4.57E-01 | 3.86E-01 | 1.35E-01 |
| 1.13E+06 | 1.56E-02 | 0.00E+00 | 1.28E-02 | 0.00E+00 | 0.00E+00 | 1.82E-01 | 4.49E-01 | 3.61E-01 | 1.18E-01 |
| 1.15E+06 | 1.78E-02 | 0.00E+00 | 1.76E-02 | 0.00E+00 | 0.00E+00 | 1.91E-01 | 4.37E-01 | 3.47E-01 | 1.11E-01 |
| 1.17E+06 | 1.41E-02 | 0.00E+00 | 1.78E-02 | 0.00E+00 | 0.00E+00 | 1.76E-01 | 4.24E-01 | 3.45E-01 | 1.31E-01 |
| 1.20E+06 | 7.47E-03 | 0.00E+00 | 1.37E-02 | 0.00E+00 | 0.00E+00 | 1.68E-01 | 4.11E-01 | 3.28E-01 | 1.26E-01 |
| 1.22E+06 | 5.03E-03 | 0.00E+00 | 1.10E-02 | 0.00E+00 | 0.00E+00 | 1.57E-01 | 4.01E-01 | 3.15E-01 | 1.31E-01 |
| 1.25E+06 | 6.72E-03 | 0.00E+00 | 8.66E-03 | 0.00E+00 | 0.00E+00 | 1.55E-01 | 3.96E-01 | 3.12E-01 | 1.02E-01 |
| 1.27E+06 | 8.22E-03 | 0.00E+00 | 4.74E-03 | 0.00E+00 | 0.00E+00 | 1.47E-01 | 3.88E-01 | 3.06E-01 | 1.08E-01 |
| 1.30E+06 | 2.05E-03 | 0.00E+00 | 1.17E-02 | 0.00E+00 | 0.00E+00 | 1.33E-01 | 3.75E-01 | 2.94E-01 | 9.60E-02 |
| 1.32E+06 | 0.00E+00 | 0.00E+00 | 7.97E-03 | 0.00E+00 | 0.00E+00 | 1.24E-01 | 3.73E-01 | 2.78E-01 | 8.33E-02 |
| 1.35E+06 | 0.00E+00 | 0.00E+00 | 9.33E-03 | 0.00E+00 | 0.00E+00 | 1.25E-01 | 3.61E-01 | 2.67E-01 | 1.02E-01 |
| 1.38E+06 | 7.86E-04 | 0.00E+00 | 7.12E-03 | 0.00E+00 | 0.00E+00 | 1.29E-01 | 3.54E-01 | 2.51E-01 | 1.06E-01 |
| 1.41E+06 | 1.37E-03 | 0.00E+00 | 0.00E+00 | 0.00E+00 | 0.00E+00 | 1.12E-01 | 3.45E-01 | 2.42E-01 | 1.08E-01 |
| 1.43E+06 | 0.00E+00 | 0.00E+00 | 0.00E+00 | 0.00E+00 | 0.00E+00 | 9.95E-02 | 3.27E-01 | 2.32E-01 | 8.52E-02 |
| 1.46E+06 | 6.01E-04 | 0.00E+00 | 0.00E+00 | 0.00E+00 | 0.00E+00 | 1.06E-01 | 3.19E-01 | 2.40E-01 | 9.41E-02 |
| 1.49E+06 | 1.08E-03 | 0.00E+00 | 0.00E+00 | 0.00E+00 | 0.00E+00 | 1.00E-01 | 3.13E-01 | 2.24E-01 | 1.06E-01 |
| 1.52E+06 | 0.00E+00 | 0.00E+00 | 0.00E+00 | 0.00E+00 | 0.00E+00 | 1.02E-01 | 3.01E-01 | 2.13E-01 | 8.13E-02 |
| 1.56E+06 | 0.00E+00 | 0.00E+00 | 0.00E+00 | 0.00E+00 | 0.00E+00 | 9.65E-02 | 2.91E-01 | 2.06E-01 | 8.47E-02 |
| 1.59E+06 | 0.00E+00 | 0.00E+00 | 0.00E+00 | 0.00E+00 | 0.00E+00 | 8.25E-02 | 2.82E-01 | 1.95E-01 | 7.97E-02 |
| 1.62E+06 | 0.00E+00 | 0.00E+00 | 0.00E+00 | 0.00E+00 | 0.00E+00 | 1.04E-01 | 2.71E-01 | 1.86E-01 | 9.27E-02 |
| 1.65E+06 | 0.00E+00 | 0.00E+00 | 0.00E+00 | 0.00E+00 | 0.00E+00 | 8.87E-02 | 2.62E-01 | 1.68E-01 | 7.63E-02 |
| 1.69E+06 | 0.00E+00 | 0.00E+00 | 0.00E+00 | 0.00E+00 | 0.00E+00 | 7.91E-02 | 2.54E-01 | 1.64E-01 | 6.70E-02 |
| 1.72E+06 | 0.00E+00 | 0.00E+00 | 0.00E+00 | 0.00E+00 | 0.00E+00 | 5.98E-02 | 2.43E-01 | 1.50E-01 | 7.07E-02 |
| 1.76E+06 | 0.00E+00 | 0.00E+00 | 0.00E+00 | 0.00E+00 | 0.00E+00 | 4.67E-02 | 2.39E-01 | 1.39E-01 | 8.38E-02 |
| 1.79E+06 | 0.00E+00 | 0.00E+00 | 0.00E+00 | 0.00E+00 | 0.00E+00 | 4.94E-02 | 2.40E-01 | 1.31E-01 | 6.95E-02 |
| 1.83E+06 | 0.00E+00 | 0.00E+00 | 0.00E+00 | 0.00E+00 | 0.00E+00 | 4.15E-02 | 2.24E-01 | 1.21E-01 | 6.19E-02 |
| 1.87E+06 | 0.00E+00 | 0.00E+00 | 0.00E+00 | 0.00E+00 | 0.00E+00 | 4.57E-02 | 2.11E-01 | 1.19E-01 | 7.21E-02 |
| 1.90E+06 | 0.00E+00 | 0.00E+00 | 0.00E+00 | 0.00E+00 | 0.00E+00 | 3.61E-02 | 2.01E-01 | 1.13E-01 | 7.46E-02 |
| 1.94E+06 | 0.00E+00 | 0.00E+00 | 0.00E+00 | 0.00E+00 | 0.00E+00 | 2.36E-02 | 1.97E-01 | 9.57E-02 | 5.61E-02 |
| 1.98E+06 | 0.00E+00 | 0.00E+00 | 0.00E+00 | 0.00E+00 | 0.00E+00 | 3.64E-02 | 1.90E-01 | 9.58E-02 | 6.21E-02 |
| 2.02E+06 | 0.00E+00 | 0.00E+00 | 0.00E+00 | 0.00E+00 | 0.00E+00 | 1.37E-02 | 1.78E-01 | 9.22E-02 | 7.78E-02 |
| 2.06E+06 | 0.00E+00 | 0.00E+00 | 0.00E+00 | 0.00E+00 | 0.00E+00 | 1.87E-02 | 1.70E-01 | 8.37E-02 | 5.21E-02 |
| 2.11E+06 | 0.00E+00 | 0.00E+00 | 0.00E+00 | 0.00E+00 | 0.00E+00 | 2.29E-02 | 1.67E-01 | 7.45E-02 | 6.54E-02 |
| 2.15E+06 | 0.00E+00 | 0.00E+00 | 0.00E+00 | 0.00E+00 | 0.00E+00 | 1.06E-02 | 1.61E-01 | 6.48E-02 | 6.46E-02 |
| 2.19E+06 | 0.00E+00 | 0.00E+00 | 0.00E+00 | 0.00E+00 | 0.00E+00 | 1.57E-02 | 1.42E-01 | 5.65E-02 | 4.77E-02 |
| 2.24E+06 | 0.00E+00 | 0.00E+00 | 0.00E+00 | 0.00E+00 | 0.00E+00 | 6.16E-03 | 1.43E-01 | 5.63E-02 | 4.93E-02 |
| 2.28E+06 | 0.00E+00 | 0.00E+00 | 0.00E+00 | 0.00E+00 | 0.00E+00 | 0.00E+00 | 1.39E-01 | 5.99E-02 | 4.62E-02 |
| 2.33E+06 | 0.00E+00 | 0.00E+00 | 0.00E+00 | 0.00E+00 | 0.00E+00 | 6.88E-03 | 1.28E-01 | 4.52E-02 | 2.75E-02 |
| 2.38E+06 | 0.00E+00 | 0.00E+00 | 0.00E+00 | 0.00E+00 | 0.00E+00 | 1.48E-02 | 1.17E-01 | 2.81E-02 | 6.26E-02 |
| 2.43E+06 | 0.00E+00 | 0.00E+00 | 0.00E+00 | 0.00E+00 | 0.00E+00 | 5.42E-03 | 1.07E-01 | 2.24E-02 | 5.17E-02 |
| 2.48E+06 | 0.00E+00 | 0.00E+00 | 0.00E+00 | 0.00E+00 | 0.00E+00 | 0.00E+00 | 9.90E-02 | 2.54E-02 | 2.10E-02 |
| 2.53E+06 | 0.00E+00 | 0.00E+00 | 0.00E+00 | 0.00E+00 | 0.00E+00 | 1.92E-02 | 9.77E-02 | 2.28E-02 | 3.97E-02 |
| 2.58E+06 | 0.00E+00 | 0.00E+00 | 0.00E+00 | 0.00E+00 | 0.00E+00 | 9.37E-03 | 9.49E-02 | 1.82E-02 | 3.41E-02 |
| 2.63E+06 | 0.00E+00 | 0.00E+00 | 0.00E+00 | 0.00E+00 | 0.00E+00 | 0.00E+00 | 8.09E-02 | 1.62E-02 | 4.13E-02 |
| 2.69E+06 | 0.00E+00 | 0.00E+00 | 0.00E+00 | 0.00E+00 | 0.00E+00 | 0.00E+00 | 7.53E-02 | 6.42E-03 | 1.75E-02 |
| 2.74E+06 | 0.00E+00 | 0.00E+00 | 0.00E+00 | 0.00E+00 | 0.00E+00 | 0.00E+00 | 6.31E-02 | 0.00E+00 | 2.56E-02 |
| 2.80E+06 | 0.00E+00 | 0.00E+00 | 0.00E+00 | 0.00E+00 | 0.00E+00 | 1.02E-03 | 5.69E-02 | 0.00E+00 | 1.80E-02 |
| 2.85E+06 | 0.00E+00 | 0.00E+00 | 0.00E+00 | 0.00E+00 | 0.00E+00 | 5.35E-04 | 5.03E-02 | 0.00E+00 | 2.43E-02 |
| 2.91E+06 | 0.00E+00 | 0.00E+00 | 0.00E+00 | 0.00E+00 | 0.00E+00 | 0.00E+00 | 4.87E-02 | 0.00E+00 | 2.42E-02 |
| 2.97E+06 | 0.00E+00 | 0.00E+00 | 0.00E+00 | 0.00E+00 | 0.00E+00 | 0.00E+00 | 4.05E-02 | 0.00E+00 | 2.27E-02 |
| 3.03E+06 | 0.00E+00 | 0.00E+00 | 0.00E+00 | 0.00E+00 | 0.00E+00 | 0.00E+00 | 3.83E-02 | 0.00E+00 | 2.66E-02 |
| 3.09E+06 | 0.00E+00 | 0.00E+00 | 0.00E+00 | 0.00E+00 | 0.00E+00 | 0.00E+00 | 2.83E-02 | 0.00E+00 | 2.30E-02 |
| 3.16E+06 | 0.00E+00 | 0.00E+00 | 0.00E+00 | 0.00E+00 | 0.00E+00 | 0.00E+00 | 2.11E-02 | 0.00E+00 | 1.78E-02 |
| 3.22E+06 | 0.00E+00 | 0.00E+00 | 0.00E+00 | 0.00E+00 | 0.00E+00 | 0.00E+00 | 1.29E-02 | 0.00E+00 | 3.52E-02 |
| 3.29E+06 | 0.00E+00 | 0.00E+00 | 0.00E+00 | 0.00E+00 | 0.00E+00 | 0.00E+00 | 5.83E-03 | 0.00E+00 | 1.50E-02 |
| 3.36E+06 | 0.00E+00 | 0.00E+00 | 0.00E+00 | 0.00E+00 | 0.00E+00 | 0.00E+00 | 4.12E-03 | 0.00E+00 | 1.62E-02 |
| 3.42E+06 | 0.00E+00 | 0.00E+00 | 0.00E+00 | 0.00E+00 | 0.00E+00 | 0.00E+00 | 0.00E+00 | 0.00E+00 | 1.57E-02 |
| 3.49E+06 | 0.00E+00 | 0.00E+00 | 0.00E+00 | 0.00E+00 | 0.00E+00 | 0.00E+00 | 0.00E+00 | 0.00E+00 | 1.84E-02 |
| 3.57E+06 | 0.00E+00 | 0.00E+00 | 0.00E+00 | 0.00E+00 | 0.00E+00 | 0.00E+00 | 0.00E+00 | 0.00E+00 | 1.11E-03 |
| 3.64E+06 | 0.00E+00 | 0.00E+00 | 0.00E+00 | 0.00E+00 | 0.00E+00 | 0.00E+00 | 0.00E+00 | 0.00E+00 | 1.57E-02 |
| 3.71E+06 | 0.00E+00 | 0.00E+00 | 0.00E+00 | 0.00E+00 | 0.00E+00 | 0.00E+00 | 0.00E+00 | 0.00E+00 | 9.43E-03 |
| 3.79E+06 | 0.00E+00 | 0.00E+00 | 0.00E+00 | 0.00E+00 | 0.00E+00 | 1.72E-03 | 0.00E+00 | 0.00E+00 | 2.63E-02 |
| 3.87E+06 | 0.00E+00 | 0.00E+00 | 0.00E+00 | 0.00E+00 | 0.00E+00 | 1.10E-03 | 0.00E+00 | 0.00E+00 | 5.27E-05 |
| 3.95E+06 | 0.00E+00 | 0.00E+00 | 0.00E+00 | 0.00E+00 | 0.00E+00 | 0.00E+00 | 0.00E+00 | 0.00E+00 | 1.17E-02 |
| 4.03E+06 | 0.00E+00 | 0.00E+00 | 0.00E+00 | 0.00E+00 | 0.00E+00 | 0.00E+00 | 0.00E+00 | 0.00E+00 | 1.70E-03 |
| 4.11E+06 | 0.00E+00 | 0.00E+00 | 0.00E+00 | 0.00E+00 | 0.00E+00 | 0.00E+00 | 0.00E+00 | 0.00E+00 | 1.06E-02 |
| 4.19E+06 | 0.00E+00 | 0.00E+00 | 0.00E+00 | 0.00E+00 | 0.00E+00 | 0.00E+00 | 0.00E+00 | 0.00E+00 | 6.53E-03 |
| 4.28E+06 | 0.00E+00 | 0.00E+00 | 0.00E+00 | 0.00E+00 | 0.00E+00 | 0.00E+00 | 0.00E+00 | 0.00E+00 | 1.24E-02 |
| 4.37E+06 | 0.00E+00 | 0.00E+00 | 0.00E+00 | 0.00E+00 | 0.00E+00 | 0.00E+00 | 0.00E+00 | 0.00E+00 | 1.34E-03 |
| 4.45E+06 | 0.00E+00 | 0.00E+00 | 0.00E+00 | 0.00E+00 | 0.00E+00 | 0.00E+00 | 0.00E+00 | 0.00E+00 | 1.48E-05 |
| 4.55E+06 | 0.00E+00 | 0.00E+00 | 0.00E+00 | 0.00E+00 | 0.00E+00 | 0.00E+00 | 0.00E+00 | 0.00E+00 | 9.47E-03 |
| 4.64E+06 | 0.00E+00 | 0.00E+00 | 0.00E+00 | 0.00E+00 | 0.00E+00 | 0.00E+00 | 0.00E+00 | 0.00E+00 | 9.05E-03 |
| 4.73E+06 | 0.00E+00 | 0.00E+00 | 0.00E+00 | 0.00E+00 | 0.00E+00 | 0.00E+00 | 0.00E+00 | 0.00E+00 | 1.73E-04 |
| 4.83E+06 | 0.00E+00 | 0.00E+00 | 0.00E+00 | 0.00E+00 | 0.00E+00 | 0.00E+00 | 0.00E+00 | 0.00E+00 | 3.69E-03 |
| 4.93E+06 | 0.00E+00 | 0.00E+00 | 0.00E+00 | 0.00E+00 | 0.00E+00 | 0.00E+00 | 0.00E+00 | 0.00E+00 | 1.42E-02 |
| 5.03E+06 | 0.00E+00 | 0.00E+00 | 0.00E+00 | 0.00E+00 | 0.00E+00 | 0.00E+00 | 0.00E+00 | 0.00E+00 | 5.27E-03 |
| 5.13E+06 | 0.00E+00 | 0.00E+00 | 0.00E+00 | 0.00E+00 | 0.00E+00 | 0.00E+00 | 0.00E+00 | 0.00E+00 | 1.43E-04 |
| 5.24E+06 | 0.00E+00 | 0.00E+00 | 0.00E+00 | 0.00E+00 | 0.00E+00 | 0.00E+00 | 0.00E+00 | 0.00E+00 | 7.23E-03 |
| 5.34E+06 | 0.00E+00 | 0.00E+00 | 0.00E+00 | 0.00E+00 | 0.00E+00 | 0.00E+00 | 0.00E+00 | 0.00E+00 | 3.48E-03 |
| 5.45E+06 | 0.00E+00 | 0.00E+00 | 0.00E+00 | 0.00E+00 | 0.00E+00 | 0.00E+00 | 0.00E+00 | 0.00E+00 | 9.67E-03 |
| 5.56E+06 | 0.00E+00 | 0.00E+00 | 0.00E+00 | 0.00E+00 | 0.00E+00 | 0.00E+00 | 0.00E+00 | 0.00E+00 | 8.79E-03 |
| 5.68E+06 | 0.00E+00 | 0.00E+00 | 0.00E+00 | 0.00E+00 | 0.00E+00 | 0.00E+00 | 0.00E+00 | 0.00E+00 | 1.11E-02 |
| 5.79E+06 | 0.00E+00 | 0.00E+00 | 0.00E+00 | 0.00E+00 | 0.00E+00 | 0.00E+00 | 0.00E+00 | 0.00E+00 | 4.76E-04 |
| 5.91E+06 | 0.00E+00 | 0.00E+00 | 0.00E+00 | 0.00E+00 | 0.00E+00 | 0.00E+00 | 0.00E+00 | 0.00E+00 | 1.07E-02 |
| 6.03E+06 | 0.00E+00 | 0.00E+00 | 0.00E+00 | 0.00E+00 | 0.00E+00 | 0.00E+00 | 0.00E+00 | 0.00E+00 | 8.43E-03 |
| 6.16E+06 | 0.00E+00 | 0.00E+00 | 0.00E+00 | 0.00E+00 | 0.00E+00 | 0.00E+00 | 0.00E+00 | 0.00E+00 | 1.34E-02 |
| 6.28E+06 | 0.00E+00 | 0.00E+00 | 0.00E+00 | 0.00E+00 | 0.00E+00 | 0.00E+00 | 0.00E+00 | 0.00E+00 | 5.26E-03 |
| 6.41E+06 | 0.00E+00 | 0.00E+00 | 0.00E+00 | 0.00E+00 | 0.00E+00 | 8.31E-03 | 0.00E+00 | 0.00E+00 | 1.21E-02 |
| 6.54E+06 | 0.00E+00 | 0.00E+00 | 0.00E+00 | 0.00E+00 | 0.00E+00 | 7.32E-03 | 0.00E+00 | 0.00E+00 | 7.12E-04 |
| 6.68E+06 | 0.00E+00 | 0.00E+00 | 0.00E+00 | 0.00E+00 | 0.00E+00 | 0.00E+00 | 0.00E+00 | 0.00E+00 | 0.00E+00 |
| 6.81E+06 | 0.00E+00 | 0.00E+00 | 0.00E+00 | 0.00E+00 | 0.00E+00 | 0.00E+00 | 0.00E+00 | 0.00E+00 | 0.00E+00 |
| 6.95E+06 | 0.00E+00 | 0.00E+00 | 0.00E+00 | 0.00E+00 | 0.00E+00 | 0.00E+00 | 0.00E+00 | 0.00E+00 | 0.00E+00 |
| 7.09E+06 | 0.00E+00 | 0.00E+00 | 0.00E+00 | 0.00E+00 | 0.00E+00 | 0.00E+00 | 0.00E+00 | 0.00E+00 | 9.09E-03 |
| 7.24E+06 | 0.00E+00 | 0.00E+00 | 0.00E+00 | 0.00E+00 | 0.00E+00 | 0.00E+00 | 0.00E+00 | 0.00E+00 | 6.68E-04 |
| 7.39E+06 | 0.00E+00 | 0.00E+00 | 0.00E+00 | 0.00E+00 | 0.00E+00 | 0.00E+00 | 0.00E+00 | 0.00E+00 | 0.00E+00 |
| 7.54E+06 | 0.00E+00 | 0.00E+00 | 0.00E+00 | 0.00E+00 | 0.00E+00 | 0.00E+00 | 0.00E+00 | 0.00E+00 | 1.63E-02 |
| 7.69E+06 | 0.00E+00 | 0.00E+00 | 0.00E+00 | 0.00E+00 | 0.00E+00 | 0.00E+00 | 0.00E+00 | 0.00E+00 | 1.98E-02 |
| 7.85E+06 | 1.64E-04 | 0.00E+00 | 0.00E+00 | 0.00E+00 | 0.00E+00 | 0.00E+00 | 0.00E+00 | 0.00E+00 | 7.41E-03 |
| 8.01E+06 | 6.49E-04 | 0.00E+00 | 0.00E+00 | 0.00E+00 | 0.00E+00 | 0.00E+00 | 0.00E+00 | 0.00E+00 | 5.10E-04 |
| 8.17E+06 | 0.00E+00 | 0.00E+00 | 0.00E+00 | 0.00E+00 | 0.00E+00 | 0.00E+00 | 0.00E+00 | 0.00E+00 | 0.00E+00 |
| 8.34E+06 | 0.00E+00 | 0.00E+00 | 0.00E+00 | 0.00E+00 | 0.00E+00 | 0.00E+00 | 0.00E+00 | 0.00E+00 | 0.00E+00 |
| 8.51E+06 | 0.00E+00 | 0.00E+00 | 0.00E+00 | 0.00E+00 | 0.00E+00 | 3.86E-03 | 0.00E+00 | 0.00E+00 | 0.00E+00 |
| 8.68E+06 | 2.28E-04 | 0.00E+00 | 0.00E+00 | 0.00E+00 | 0.00E+00 | 4.03E-03 | 0.00E+00 | 0.00E+00 | 5.93E-03 |
| 8.86E+06 | 9.58E-04 | 0.00E+00 | 0.00E+00 | 0.00E+00 | 0.00E+00 | 0.00E+00 | 0.00E+00 | 0.00E+00 | 1.84E-02 |
| 9.04E+06 | 0.00E+00 | 0.00E+00 | 2.65E-03 | 0.00E+00 | 0.00E+00 | 0.00E+00 | 0.00E+00 | 0.00E+00 | 1.21E-02 |
| 9.23E+06 | 0.00E+00 | 0.00E+00 | 5.10E-04 | 0.00E+00 | 0.00E+00 | 0.00E+00 | 0.00E+00 | 0.00E+00 | 9.21E-03 |
| 9.42E+06 | 0.00E+00 | 0.00E+00 | 0.00E+00 | 0.00E+00 | 0.00E+00 | 0.00E+00 | 0.00E+00 | 0.00E+00 | 3.13E-03 |
| 9.61E+06 | 0.00E+00 | 0.00E+00 | 0.00E+00 | 0.00E+00 | 0.00E+00 | 6.38E-03 | 0.00E+00 | 0.00E+00 | 1.32E-02 |
| 9.80E+06 | 0.00E+00 | 0.00E+00 | 0.00E+00 | 0.00E+00 | 0.00E+00 | 7.17E-03 | 0.00E+00 | 0.00E+00 | 5.38E-03 |
| 1.00E+07 | 0.00E+00 | 0.00E+00 | 0.00E+00 | 0.00E+00 | 1.36E-02 | 0.00E+00 | 0.00E+00 | 0.00E+00 | 4.61E-04 |
| 1.02E+07 | 1.08E-04 | 0.00E+00 | 0.00E+00 | 0.00E+00 | 2.15E-03 | 0.00E+00 | 0.00E+00 | 0.00E+00 | 0.00E+00 |
| 1.04E+07 | 5.00E-04 | 0.00E+00 | 0.00E+00 | 0.00E+00 | 6.68E-03 | 0.00E+00 | 0.00E+00 | 0.00E+00 | 2.03E-02 |
| 1.06E+07 | 0.00E+00 | 0.00E+00 | 0.00E+00 | 0.00E+00 | 1.10E-03 | 0.00E+00 | 0.00E+00 | 0.00E+00 | 1.29E-02 |
| 1.08E+07 | 1.02E-04 | 0.00E+00 | 0.00E+00 | 0.00E+00 | 0.00E+00 | 6.82E-03 | 0.00E+00 | 0.00E+00 | 2.32E-02 |
| 1.11E+07 | 4.94E-04 | 0.00E+00 | 0.00E+00 | 0.00E+00 | 0.00E+00 | 8.25E-03 | 0.00E+00 | 0.00E+00 | 2.21E-02 |
| 1.13E+07 | 2.04E-04 | 0.00E+00 | 0.00E+00 | 0.00E+00 | 0.00E+00 | 0.00E+00 | 0.00E+00 | 0.00E+00 | 1.13E-02 |
| 1.15E+07 | 1.23E-03 | 0.00E+00 | 0.00E+00 | 0.00E+00 | 0.00E+00 | 0.00E+00 | 0.00E+00 | 0.00E+00 | 1.22E-03 |
| 1.18E+07 | 1.08E-03 | 0.00E+00 | 0.00E+00 | 0.00E+00 | 0.00E+00 | 2.96E-04 | 0.00E+00 | 0.00E+00 | 4.05E-03 |
| 1.20E+07 | 0.00E+00 | 0.00E+00 | 4.18E-03 | 0.00E+00 | 0.00E+00 | 3.76E-04 | 0.00E+00 | 0.00E+00 | 5.91E-04 |
| 1.22E+07 | 1.30E-04 | 0.00E+00 | 1.02E-03 | 0.00E+00 | 6.78E-04 | 0.00E+00 | 0.00E+00 | 0.00E+00 | 1.51E-03 |
| 1.25E+07 | 6.79E-04 | 0.00E+00 | 6.96E-06 | 0.00E+00 | 1.30E-04 | 0.00E+00 | 0.00E+00 | 0.00E+00 | 7.30E-04 |
| 1.28E+07 | 7.63E-05 | 0.00E+00 | 1.75E-06 | 0.00E+00 | 0.00E+00 | 1.79E-02 | 0.00E+00 | 0.00E+00 | 1.29E-02 |
| 1.30E+07 | 6.80E-04 | 0.00E+00 | 0.00E+00 | 0.00E+00 | 0.00E+00 | 2.67E-02 | 0.00E+00 | 0.00E+00 | 1.44E-02 |
| 1.33E+07 | 1.85E-03 | 0.00E+00 | 0.00E+00 | 0.00E+00 | 1.34E-02 | 3.78E-03 | 7.03E-04 | 0.00E+00 | 1.69E-02 |
| 1.36E+07 | 2.99E-03 | 0.00E+00 | 0.00E+00 | 0.00E+00 | 2.74E-03 | 0.00E+00 | 2.34E-03 | 0.00E+00 | 6.62E-03 |
| 1.38E+07 | 5.51E-03 | 0.00E+00 | 0.00E+00 | 0.00E+00 | 0.00E+00 | 0.00E+00 | 0.00E+00 | 0.00E+00 | 1.56E-02 |
| 1.41E+07 | 4.54E-03 | 4.74E-03 | 2.23E-03 | 0.00E+00 | 0.00E+00 | 4.41E-03 | 0.00E+00 | 0.00E+00 | 3.00E-02 |
| 1.44E+07 | 2.74E-03 | 6.55E-04 | 4.09E-03 | 0.00E+00 | 1.10E-03 | 9.28E-03 | 0.00E+00 | 0.00E+00 | 1.10E-02 |
| 1.47E+07 | 0.00E+00 | 0.00E+00 | 9.70E-04 | 0.00E+00 | 2.41E-04 | 4.33E-03 | 0.00E+00 | 0.00E+00 | 1.11E-03 |
| 1.50E+07 | 8.69E-05 | 0.00E+00 | 0.00E+00 | 0.00E+00 | 0.00E+00 | 0.00E+00 | 0.00E+00 | 0.00E+00 | 7.41E-04 |
| 1.53E+07 | 1.61E-03 | 0.00E+00 | 2.51E-03 | 0.00E+00 | 0.00E+00 | 1.57E-04 | 2.16E-04 | 0.00E+00 | 1.40E-03 |
| 1.56E+07 | 8.26E-03 | 0.00E+00 | 5.08E-03 | 0.00E+00 | 4.34E-03 | 6.42E-03 | 7.73E-04 | 0.00E+00 | 4.03E-03 |
| 1.59E+07 | 9.98E-03 | 0.00E+00 | 6.25E-03 | 0.00E+00 | 1.02E-03 | 9.37E-03 | 0.00E+00 | 0.00E+00 | 1.50E-02 |
| 1.63E+07 | 8.47E-04 | 0.00E+00 | 1.49E-03 | 0.00E+00 | 0.00E+00 | 5.49E-03 | 5.73E-04 | 0.00E+00 | 1.16E-02 |
| 1.66E+07 | 5.88E-03 | 0.00E+00 | 9.67E-04 | 3.56E-04 | 3.72E-04 | 8.53E-03 | 2.12E-03 | 0.00E+00 | 1.74E-03 |
| 1.69E+07 | 2.83E-03 | 0.00E+00 | 2.98E-04 | 3.63E-04 | 9.11E-05 | 0.00E+00 | 0.00E+00 | 0.00E+00 | 6.33E-03 |
| 1.73E+07 | 1.64E-03 | 0.00E+00 | 1.61E-03 | 0.00E+00 | 5.19E-03 | 0.00E+00 | 1.53E-03 | 1.17E-03 | 1.16E-02 |

Raw data Fig. 6

| name | runtime | Fouling mass | runtime | Fouling mass | runtime | Fouling mass |
| --- | --- | --- | --- | --- | --- | --- |
| unit | min | mg/g | min | mg/g | min | mg/g |
| comment | - | 5 °C | - | 20 °C |  | 40°C |
|  | 30 |  | 60 | 51.5 | 30 | 93 |
|  | 30 |  | 30 | 16 | 30 | 78 |
|  | 40 |  | 60 | 24.2 | 45 | 145 |
|  | 40 |  | 120 | 98.5 | 45 | 133 |
|  | 50 |  | 90 | 88.9 | 60 | 173 |
|  | 60 | 19.5569 | 30 | 19.9 | 60 | 149 |
|  | 60 | 14.10725 | 30 | 3.99 | 75 | 179 |
|  | 75 | 16.5422 | 90 | 87.6 | 75 | 201 |
|  | 75 | 19.50279 | 30 | 28.2 | 90 | 256 |
|  | 90 | 14.09179 | 45 | 20.9 | 90 | 217 |
|  | 90 | 12.10518 | 45 | 47.7 | 120 | 303 |
|  | 120 |  | 60 | 65.8 | 120 | 310 |
|  | 120 | 16.88232 | 60 | 48.9 |  |  |
|  | 120 | 19.28635 | 30 | 18.6 |  |  |
|  | 150 | 17.28428 | 45 | 27.2 |  |  |
|  | 150 | 26.14286 | 20 | 7.85 |  |  |
|  | 180 | 17.83311 | 20 | 9.74 |  |  |
|  | 180 | 20.3299 | 30 | 12.41438 |  |  |
|  |  |  | 30 | 18.98488 |  |  |
|  |  |  | 40 | 20.52315 |  |  |
|  |  |  | 40 | 21.23431 |  |  |
|  |  |  | 50 | 21.57443 |  |  |
|  |  |  | 60 | 40.5052 |  |  |
|  |  |  | 60 | 30.6108 |  |  |
|  |  |  | 75 | 42.15942 |  |  |
|  |  |  | 75 | 42.40678 |  |  |
|  |  |  | 90 | 75.93952 |  |  |
|  |  |  | 90 | 71.4252 |  |  |
|  |  |  | 120 | 103.65157 |  |  |
|  |  |  | 120 | 113.26769 |  |  |
|  |  |  | 120 | 116.81576 |  |  |
|  |  |  | 150 | 175.58695 |  |  |
|  |  |  | 150 | 156.58661 |  |  |
|  |  |  | 180 | 186.48625 |  |  |
|  |  |  | 180 | 186.16932 |  |  |

Raw data Fig. 7

| Name | mixing element | fouling mass | Measurement error | fouling mass | Measurement error |
| --- | --- | --- | --- | --- | --- |
| unit | - | mg/g | mg/g | mg/g | mg/g |
| comment |  | local, 20 °C |  | integral, 20 °C |  |
|  | 1 | 203.16109 | 23.94982 | 188.53156 | 21.21738 |
|  | 2 | 162.16216 | 32.2428 | 188.53156 | 21.21738 |
|  | 3 | 154.35435 | 16.58354 | 188.53156 | 21.21738 |
|  | 4 | 173.21321 | 22.57044 | 188.53156 | 21.21738 |
|  | 5 | 191.23123 | 29.66164 | 188.53156 | 21.21738 |
|  | 6 | 214.65465 | 25.89863 | 188.53156 | 21.21738 |
|  | 7 | 220.94421 | 32.64783 | 188.53156 | 21.21738 |

Raw data Fig. 8

| name | runtime | Fouling mass | runtime | Fouling mass |
| --- | --- | --- | --- | --- |
| unit | min | mg/g | min | mg/g |
| comment |  | Mixing element 7 |  | Reactor average |
|  | 60 | 86.7 | 60 | 51.5 |
|  | 30 | 62.7 | 30 | 16 |
|  | 60 | 33.5 | 60 | 24.2 |
|  | 120 | 186.8 | 120 | 98.5 |
|  | 90 | 170.8 | 90 | 88.9 |
|  | 60 | 165.7 | 30 | 19.9 |
|  | 30 | 48.1 | 30 | 3.99 |
|  | 30 | 34.3 | 90 | 87.6 |
|  | 90 | 163.9 | 30 | 28.2 |
|  | 30 | 25.8 | 45 | 20.9 |
|  | 45 | 77.3 | 45 | 47.7 |
|  | 45 | 137.3 | 60 | 65.8 |
|  | 60 | 149.3 | 60 | 48.9 |
|  | 60 | 103.9 | 30 | 18.6 |
|  | 30 | 55.8 | 45 | 27.2 |
|  | 45 | 56.7 | 20 | 7.85 |
|  | 20 | 10.3 | 20 | 9.74 |
|  | 20 | 14.6 | 30 | 12.41438 |
|  | 150 | 188.4 | 30 | 18.98488 |
|  | 150 | 165.3 | 40 | 20.52315 |
|  | 180 | 192.3 | 40 | 21.23431 |
|  | 180 | 182 | 50 | 21.57443 |
|  | 120 | 174.4 | 60 | 40.5052 |
|  |  |  | 60 | 30.6108 |
|  |  |  | 75 | 42.15942 |
|  |  |  | 75 | 42.40678 |
|  |  |  | 90 | 75.93952 |
|  |  |  | 90 | 71.4252 |
|  |  |  | 120 | 103.65157 |
|  |  |  | 120 | 113.26769 |
|  |  |  | 120 | 116.81576 |
|  |  |  | 150 | 175.58695 |
|  |  |  | 150 | 156.58661 |
|  |  |  | 180 | 186.48625 |
|  |  |  | 180 | 186.16932 |

Raw data Fig. 9

| name | Decrease in reaction volume | Decrease in conversion |
| --- | --- | --- |
| unit | - | - |
|  | 0.29052 | 0.32967 |
|  | 0.14072 | 0.14706 |
|  | 0.48191 | 0.52326 |
|  | 0.4187 | 0.5 |
|  | 0.44386 | 0.38824 |
|  | 0.10827 | 0.07692 |
|  | 0.08672 | 0.14737 |
|  | 0.42438 | 0.39726 |
|  | 0.09831 | 0.0274 |
|  | 0.23298 | 0.10588 |
|  | 0.2748 | 0.20732 |
|  | 0.36789 | 0.32558 |
|  | 0.30287 | 0.3 |
|  | 0.11343 | 0.04819 |
|  | 0.21978 | 0.275 |
|  | 0.03933 | 0.07407 |

Raw data Fig. 10

| name | Fouling mass | Fouling mass | Fouling mass | Fouling mass |
| --- | --- | --- | --- | --- |
| unit | mg/g | g | mg/g | g |
| comment | Average reactor (a) | Measurement cell | Static mixer 7 (b) | Measurement cell |
|  | 51.5 | 1.008 | 34.2 | 0.671 |
|  | 24.2 | 0.79 | 33.8 | 0.788 |
|  | 98.5 | 1.11412 | 47.97 | 0.859 |
|  | 88.9 | 1.113 | 56.2 | 0.623 |
|  | 19.9 | 0.86 | 86.4 | 1.007 |
|  | 3.99 | 0.672 | 103.9 | 0.935 |
|  | 28.2 | 0.426 | 105.7 | 0.934 |
|  | 20.9 | 1 | 137.7 | 0.979 |
|  | 47.7 | 0.98 | 148.3 | 1.039 |
|  | 65.8 | 1.0339 | 151.5 | 1.029 |
|  | 48.9 | 0.936 | 166.1 | 1.185 |
|  | 18.6 | 0.623 | 170.7 | 1.112 |
|  | 27.2 | 0.999 | 187.2 | 1.113 |
|  | 7.85 | 0.141 |  |  |
|  | 9.74 | 0.206 |  |  |

Raw data Fig. 12

| name | ΔASV | Fouling mass |
| --- | --- | --- |
| unit | m/s | g |
| comment |  | Measurement cell |
|  | 125 | 1.008 |
|  | 6 | 0.152 |
|  | 120 | 1.1142 |
|  | 170 | 1.113 |
|  | 150 | 1.185 |
|  | 120 | 0.86 |
|  | 78 | 0.672 |
|  | 130 | 1.0003 |
|  | 120 | 0.98 |
|  | 155 | 1.0339 |
|  | 125 | 0.936 |
|  | 85 | 0.625 |
|  | 140 | 0.999 |
|  | 20 | 0.141 |
|  | 35 | 0.206 |

Raw data Fig. 11+13

| Fig. 11 | | | Fig. 13 | |
| --- | --- | --- | --- | --- |
| time | sound velocity | fouling mass | runtime | ASV |
| min | m/s | mg/g | min | m/s |
|  |  | static mixer 7 |  |  |
| 0 | 1309.2 | 0 | -20 | 1217.2 |
| 0.32032 | 1311.2 |  | -19.5 | 1217 |
| 0.63738 | 1313.5 |  | -19 | 1218.7 |
| 0.95007 | 1312.9 |  | -18.5 | 1220.8 |
| 1.2631 | 1316.7 |  | -18 | 1219.5 |
| 1.5744 | 1314.3 |  | -17.5 | 1218.6 |
| 1.8826 | 1310.8 |  | -17 | 1218.4 |
| 2.1933 | 1311.4 |  | -16.5 | 1218.2 |
| 2.5084 | 1308 |  | -16 | 1218.7 |
| 2.8211 | 1312 |  | -15.5 | 1218.8 |
| 3.1326 | 1312.4 |  | -15 | 1219.6 |
| 3.4405 | 1317.5 |  | -14.5 | 1217 |
| 3.7518 | 1318.7 |  | -14 | 1218.7 |
| 4.0672 | 1323.1 |  | -13.5 | 1218.4 |
| 4.3958 | 1325.1 |  | -13 | 1218.2 |
| 4.7612 | 1326.6 |  | -12.5 | 1218.7 |
| 5.1139 | 1321.5 |  | -12 | 1218.8 |
| 5.4635 | 1327.6 |  | -11.5 | 1219.6 |
| 5.7874 | 1329.3 |  |  |  |
| 6.1005 | 1333.3 |  | 0.167 | 1369.013333 |
| 6.4111 | 1331.3 |  | 0.469 | 1370.126667 |
| 6.7252 | 1327.1 |  | 0.776 | 1369.26 |
| 7.0374 | 1328.3 |  | 1.089 | 1367.926667 |
| 7.3469 | 1326 |  | 1.391 | 1366.613333 |
| 7.6573 | 1321.6 |  | 1.694 | 1364.94 |
| 7.9675 | 1321.6 |  | 2.008 | 1364.36 |
| 8.2833 | 1320.4 |  | 2.316 | 1360.6 |
| 8.596 | 1316.4 |  | 2.62 | 1364.08 |
| 8.9246 | 1313.6 |  | 2.925 | 1366.473333 |
| 9.2374 | 1313.5 |  | 3.233 | 1366.5 |
| 9.5502 | 1314.6 |  | 3.536 | 1366.34 |
| 9.8805 | 1314.3 |  | 3.842 | 1363.16 |
| 10.243 | 1315.7 |  | 4.148 | 1362.933333 |
| 10.614 | 1313 |  | 4.461 | 1366.106667 |
| 10.961 | 1312.8 |  | 4.759 | 1367.193333 |
| 11.31 | 1309.2 |  | 5.066 | 1366.28 |
| 11.635 | 1308.1 |  | 5.375 | 1368.386667 |
| 11.949 | 1305.9 |  | 5.676 | 1368.013333 |
| 12.262 | 1307.3 |  | 5.983 | 1366.533333 |
| 12.591 | 1301.7 |  | 6.292 | 1367.873333 |
| 12.919 | 1305.8 |  | 6.592 | 1365.64 |
| 13.234 | 1299.7 |  | 6.898 | 1365.666667 |
| 13.561 | 1307 |  | 7.197 | 1361.293333 |
| 13.887 | 1302.1 |  | 7.508 | 1359.833333 |
| 14.206 | 1295.2 |  | 7.839 | 1360.88 |
| 14.53 | 1292.6 |  | 8.156 | 1357.906667 |
| 14.843 | 1298.3 |  | 8.477 | 1358.466667 |
| 15.16 | 1299.2 |  | 8.808 | 1358.933333 |
| 15.485 | 1297 |  | 9.156 | 1354.813333 |
| 15.813 | 1296 |  | 9.486 | 1354.646667 |
| 16.127 | 1292.8 |  | 9.8 | 1351.26 |
| 16.457 | 1288.8 |  | 10.108 | 1348.253333 |
| 16.784 | 1288.5 |  | 10.412 | 1346.293333 |
| 17.117 | 1289.4 |  | 10.717 | 1343.346667 |
| 17.449 | 1289.3 |  | 11.024 | 1341.186667 |
| 17.768 | 1286.7 |  | 11.328 | 1342.513333 |
| 18.092 | 1285.7 |  | 11.633 | 1341.913333 |
| 18.419 | 1292.5 |  | 11.933 | 1344.32 |
| 18.735 | 1286.5 |  | 12.235 | 1343.726667 |
| 19.059 | 1289.2 |  | 12.533 | 1343.406667 |
| 19.375 | 1284.5 |  | 12.841 | 1344.573333 |
| 19.701 | 1287.1 | 10.3 | 13.149 | 1344.12 |
| 20.019 | 1283.6 | 14.6 | 13.446 | 1342.546667 |
| 20.343 | 1283.8 |  | 13.748 | 1345.24 |
| 20.659 | 1283.9 |  | 14.05 | 1345.16 |
| 20.985 | 1284.5 |  | 14.358 | 1346.38 |
| 21.3 | 1284.3 |  | 14.659 | 1346.193333 |
| 21.627 | 1286.3 |  | 14.964 | 1346.08 |
| 21.942 | 1290.6 |  | 15.262 | 1346.84 |
| 22.268 | 1289.5 |  | 15.572 | 1345.22 |
| 22.595 | 1288.6 |  | 15.869 | 1344.66 |
| 22.913 | 1286.9 |  | 16.174 | 1345.533333 |
| 23.239 | 1285.9 |  | 16.471 | 1344.68 |
| 23.552 | 1285.4 |  | 16.781 | 1345.553333 |
| 23.867 | 1286.4 |  | 17.079 | 1346.8 |
| 24.193 | 1285.5 |  | 17.379 | 1347.553333 |
| 24.509 | 1287.5 |  | 17.683 | 1348.36 |
| 24.82 | 1288.5 |  | 17.984 | 1348.793333 |
| 25.131 | 1286.8 |  | 18.286 | 1348.686667 |
| 25.443 | 1290.4 |  | 18.6 | 1348.933333 |
| 25.756 | 1287.4 |  | 18.906 | 1347.993333 |
| 26.067 | 1291.4 |  | 19.206 | 1346.906667 |
| 26.379 | 1287.7 |  | 19.519 | 1347.74 |
| 26.687 | 1293.3 |  | 19.825 | 1347.78 |
| 27.001 | 1297.3 |  | 20.124 | 1349.26 |
| 27.311 | 1295.9 |  | 20.433 | 1350.206667 |
| 27.625 | 1294.1 |  | 20.734 | 1350.413333 |
| 27.937 | 1292.7 |  | 21.034 | 1350.96 |
| 28.247 | 1299.4 |  | 21.333 | 1349.5 |
| 28.556 | 1298.7 |  | 21.64 | 1347.966667 |
| 28.869 | 1301.5 |  | 21.935 | 1348.013333 |
| 29.183 | 1298.6 |  | 22.23 | 1347.486667 |
| 29.502 | 1302.9 |  | 22.525 | 1348.026667 |
| 29.823 | 1298.8 | 55.8 | 22.831 | 1347.88 |
| 30.137 | 1305.2 | 25.8 | 23.129 | 1348.473333 |
| 30.465 | 1298.3 | 34.3 | 23.422 | 1348.593333 |
| 30.78 | 1308.1 | 48.1 | 23.717 | 1350.126667 |
| 31.088 | 1306.4 | 62.7 | 24.024 | 1349.78 |
| 31.399 | 1312.7 |  | 24.321 | 1350.106667 |
| 31.711 | 1306.6 |  | 24.628 | 1350.093333 |
| 32.025 | 1305.6 |  | 24.926 | 1350.473333 |
| 32.338 | 1307.6 |  | 25.231 | 1350.333333 |
| 32.648 | 1304.9 |  | 25.527 | 1350.326667 |
| 32.976 | 1311.3 |  | 25.824 | 1352.006667 |
| 33.287 | 1309.6 |  | 26.124 | 1354.586667 |
| 33.6 | 1307.8 |  | 26.425 | 1355.313333 |
| 33.911 | 1310.1 |  | 26.722 | 1356.926667 |
| 34.225 | 1313 |  | 27.016 | 1356.246667 |
| 34.538 | 1314.2 |  | 27.315 | 1356.44 |
| 34.846 | 1308.9 |  | 27.617 | 1356.393333 |
| 35.158 | 1318.3 |  | 27.914 | 1358.106667 |
| 35.472 | 1316.1 |  | 28.223 | 1359.773333 |
| 35.799 | 1319.6 |  | 28.52 | 1362.84 |
| 36.112 | 1314.1 |  | 28.826 | 1364.38 |
| 36.432 | 1317.2 |  | 29.122 | 1365.173333 |
| 36.753 | 1318.5 |  | 29.418 | 1367.16 |
| 37.082 | 1323.6 |  | 29.723 | 1369.193333 |
| 37.403 | 1324.7 |  | 30.036 | 1371.533333 |
| 37.724 | 1323.4 |  | 30.333 | 1370.946667 |
| 38.04 | 1324.9 |  | 30.628 | 1367.193333 |
| 38.365 | 1327.9 |  | 30.933 | 1362.66 |
| 38.678 | 1329.4 |  | 31.229 | 1353.88 |
| 39.006 | 1332.4 |  | 31.525 | 1346.173333 |
| 39.328 | 1329.2 |  | 31.825 | 1338.946667 |
| 39.649 | 1330 |  | 32.127 | 1332.226667 |
| 39.972 | 1334.4 |  | 32.422 | 1324.433333 |
| 40.291 | 1336.2 |  | 32.718 | 1318.9 |
| 40.608 | 1330.3 |  | 33.022 | 1316.273333 |
| 40.93 | 1333.6 |  | 33.319 | 1317.253333 |
| 41.248 | 1332.3 |  | 33.616 | 1317.586667 |
| 41.574 | 1335.4 |  | 33.908 | 1315.16 |
| 41.886 | 1335.4 |  | 34.204 | 1313.066667 |
| 42.2 | 1334.8 |  | 34.509 | 1311.153333 |
| 42.513 | 1339.1 |  | 34.804 | 1311.54 |
| 42.823 | 1338.7 |  | 35.1 | 1313.86 |
| 43.142 | 1333.3 |  | 35.4 | 1317.053333 |
| 43.462 | 1332 |  | 35.702 | 1323.753333 |
| 43.777 | 1339.1 |  | 35.998 | 1330.153333 |
| 44.104 | 1338.3 |  | 36.299 | 1335.553333 |
| 44.433 | 1340.4 |  | 36.6 | 1340.066667 |
| 44.759 | 1340.2 | 77.3 | 36.897 | 1344.02 |
| 45.08 | 1341.9 | 137.3 | 37.191 | 1345.013333 |
| 45.409 | 1345.2 | 56.7 | 37.485 | 1342.273333 |
| 45.722 | 1346.6 |  | 37.778 | 1336.74 |
| 46.032 | 1350.1 |  | 38.082 | 1332.1 |
| 46.343 | 1349.9 |  | 38.379 | 1328.226667 |
| 46.67 | 1350.8 |  | 38.676 | 1323.986667 |
| 46.982 | 1352.7 |  | 38.981 | 1319.713333 |
| 47.294 | 1350.6 |  | 39.278 | 1315.433333 |
| 47.608 | 1351.5 |  | 39.575 | 1311.24 |
| 47.921 | 1356.5 |  | 39.882 | 1307.333333 |
| 48.249 | 1353.6 |  | 40.176 | 1303.586667 |
| 48.563 | 1357.5 |  | 40.473 | 1300.246667 |
| 48.869 | 1358.8 |  | 40.781 | 1296.94 |
| 49.181 | 1353.9 |  | 41.078 | 1293.546667 |
| 49.495 | 1359.7 |  | 41.377 | 1290.246667 |
| 49.808 | 1353.7 |  | 41.679 | 1286.92 |
| 50.121 | 1356.1 |  | 41.983 | 1283.753333 |
| 50.429 | 1352.9 |  | 42.283 | 1280.573333 |
| 50.742 | 1359.2 |  | 42.577 | 1277.713333 |
| 51.051 | 1359 |  | 42.871 | 1275.033333 |
| 51.367 | 1360.4 |  | 43.167 | 1272.353333 |
| 51.679 | 1359.2 |  | 43.475 | 1269.66 |
| 52.006 | 1365.7 |  | 43.772 | 1266.98 |
| 52.321 | 1360.9 |  | 44.075 | 1264.3 |
| 52.65 | 1362.5 |  | 44.378 | 1261.3 |
| 52.963 | 1362.4 |  | 44.683 | 1258.74 |
| 53.284 | 1364.1 |  | 44.979 | 1256.133333 |
| 53.605 | 1366.1 |  | 45.282 | 1253.666667 |
| 53.932 | 1363.9 |  | 45.585 | 1251.286667 |
| 54.246 | 1363.8 |  | 45.881 | 1249.006667 |
| 54.559 | 1371.3 |  | 46.193 | 1246.933333 |
| 54.867 | 1372.7 |  | 46.517 | 1245.026667 |
| 55.176 | 1371.2 |  | 46.868 | 1243.393333 |
| 55.491 | 1367 |  | 47.18 | 1241.72 |
| 55.805 | 1373.9 |  | 47.49 | 1240.026667 |
| 56.126 | 1371.1 |  | 47.787 | 1238.426667 |
| 56.442 | 1369.1 |  | 48.087 | 1236.88 |
| 56.755 | 1371.2 |  | 48.392 | 1235.406667 |
| 57.067 | 1368.2 |  | 48.7 | 1234.02 |
| 57.379 | 1368.8 |  | 48.998 | 1233.246667 |
| 57.693 | 1372.3 |  | 49.307 | 1231.873333 |
| 58.004 | 1370 |  | 49.604 | 1230.56 |
| 58.313 | 1370.2 |  | 49.908 | 1229.353333 |
| 58.625 | 1367.6 |  | 50.205 | 1228.206667 |
| 58.936 | 1366.3 |  | 50.515 | 1227.113333 |
| 59.251 | 1372.8 |  | 50.813 | 1226.053333 |
| 59.563 | 1370.1 | 86.7 | 51.112 | 1224.84 |
| 59.872 | 1372.6 | 33.5 | 51.417 | 1223.66 |
| 60.182 | 1377.6 | 165.7 | 51.711 | 1222.526667 |
| 60.498 | 1375.2 | 149.3 | 52.01 | 1221.5 |
| 60.808 | 1379.3 | 103.9 | 52.313 | 1220.66 |
| 61.133 | 1378.3 |  | 52.623 | 1220 |
| 61.451 | 1378.9 |  | 52.921 | 1219.5 |
| 61.764 | 1377.3 |  | 53.226 | 1219.14 |
| 62.09 | 1381 |  | 53.525 | 1218.646667 |
| 62.405 | 1386.2 |  | 53.833 | 1218.533333 |
| 62.715 | 1379.1 |  | 54.129 | 1218.42 |
| 63.035 | 1382.6 |  | 54.435 | 1218.32 |
| 63.355 | 1381.3 |  | 54.737 | 1218.393333 |
| 63.666 | 1377.6 |  | 55.029 | 1218.62 |
| 63.978 | 1378.7 |  | 55.329 | 1218.801786 |
| 64.292 | 1375.2 |  | 55.634 | 1218.983571 |
| 64.756 | 1377 |  | 55.934 | 1219.165357 |
| 65.229 | 1375.3 |  | 56.235 | 1219.347143 |
| 65.6 | 1380.4 |  | 56.549 | 1219.528929 |
| 65.98 | 1378.6 |  | 56.845 | 1219.710714 |
| 66.308 | 1380.9 |  | 57.149 | 1219.8925 |
| 66.621 | 1380.9 |  |  |  |
| 66.929 | 1380.1 |  |  |  |
| 67.241 | 1383.3 |  |  |  |
| 67.553 | 1383.2 |  |  |  |
| 67.869 | 1381.7 |  |  |  |
| 68.181 | 1387.1 |  |  |  |
| 68.492 | 1386.5 |  |  |  |
| 68.801 | 1388.2 |  |  |  |
| 69.112 | 1386.6 |  |  |  |
| 69.425 | 1389.2 |  |  |  |
| 69.738 | 1388.1 |  |  |  |
| 70.047 | 1388 |  |  |  |
| 70.359 | 1385 |  |  |  |
| 70.67 | 1393 |  |  |  |
| 70.983 | 1387.1 |  |  |  |
| 71.297 | 1386.8 |  |  |  |
| 71.605 | 1384.2 |  |  |  |
| 71.916 | 1384.7 |  |  |  |
| 72.228 | 1387.2 |  |  |  |
| 72.542 | 1390.8 |  |  |  |
| 72.855 | 1391.5 |  |  |  |
| 73.163 | 1385.2 |  |  |  |
| 73.475 | 1385.6 |  |  |  |
| 73.788 | 1393.1 |  |  |  |
| 74.102 | 1388.9 |  |  |  |
| 74.424 | 1390 |  |  |  |
| 74.726 | 1392.1 |  |  |  |
| 75.05 | 1389.8 |  |  |  |
| 75.364 | 1397.3 |  |  |  |
| 75.679 | 1389.4 |  |  |  |
| 75.991 | 1399.2 |  |  |  |
| 76.301 | 1396.5 |  |  |  |
| 76.613 | 1400 |  |  |  |
| 76.921 | 1397.2 |  |  |  |
| 77.233 | 1405.6 |  |  |  |
| 77.544 | 1399.3 |  |  |  |
| 77.859 | 1396.4 |  |  |  |
| 78.182 | 1404.6 |  |  |  |
| 78.501 | 1403.4 |  |  |  |
| 78.825 | 1401.4 |  |  |  |
| 79.143 | 1397.4 |  |  |  |
| 79.455 | 1400.4 |  |  |  |
| 79.784 | 1399.5 |  |  |  |
| 80.107 | 1409.2 |  |  |  |
| 80.425 | 1399 |  |  |  |
| 80.738 | 1407.1 |  |  |  |
| 81.051 | 1400.2 |  |  |  |
| 81.375 | 1401.9 |  |  |  |
| 81.688 | 1404.9 |  |  |  |
| 81.999 | 1401.4 |  |  |  |
| 82.311 | 1409.3 |  |  |  |
| 82.626 | 1402.9 |  |  |  |
| 82.944 | 1404.5 |  |  |  |
| 83.268 | 1406.1 |  |  |  |
| 83.581 | 1404.8 |  |  |  |
| 83.889 | 1407.4 |  |  |  |
| 84.199 | 1401.6 |  |  |  |
| 84.511 | 1407.6 |  |  |  |
| 84.825 | 1407.1 |  |  |  |
| 85.139 | 1410.2 |  |  |  |
| 85.467 | 1412.1 |  |  |  |
| 85.797 | 1408.7 |  |  |  |
| 86.11 | 1409.5 |  |  |  |
| 86.421 | 1410.2 |  |  |  |
| 86.733 | 1404.2 |  |  |  |
| 87.058 | 1412.3 |  |  |  |
| 87.371 | 1410 |  |  |  |
| 87.683 | 1407.1 |  |  |  |
| 87.994 | 1410.3 |  |  |  |
| 88.309 | 1411.5 |  |  |  |
| 88.622 | 1410 |  |  |  |
| 88.929 | 1412.3 |  |  |  |
| 89.245 | 1420.2 |  |  |  |
| 89.567 | 1412.3 |  |  |  |
| 89.884 | 1421.2 | 170.8 |  |  |
| 90.199 | 1408.6 | 163.9 |  |  |
| 90.523 | 1415.4 |  |  |  |
| 90.837 | 1414.3 |  |  |  |
| 91.151 | 1417.3 |  |  |  |
| 91.476 | 1415.4 |  |  |  |
| 91.792 | 1411 |  |  |  |
| 92.105 | 1419.8 |  |  |  |
| 92.434 | 1420.1 |  |  |  |
| 92.759 | 1416.8 |  |  |  |
| 93.078 | 1417.1 |  |  |  |
| 93.395 | 1419.7 |  |  |  |
| 93.715 | 1418.7 |  |  |  |
| 94.027 | 1413.4 |  |  |  |
| 94.338 | 1411.1 |  |  |  |
| 94.66 | 1415.7 |  |  |  |
| 94.979 | 1417.7 |  |  |  |
| 95.302 | 1416.7 |  |  |  |
| 95.622 | 1419.3 |  |  |  |
| 95.937 | 1422.4 |  |  |  |
| 96.246 | 1418.3 |  |  |  |
| 96.559 | 1418.2 |  |  |  |
| 96.885 | 1418.4 |  |  |  |
| 97.215 | 1416.8 |  |  |  |
| 97.529 | 1413.6 |  |  |  |
| 97.843 | 1416.3 |  |  |  |
| 98.161 | 1420.7 |  |  |  |
| 98.483 | 1422.2 |  |  |  |
| 98.801 | 1418.7 |  |  |  |
| 99.119 | 1422.7 |  |  |  |
| 99.439 | 1423.4 |  |  |  |
| 99.766 | 1421.9 |  |  |  |
| 100.08 | 1420.5 |  |  |  |
| 100.41 | 1417.5 |  |  |  |
| 100.73 | 1411.7 |  |  |  |
| 101.05 | 1418.9 |  |  |  |
| 101.37 | 1422.2 |  |  |  |
| 101.69 | 1414.4 |  |  |  |
| 102.01 | 1419.3 |  |  |  |
| 102.33 | 1418.6 |  |  |  |
| 102.65 | 1410.9 |  |  |  |
| 102.97 | 1414.5 |  |  |  |
| 103.29 | 1418.3 |  |  |  |
| 103.6 | 1420.7 |  |  |  |
| 103.92 | 1417.8 |  |  |  |
| 104.29 | 1423.3 |  |  |  |
| 104.67 | 1420.6 |  |  |  |
| 105.03 | 1413.6 |  |  |  |
| 105.42 | 1425.6 |  |  |  |
| 105.75 | 1425.3 |  |  |  |
| 106.06 | 1428.6 |  |  |  |
| 106.38 | 1427.1 |  |  |  |
| 106.7 | 1427.6 |  |  |  |
| 107.01 | 1428.9 |  |  |  |
| 107.33 | 1430.8 |  |  |  |
| 107.65 | 1423.5 |  |  |  |
| 107.96 | 1423 |  |  |  |
| 108.27 | 1422.6 |  |  |  |
| 108.59 | 1431.6 |  |  |  |
| 108.91 | 1428.2 |  |  |  |
| 109.23 | 1423.7 |  |  |  |
| 109.54 | 1429.1 |  |  |  |
| 109.85 | 1437.5 |  |  |  |
| 110.17 | 1427.2 |  |  |  |
| 110.51 | 1430.1 |  |  |  |
| 110.83 | 1431.3 |  |  |  |
| 111.15 | 1424.1 |  |  |  |
| 111.46 | 1426.5 |  |  |  |
| 111.78 | 1437.6 |  |  |  |
| 112.1 | 1433.9 |  |  |  |
| 112.42 | 1430 |  |  |  |
| 112.73 | 1432.8 |  |  |  |
| 113.05 | 1423 |  |  |  |
| 113.37 | 1431.6 |  |  |  |
| 113.68 | 1432.3 |  |  |  |
| 113.99 | 1425.3 |  |  |  |
| 114.3 | 1427.9 |  |  |  |
| 114.62 | 1429.9 |  |  |  |
| 114.94 | 1428.4 |  |  |  |
| 115.26 | 1424.8 |  |  |  |
| 115.58 | 1432.3 |  |  |  |
| 115.9 | 1429.9 |  |  |  |
| 116.21 | 1432.2 |  |  |  |
| 116.52 | 1426.4 |  |  |  |
| 116.83 | 1428.5 |  |  |  |
| 117.15 | 1427.6 |  |  |  |
| 117.46 | 1428 |  |  |  |
| 117.77 | 1429.9 |  |  |  |
| 118.09 | 1430.5 |  |  |  |
| 118.41 | 1430.6 |  |  |  |
| 118.72 | 1429.2 |  |  |  |
| 119.04 | 1429.9 |  |  |  |
| 119.35 | 1430.4 |  |  |  |
| 119.66 | 1426.8 |  |  |  |
| 119.98 | 1430.8 | 186.8 |  |  |
|  |  |  |  |  |
